# Prevalence and Estimated Economic Burden of Substandard and Falsified Medicines in Low- and Middle-Income Countries: A Systematic Review and Meta-analysis

**DOI:** 10.1001/jamanetworkopen.2018.1662

**Published:** 2018-08-10

**Authors:** Sachiko Ozawa, Daniel R. Evans, Sophia Bessias, Deson G. Haynie, Tatenda T. Yemeke, Sarah K. Laing, James E. Herrington

**Affiliations:** 1 Department of Maternal and Child Health, Gillings School of Global Public Health, University of North Carolina, Chapel Hill; 2 Division of Practice Advancement and Clinical Education, Eshelman School of Pharmacy, University of North Carolina, Chapel Hill; 3 Enterprise Analytics and Data Sciences, University of North Carolina Health Care, Chapel Hill; 4 University of Virginia School of Medicine, Charlottesville; 5 Department of Health Behavior, Gillings School of Global Public Health, University of North Carolina, Chapel Hill

## Abstract

**Importance:**

Substandard and falsified medicines burden health systems by diverting resources to ineffective or harmful therapies, causing medical complications and prolonging illnesses. However, the prevalence and economic impact of poor-quality medicines is unclear.

**Objective:**

To conduct a systematic review and meta-analysis to assess the prevalence and estimated economic burden of substandard and falsified essential medicines in low- and middle-income countries.

**Data Sources:**

Five databases (PubMed, EconLit, Global Health, Embase, and Scopus) were searched from inception until November 3, 2017.

**Study Selection:**

Publications were assessed to determine whether they examined medicine quality and the prevalence and/or economic burden of substandard and falsified medicines in low- and middle-income countries. Studies with a sample size of 50 or more were included in the meta-analysis.

**Data Extraction and Synthesis:**

The study is registered in PROSPERO and reported via the Preferred Reporting Items for Systematic Reviews and Meta-analyses (PRISMA) reporting guidelines. Study quality was assessed using an adapted Medicine Quality Assessment Reporting Guidelines scoring metric. Multiple reviewers conducted the data extraction and quality assessment independently.

**Main Outcomes and Measures:**

Prevalence and/or estimated economic impact of substandard and falsified medicines.

**Results:**

Two hundred sixty-five studies that estimated the prevalence of poor-quality essential medicines in low- and middle-income countries were identified. Among 96 studies that tested 50 samples or more (67 839 total drug samples), overall prevalence of poor-quality medicines was 13.6% (95% CI, 11.0%-16.3%), with regional prevalence of 18.7% in Africa (95% CI, 12.9%-24.5%) and 13.7% in Asia (95% CI, 8.2%-19.1%). Of studies included in the meta-analysis, 19.1% (95% CI, 15.0%-23.3%) of antimalarials and 12.4% (95% CI, 7.1%-17.7%) of antibiotics were substandard or falsified. Eight approximations of the economic impact, focused primarily on market size, with poor or undisclosed methods in estimation were identified, ranging from $10 billion to $200 billion.

**Conclusions and Relevance:**

Poor-quality essential medicines are a substantial and understudied problem. Methodological standards for prevalence and rigorous economic studies estimating the burden beyond market size are needed to accurately assess the scope of the issue and inform efforts to address it. Global collaborative efforts are needed to improve supply-chain management, surveillance, and regulatory capacity in low- and middle-income countries to reduce the threat of poor-quality medicines.

**Trial Registration:**

PROSPERO Identifier: CRD42017080266

## Key Points

**Question:**

What are the prevalence and estimated economic burden of substandard and falsified medicines in low- and middle-income countries?

**Findings:**

In this systematic review of 265 studies comprising 400 647 drug samples and meta-analysis of 96 studies comprising 67 839 drug samples, the prevalence of substandard and falsified medicines in low- and middle-income countries was 13.6% overall (19.1% for antimalarials and 12.4% for antibiotics). Data on the estimated economic impact were limited primarily to market size and ranged widely from $10 billion to $200 billion.

**Meaning:**

Substandard and falsified medicines are a substantial health and economic problem; a concerted global effort is needed to secure the global supply chain, increase quality control capacity, and improve surveillance to better assess the problem and identify solutions.

## Teaser

This systematic review and meta-analysis examines 96 studies of substandard and falsified medicines in low- and middle-income countries to determine the prevalence and estimated economic burden of these medicines.

## Introduction

Increasing access to essential medicines is integral to the effort to reduce global morbidity and mortality.^1^ While access and health outcomes have generally improved in recent decades, these efforts face a serious obstacle from the threat of substandard and falsified (SF) medicines.^2,3,4^ Poor-quality medicines increase risks of morbidity and mortality by prolonging illnesses and heighten the risk of treatment failure, poisoning, and adverse drug interactions.^5,6^ Circulation of SF medicines with little clinical effectiveness also places entire communities at risk of drug resistance, posing a threat to global treatment effectiveness, as well as undermining people’s overall trust in the health system and its legitimate health care professionals.^5,6,7,8,9^ Over time, diminished trust in licensed health care professionals may further encourage informal care-seeking and self-medication.^10^ Poor health outcomes can also erode trust in the manufacturers of genuine pharmaceutical products, which are often challenging to distinguish from SF ones without the use of verification technologies.^11^

The risks posed by these medicines extend beyond health outcomes.^6^ Poor-quality medicines cause increased costs for patients and the health system.^5,6^ Some of these costs, such as resources wasted on ineffective therapies and treating additional complications, are borne primarily by consumers and health facilities.^5,6^ Others, such as decreased economic productivity resulting from prolonged illness, reduced sales and tax revenue, and costs of anticounterfeiting initiatives, are borne by governments, companies, the pharmaceutical industry, donors, and society as a whole.^5,6,12^ Furthermore, SF medicines purchased through the use of personal savings, sale of assets, or borrowing can lead people into poverty.^5^

Substandard and falsified medicines are a complex but critical global health issue.^3,5,6,9^ The World Health Organization (WHO) estimates that 10.5% of medicines worldwide are substandard or falsified.^5^ Furthermore, most of the burden falls on low- and middle-income countries (LMICs) because of poor pharmaceutical governance, weak technical capacity, and poor supply-chain management.^6,13^ Until recently, the efforts to combat SF medicines have been fragmented because of the complexity of the issue and intellectual property rights disputes.^14^ In 2017, in an effort to draw the issue away from intellectual property rights and reframe it solely as a public health issue, the World Health Assembly officially adopted the term *substandard and falsified* to replace the previous term, *substandard/spurious/falsely labeled/falsified/counterfeit (SSFFC).*^15^ Substandard medicines are officially defined as “authorized medical products that fail to meet either their quality standards or specification, or both” and may result through poor manufacturing, shipping or storage conditions, or when the drug is sold beyond the expiration date.^14^ Falsified medicines are defined as “medical products that deliberately/fraudulently misrepresent their identity, composition or source.”^14^

This issue not only has significant health and economic consequences, but directly threatens global health security and efforts to meet the United Nations Sustainable Development Goal 3.8, to achieve universal access to safe and effective essential medicines.^1,5^ Despite this, the full extent of the problem is unclear.^5,6^ Furthermore, research efforts to examine the issue have often faced poor methodological quality and exhibited high amounts of variability.^16^ To address these issues, we systematically reviewed and analyzed the existing evidence to assess the prevalence and estimated economic burden of SF essential medicines across LMICs.

## Methods

For this systematic review and meta-analysis, 5 databases were searched: PubMed, EconLit, Global Health, Embase, and Scopus. A research librarian was consulted to aid in the creation of 2 separate searches and ensure all relevant studies were identified. The first search used terms related to “substandard and falsified medicines” (incorporating SSFFC terms) and the second, “quality of medicine.” Both searches were run with terms associated with “low- and middle-income countries.” Additional articles were incorporated through systematic searches of the WHO Essential Medicines and Health Products Information Portal,^17^ the United States Pharmacopeial Convention Medicines Quality Database,^18^ publications in the Worldwide Antimalarial Resistance Network database,^19^ and references of included articles and pertinent literature reviews.^3,5,20,21,22,23,24,25,26,27,28,29^ The comprehensive search strategy and terminology as well as a list of additional sources searched are presented in eAppendix 1 in the Supplement. This systematic review and meta-analysis is registered in the PROSPERO database and is reported according to the Preferred Reporting Items for Systematic Reviews and Meta-analyses (PRISMA) reporting guidelines. Institutional review board approval was not required as this study did not involve human subjects research.

The primary eligibility criteria for this systematic review were that the article examined the quality of essential medicines, the prevalence of SF medicines, and/or the economic impact of SF medicines. Economic impact is defined broadly as any economic estimate associated with the manufacture, trade, sale, or consumption of SF medicines. Peer-reviewed articles in English, Chinese, French, Portuguese, Spanish, or German published before November 3, 2017, were included in the review. All study locations not in LMICs, as classified as the World Bank at the time of review, were excluded. Abstract-only publications, correspondence without data, studies of medicines that are not classified as essential medicines by the WHO,^30^ case reports, and publications that did not include sample sizes of tested medicines were also excluded.

All unique articles were independently assessed by 3 of us (D.G.H., S.B., and D.R.E.) based on title and abstract. Those marked for inclusion, or whose title and abstract were not sufficient to determine inclusion, were then reviewed using the full text. Any discrepancies between reviewers were resolved by a third party (S.O.). Full-text records were sought and obtained through the library systems of 3 major US research universities. Selected articles were then categorized into 1 or both of 2 groups: (1) primary prevalence studies and/or (2) articles containing an estimate of economic impact.

Four of us (D.G.H., S.B., S.K.L., and D.R.E.) independently conducted the data extraction with oversight by a third party (S.O.). Data describing prevalence, type of medicines, country setting, and sample size were extracted from all included primary prevalence studies. Studies were grouped according to the following categories: type of medicines tested, sample size, continent, and year of publication.

Studies including an estimate of economic impact were compiled separately and data were extracted on estimate source, period of analysis, geographic scope, target medicine, and estimated economic impact. Citation mining was conducted to determine the root sources of the cited statistics. For every estimate of economic impact, we noted the root source, type of literature, and methods used, or recorded that the root source was untraceable if it could not be traced back.

The meta-analysis of the prevalence of SF medicines focused on studies that tested 50 samples or more and adequately reported sampling and testing methods to ensure the rigor of prevalence estimates. Studies that did not report primary data, included previously reported data, or included data from regulatory laboratories that only tested suspected medicines were excluded from the meta-analysis. Where available, uncertainty ranges or minimum to maximum prevalence were noted alongside summary statistics. Mean prevalence across studies—weighted by sample size and Medicine Quality Assessment Reporting Guidelines (MEDQUARG) score^16^ using a random-effects model—was determined overall and by world region. Mean weighted prevalence was also assessed across subcategorizations of 8 study characteristics: sample size, publication year, sampling method, purchasing method, chemical analysis, chemical testing method, conflict of interest, and MEDQUARG score. The prevalence for each subcategorization was compared with the overall prevalence with a 1-sample *t* test and with the subgroup gold standard by an unequal variances *t* test. Overall uncertainty ranges were estimated using a 95% confidence interval based on the calculated standard deviation.

Additional data were extracted for these studies in the meta-analysis, including methods of sampling, purchasing, and pharmaceutical quality analysis. Sampling methods were categorized as random, convenience, survey-based, or not specified; purchasing method was categorized by use of mystery clients, using overt methods, or not specified; and pharmaceutical quality analysis was categorized by the specific chemical analyses. Additionally, each study was assessed to determine whether it (1) examined the actual presence of active pharmaceutical ingredients (APIs), (2) assessed the quantity or percentage of the APIs, and/or (3) looked for other ingredients (excipients and other analytes). Use of Raman spectrometry, any mention of impurities, or highlighting unknown peaks in chromatographs were counted as searching for other ingredients.

To assess the quality of the studies included in the meta-analysis, studies were independently examined by 2 of us (D.R.E. and S.K.L.) according to MEDQUARG.^16^ Each study was assigned a 12-point MEDQUARG score adapted by Almuzaini and colleagues^20^ with scores of 6 or greater being considered acceptable quality. The interrater reliability was assessed between the 2 reviewers and, where possible, the original Almuzaini scores using the Spearman ρ. Additional information about the MEDQUARG scoring metric, this study’s reported MEDQUARG scores (eTable 1 in the Supplement), and results of the interrater reliability assessments are presented in eAppendix 2 in the Supplement. Additionally, studies included in the meta-analysis were analyzed for potential conflict of interest by examining the articles’ author statements, funding source, and/or institutional affiliations for a direct partisan or industry relationship. Studies with a description of funding source with no apparent partisan or industry affiliation were considered free of potential conflict of interest. Other studies that did not include a funding source and had no discernable industry author affiliations were categorized as unclear.

Study heterogeneity was evaluated using a random-effects model and reported using the Cochran *Q* and *I*^2^. Publication bias was evaluated using a funnel plot analysis (eFigure 1 in the Supplement) with a regression test for funnel plot asymmetry. Baujaut and influence plot analyses (eFigures 2 and 3 in the Supplement) were conducted to examine which articles contributed the most heterogeneity. A mixed-effects model was used to test for potential modifiers. The results of these analyses are included in eAppendix 3 in the Supplement. All analyses were made using R statistical software version 3.3.2 (R Project for Statistical Computing).^31^

## Results

### Systematic Review

**Figure 1.**
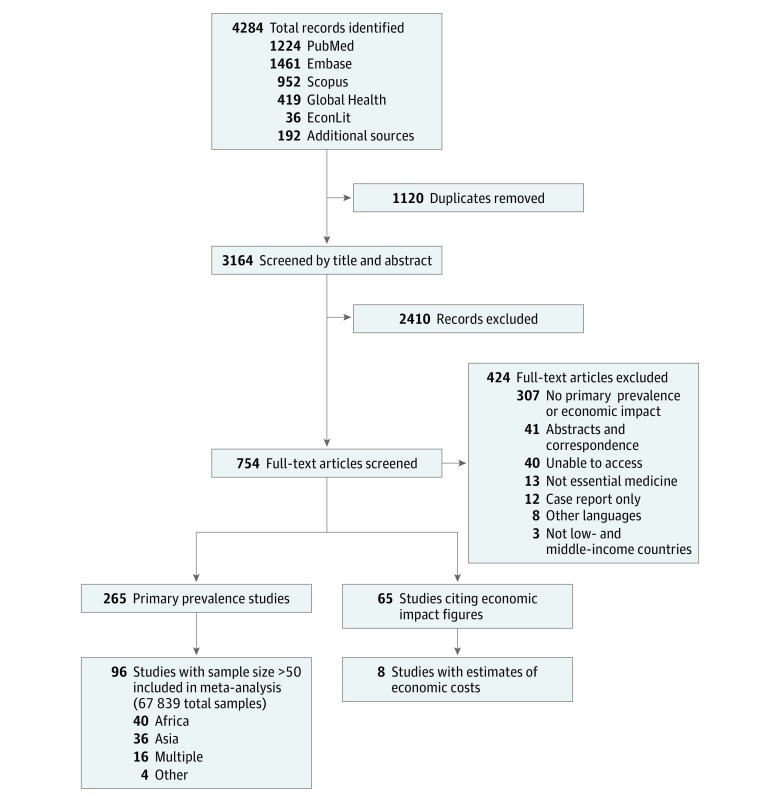
PRISMA Flowchart

Our searches yielded a total of 4284 citations, of which 3164 were unique and screened based on title and abstract. The full text was assessed for 754 articles. We identified 265 primary data collection studies that sought to determine the prevalence of SF essential medicines in LMICs (Figure 1). Studies not included in the meta-analysis are shown in eReferences 1 in the Supplement.

Globally, data on SF medicines in LMICs came primarily from articles focused on Africa (133 studies [50.2%]) and Asia (90 studies [34.0%]). Eight studies (3.0%) covered countries in South America, while 3 studies (1.1%) tested samples from each of Europe, North America, and Oceania. Twenty-five studies (9.4%) tested samples from multiple continents. More than half of the 265 prevalence studies (157 [59.2%]) were published in this decade (2010-present) with 63 (23.8%) published in the last 3 years (2015-2017). Antimalarials (117 studies [44.2%]) and antibiotics (104 studies [39.2%]) were the most commonly examined medicines, including 35 studies (13.2%) that investigated both. The total number of samples tested was 400 647, with a median (interquartile range) study sample size of 41 (10-242) (see eTable 2 in the Supplement for additional details on study characteristics).

### Meta-analysis

**Figure 2.**
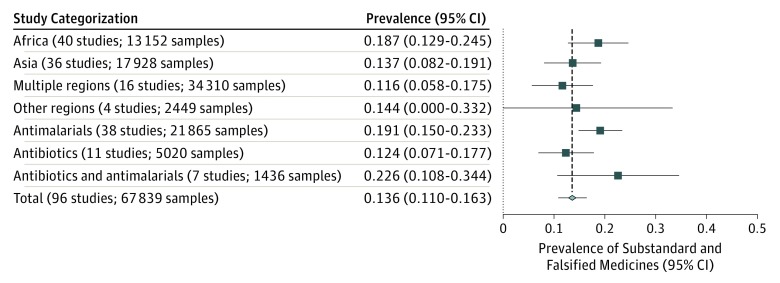
Prevalence of Substandard and Falsified Medicines in Low- and Middle-Income Countries by Medicine Category and Region The forest plot presents mean prevalence by study category among studies that only examined specific medicines.

**Figure 3.**
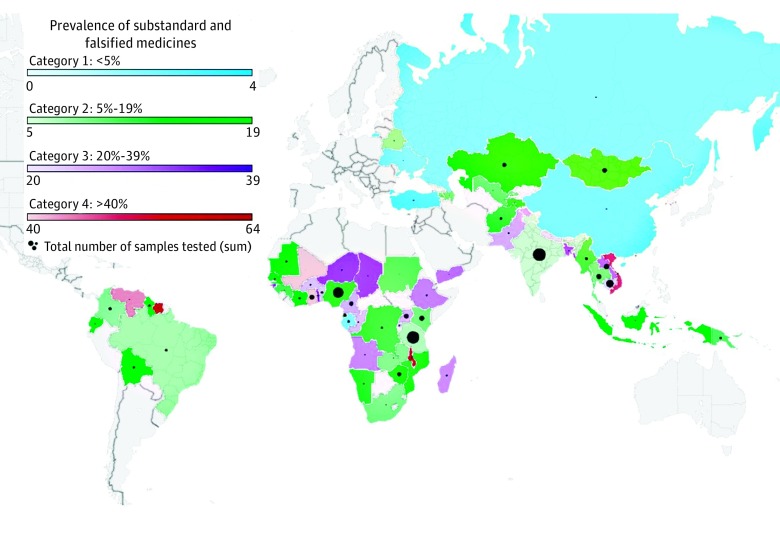
Reported National Prevalences of Substandard and Falsified Medicines Results of studies included in the meta-analysis. Multicountry studies that did not report country-specific data were not included. Subcategorical prevalence is delineated by color (blue, green, purple, and red as categories 1 through 4), and by color gradation, with a darker color representing a higher prevalence. Total number of samples tested for each country is presented as a black circle with the diameter of the circle increasing proportionally to samples tested. This map was generated using study data and the Microsoft Excel 2016 3D Mapping tool.

Our meta-analysis included a subset of 96 studies^32,33,34,35,36,37,38,39,40,41,42,43,44,45,46,47,48,49,50,51,52,53,54,55,56,57,58,59,60,61,62,63,64,65,66,67,68,69,70,71,72,73,74,75,76,77,78,79,80,81,82,83,84,85,86,87,88,89,90,91,92,93,94,95,96,97,98,99,100,101,102,103,104,105,106,107,108,109,110,111,112,113,114,115,116,117,118,119,120,121,122,123,124,125,126,127^ (67 839 drug samples) that met inclusion criteria. Figure 2 shows the results of the meta-analysis of the prevalences grouped by region and medication category. The average overall prevalence of SF medicines was 13.6% across LMICs (95% CI, 11.0%-16.3%). Regional prevalence estimates ranged from 18.7% in Africa (95% CI, 12.9%-24.5%) to 13.7% in Asia (95% CI, 8.2%-19.1%) and 14.4% (95% CI, 0%-33.2%) for other single-region studies. Studies that tested samples across multiple continents observed a lower average prevalence, with a pooled prevalence of 11.6% (95% CI, 5.8%-17.5%). The average prevalence of SF medicines was 19.1% (95% CI, 15.0%-23.3%) for antimalarials and 12.4% (95% CI, 7.1%-17.7%) for antibiotics. Table 1 presents the data extracted from these studies grouped by medication class. A map of the calculated national prevalence of SF medicines is presented in Figure 3 (eFigures 4-7 in the Supplement present the reported prevalence of SF medicines by each study in the meta-analysis grouped by region).

**Table 1.** Studies on the Prevalence of Substandard and Falsified Medicines by Medication Type

Source	Countries	Samples, No.	Substandard and Falsified Medicines, % (95% CI)	Sampling Method	Purchasing Method	Testing Method	Tested % Active Pharmaceutical Ingredient	Tested Other Ingredients
**Antibiotics**								
Bate et al,^39^ 2012	Angola, Brazil, China, Democratic Republic of the Congo, Egypt, Ethiopia, Ghana, India, Kenya, Mozambique, Nigeria, Russia, Rwanda, Tanzania, Thailand, Turkey, Uganda, Zambia	1437	4.95 (4.1-5.8)	Random	Mystery client	GPHF-Minilab^a^	Yes	NS
Bate et al,^41^ 2014^c^	Angola, Democratic Republic of the Congo, Egypt, Ethiopia, Ghana, Kenya, Nigeria, Rwanda, Tanzania, Uganda, Zambia, India, Thailand, China, Turkey, Russia, Brazil, Mozambique	1470	5.45 (3.9-7)	Random	Mystery client	GPHF-Minilab^a^	Yes	NS
Bate et al,^40^ 2015	Angola, Democratic Republic of the Congo, Egypt, Ethiopia, Ghana, Kenya, Nigeria, Rwanda, Tanzania, Uganda, Zambia, India, Thailand, China, Turkey, Russia, Brazil, Mozambique	1437	9.88	Random	Mystery client	GPHF-Minilab^a^	Yes	NS
Hadi et al,^52^ 2010	Indonesia	104	18	Convenience	Mystery client	HPLC	Yes	NS
Khan et al,^64^ 2013	India	59	33.9	Random	Mystery client	Visual examination, HPLC, UV, dissolution, other^b^	Yes	NS
Kitutu; Uganda Medicines Transparency Alliance,^114^ 2015	Uganda	179	14	Random	NS	GPHF-Minilab^a^	Yes	NS
Khurelbat et al,^68^ 2014^c^	Mongolia	1236	14.64 (13.2-17.8)	Random	Mystery client	Disintegration, dissolution, TLC, UV, IR, other^b^	Yes	NS
Kyriacos et al,^70^ 2008	Lebanon, Jordan, Egypt, Saudi Arabia	111	56	Random	NS	HPLC	Yes	NS
Nabirova et al,^77^ 2017	Kazakhstan	854	19.1	Random	NS	GPHF-Minilab^a^	Yes	Yes
Nazerali and Hogerzeil,^78^ 1998^c^	Zimbabwe	840	16	Convenience	NS	Disintegration, other^b^	Yes	NS
Obaid et al,^81^ 2009	Pakistan	96	15.6	Random	NS	HPLC	Yes	NS
Okumura et al,^85^ 2010	Cambodia	254	8.7	Convenience	NS	Dissolution	No	No
Patel et al,^88^ 2012^c^	South Africa	135	8.9	Convenience	Overt	Visual inspection, dissolution, other^b^	Yes	Yes
Ramachandran et al,^94^ 2013^c^	India	1948	8.5	Convenience	NS	Other^b^	Yes	NS
Rookkapan et al,^96^ 2005	Thailand	198	25.3	Random	Overt	HPLC, UV, dissolution	Yes	NS
Sabartova et al,^98^ 2011	Armenia, Azerbaijan, Belarus, Estonia, Kazakhstan, Latvia, Moldova, Ukraine, Uzbekistan	291	11.3	Convenience	NS	Visual inspection, dissolution, HPLC, other^b^	Yes	Yes
Wafula et al,^117^ 2016^c^	Kenya	60	17	Convenience	Mystery client	HPLC, dissolution, disintegration, other^b^	Yes	NS
Yoshida et al,^127^ 2014^c^	Cambodia	325	14.5	Random	Mystery client	HPLC, UV, dissolution, other^b^	Yes	NS
Yusuf et al^118^ 2011^c^	Afghanistan	348	9.5	Convenience	Overt	Other^b^	NS	NS
**Antimalarials**								
Abdo-Rabbo et al,^32^ 2005	Yemen	50	28	Random, convenience	NS	Dissolution, HPLC, UV	Yes	NS
ACT Consortium,^33^ 2015	Tanzania	1737	4.1	Random	Overt	HPLC, MS, UV	Yes	NS
Amin et al,^34^ 2005	Kenya	116	39.15 (33-45.3)	Convenience	Overt	Dissolution, HPLC, UV	Yes	NS
Basco,^37^ 2004	Cameroon	284	39.44 (12-74)	NS	Mystery client	Colorimetry, TLC	Yes	Yes
Bjorkman et al,^43^ 2012	Uganda	558	19.4	Random	Mystery client	Raman spectrometry, visual inspection	Yes	Yes
Chikowe et al,^46^ 2015	Malawi	112	88.4	Random, convenience	Mystery client, overt	Visual inspection, colorimetry, TLC, HPLC	Yes	NS
Dondorp et al,^47^ 2004	Myanmar, Lao People’s Democratic Republic, Vietnam, Cambodia, Thailand	232	44.4 (9-53)	Convenience	Mystery client	Colorimetry, HPLC, UV	Yes	NS
Evans et al,^48^ 2012	Guyana, Suriname	135	70.4	Convenience	Mystery client, overt	Visual inspection, dissolution, disintegration, other^b^	No	NS
Gimenez et al,^50^ 1997	Cambodia	128	28	Convenience	Mystery client	TLC, dissolution, HPLC, other^b^	Yes	NS
Guo et al,^51^ 2017	Myanmar	153	0.7	Convenience	NS	Colorimetry, HPLC	Yes	NS
Idowu et al,^56^ 2006	Nigeria	50	38	NS	NS	Visual inspection, colorimetry	No	NS
Ioset and Kaur,^57^ 2009	13 Countries in Asia, South America, and Africa, including Kenya, Nigeria, Vietnam (does not name all 13)	171	1.33	NS	NS	GPHF-Minilab,^a^ colorimetry, HPLC, UV	Yes	Yes
Kaur et al,^60^ 2016	Equatorial Guinea, Cambodia, Ghana, Nigeria, Rwanda, Tanzania	10079	19.3 (1.6-37)	Random	Mystery client, overt	HPLC, MS	Yes	Yes
Kaur et al,^61^ 2008	Tanzania	304	12.2 (7.5-23.8)	Random	NS	Dissolution, HPLC	Yes	NS
Kenyan Ministry of Public Health and Sanitation,^62^ 2011	Kenya	451	8.2	Survey	Mystery client	GPHF-Minilab,^a^ dissolution, other^b^	Yes	Yes
Kenyan Ministry of Public Health and Sanitation,^63^ 2012	Kenya	496	3	Survey	Mystery client	GPHF-Minilab,^a^ other^b^	Yes	Yes
Khin et al,^66^ 2016	Myanmar	51	23.5	Random	NS	GPHF-Minilab,^a^ dissolution, HPLC, other^b^	Yes	NS
Lalani et al,^71^ 2015	Afghanistan	134	26	Random	Mystery client, overt	GPHF-Minilab,^a^ dissolution, HPLC, UV	Yes	NS
Lon et al,^74^ 2006	Cambodia	451	27.1	Convenience	Mystery client, overt	GPHF-Minilab,^a^ HPLC	Yes	NS
Maponga and Ondari,^75^ 2003	Gabon, Ghana, Kenya, Mali, Mozambique, Sudan, Zimbabwe	288	21.5	Convenience	NS	HPLC, UV, dissolution	Yes	Yes
Newton et al,^79^ 2001	Cambodia, Lao People’s Democratic Republic, Myanmar, Thailand, Vietnam	104	38	Convenience	NS	Colorimetry, visual inspection	No	NS
Newton et al,^80^ 2008	Vietnam, Cambodia, Lao People’s Democratic Republic, Myanmar, Thailand	391	49.9	Random, convenience	NS	Visual inspection, colorimetry, HPLC, MS	Yes	Yes
Ochekpe et al,^82^ 2010	Nigeria	70	44.3	Random	NS	GPHF-Minilab^a^	Yes	Yes
Ogwal-Okeng et al,^83^ 1998	Uganda	88	59.1	Random	Mystery client	HPLC	Yes	NS
Ogwal-Okeng et al,^84^ 2003	Uganda	92	44.6	Random	Mystery client	HPLC	Yes	NS
Onwujekwe et al,^86^ 2009	Nigeria	225	26.67	Random	Mystery client, overt	Dissolution, HPLC	Yes	NS
Osei-Safo et al,^87^ 2014	Ghana, Togo	124	75.8	Convenience	Mystery client	Visual inspection, colorimetry, TLC, HPLC	Yes	NS
Phanouvong et al,^90^ 2013b	Thailand	709	1	Random	Mystery client, overt	GPHF-Minilab,^a^ dissolution, other^b^	Yes	NS
Phanouvong et al,^91^ (2013a)	Cambodia	374	12.3	Random	Mystery client, overt	GPHF-Minilab,^a^ dissolution, other^b^	Yes	NS
Pribluda et al,^93^ 2012	Bolivia, Brazil, Colombia, Ecuador, Guyana, Suriname, Venezuela	1663	11.6	Convenience	NS	GPHF-Minilab^a^	Yes	NS
Tabernero et al,^108^ 2015	Lao People’s Democratic Republic	158	25.4	Random	Mystery client	HPLC, MS, UV, visual inspection	Yes	Yes
Tipke et al,^110^ 2008	Burkina Faso	77	41.6	Random, convenience	Mystery client	GPHF-Minilab,^a^ UV	Yes	Yes
Tivura et al,^111^ 2016	Ghana	254	35.4	Random	Mystery client	HPLC, MS, UV	Yes	NS
Vijaykadga et al,^115^ 2006	Thailand	369	11.39 (11.1-29.4)	Survey	Overt	GPHF-Minilab,^a^ HPLC	Yes	NS
Visser et al,^116^ 2015	Gabon	432	3.7	Random	Mystery client	GPHF-Minilab,^a^ HPLC, UV	Yes	NS
World Health Organization,^122^ 2009	Madagascar, Senegal, Uganda	197	32.5	Convenience	Mystery client	GPHF-Minilab,^a^ other^b^	Yes	NS
World Health Organization,^123^ 2011	Cameroon, Ethiopia, Ghana, Kenya, Nigeria, Tanzania	267	28.5	Random	NS	GPHF-Minilab,^a^ HPLC, other^b^	Yes	Yes
Yeung et al,^126^ 2015	Cambodia	291	31.3 (25.8-50)	Random, survey	Mystery client, overt	HPLC, MS, UV	Yes	NS
**Antimalarials and Antibiotics**								
Baratta et al,^36^ 2012^c^	Congo, Ethiopia, India, Malawi, Central African Republic, Guinea Conakry, Uganda, Brazil, Guinea Bissau, Madagascar, Kenya, Angola, Rwanda, Cameroon, Chad	221	52	NS	NS	HPLC, UV, other^b^	Yes	Yes
Bate et al,^42^ 2010^c^	Ghana, Tanzania, Uganda, Nigeria, Angola, Zambia, Kenya, India, Thailand, China, Turkey, Russia, Brazil	2065	10.82 (7.3-14.2)	NS	Mystery client	GPHF-Minilab^a^	Yes	NS
Bruneton,^44^ 1995^c^	Cameroon, Madagascar, Chad	429	31.2	NS	Mystery client	HPLC, TLC, UV, other^b^	Yes	Yes
Central Drugs Standard Control Organization,^45^ 2009^c^	India	2976	0.1	Survey	Mystery client	Visual inspection, other^b^	NS	NS
Hajjou et al,^53^ 2015^c^	Ghana, Ethiopia, Liberia, Kenya, Mozambique, Cambodia, Indonesia, Lao People’s Democratic Republic, Myanmar, Philippines, Thailand, Vietnam, China, Colombia, Ecuador, Guyana, Peru	15063	5.6 (2.9-11.5)	Convenience	NS	GPHF-Minilab,^a^ HPLC	Yes	Yes
Hetzel et al,^55^ 2014	Papua New Guinea	360	10.1	Survey, random, convenience	Overt	HPLC, UV, MS	Yes	NS
Kaale et al,^58^ 2016^c^	Tanzania	242	6.2	Random	Mystery client	GPHF-Minilab,^a^ HPLC	Yes	NS
Khan et al,^65^ 2011^c^	Cambodia	679	4.6	Random	Mystery client	HPLC, dissolution	Yes	NS
Khuluza et al,^67^ 2017	Malawi	56	12.5	Random	Mystery client, overt	GPHF-Minilab,^a^ dissolution, other^b^	Yes	Yes
Petersen et al,^89^ 2017^c^	Cameroon, Democratic Republic of the Congo, India, Ghana, Kenya, Nigeria, Uganda	869	2.4	Convenience	Mystery client, overt	GPHF-Minilab,^a^ dissolution, HPLC, other^b^	Yes	NS
Pouillot et al,^92^ 2008	Niger, Cameroon	153	45.75	Random	Overt	HPLC, UV, dissolution	Yes	NS
Risha et al,^95^ 2008^c^	Tanzania	1257	3.7	Convenience	Overt	GPHF-Minilab,^a^ dissolution	Yes	NS
Seear et al,^99^ 2011	India	300	43	Random	Overt	HPLC, MS	Yes	NS
Shakoor et al,^100^ 1997	Nigeria, Thailand	96	36.5 (36-40)	Random	Mystery client	HPLC	Yes	Yes
Sheth et al,^101^ 2007^c^	India	2455	0.3	Convenience	Mystery client	Visual inspection, other^b^	Yes	NS
Stenson et al,^104^ 1998	Lao People’s Democratic Republic	366	11.5 (3.3-46.2)	Random	NS	TLC, UV, colorimetry, HPLC, other^b^	Yes	NS
Syhakhang,^106^ 2002^c^	Lao People’s Democratic Republic	666	46	Random	Mystery client	TLC, UV, colorimetry, HPLC, other^b^	Yes	NS
Syhakhang et al,^107^ 2004^c^	Lao People’s Democratic Republic	300	5.33	Random	Mystery client	Disintegration, HPLC, UV, colorimetry, other^b^	Yes	NS
Taylor et al,^109^ 2001^c^	Nigeria	581	48	Random	Mystery client	HPLC	Yes	NS
Tshilumba et al,^112^ 2015^c^	Democratic Republic of Congo	60	31.7	Convenience	NS	Visual inspection	NS	NS
Uganda Medicines Transparency Alliance,^113^ 2014	Uganda	105	4.8	Random	NS	GPHF-Minilab^a^	Yes	Yes
Wondemagegnehu,^120^ 1999^c^	Myanmar, Vietnam	500	11.2	Random	Mystery client	Other^b^	Yes	NS
**Other**^c^								
Antignac et al,^35^ 2017	Benin, Burkina Faso, Congo, the Democratic Republic of Congo, Guinea, Côte d'Ivoire, Mauritania, Niger, Senegal, Togo	1530	16.3	Random	Mystery client	MS, other^b^	Yes	NS
Bate et al,^38^ 2013	Angola, Democratic Republic of the Congo, Egypt, Ethiopia, Ghana, Kenya, Nigeria, Rwanda, Tanzania, Uganda, Zambia, India, Thailand, China, Turkey, Russia, Brazil	713	9.1 (3.9-16.6)	Random	Mystery client	GPHF-Minilab^a^	Yes	NS
Fotiou et al,^49^ 2009	Thailand	139	23.02	Convenience	Mystery client	HPLC, MS, other^b^	Yes	Yes
Hall,^54^ 2016	Bangladesh, Egypt, Cambodia, Kenya, India, Mexico, Nigeria, Pakistan, Peru, Viet Nam, Nigeria, Nepal, Pakistan, Bangladesh, Argentina, Indonesia, Peru, the Philippines, Kazakhstan	215	45	Random, convenience	NS	Visual inspection, HPLC, UV	Yes	Yes
Karikari-Boateng,^59^ 2013	Ghana	279	63.8	Survey	Overt	Other^b^	NS	Yes
Kuwana and Sabartova,^69^ 2017	Burkina Faso, Democratic Republic of the Congo, Nigeria, Rwanda, Zambia	126	0.8	Convenience	NS	Other^b^	NS	NS
Laroche et al,^72^ 2005	Mauritania	146	13.7 (8.8-20)	Random, Convenience	NS	HPLC, disintegration, IR, other^b^	Yes	Yes
Laserson et al,^73^ 2001	Colombia, Estonia, India, Latvia, Russia, Vietnam	71	10	Convenience	NS	TLC, UV	Yes	Yes
Mbaziira et al,^76^ 2015	Namibia	151	13.9	NS	NS	Other^b^	NS	NS
Roy et al,^97^ 1993	Bangladesh	53	30.2	Random	NS	HPLC, UV, disintegration, dissolution, other^b^	Yes	Yes
Stanton et al,^102^ 2012	Ghana	101	89.1 (76.09-100)	Random	Mystery client	HPLC	Yes	NS
Stanton et al,^103^ 2014	India	381	53.8	Convenience	Mystery client	Other^b^	Yes	NS
Suleman et al,^105^ 2014	Ethiopia	106	45.3	Random	Mystery client	HPLC, dissolution, visual inspection, other^b^	Yes	NS
Wang et al,^119^ 2015	8 Countries and 5 internet pharmacies: South Africa, United States, China, Ethiopia, Thailand, Lao People’s Democratic Republic, Mexico, Nigeria	88	6.82	Random	NS	HPLC, UV, dissolution, other^b^	Yes	NS
World Health Organization,^121^ 2007	Cameroon, Democratic Republic of the Congo, Kenya, Nigeria, Tanzania, Uganda, Zambia	394	1.8	Convenience	NS	Visual inspection, dissolution, disintegration, other^b^	Yes	Yes
World Health Organization,^124^ 2016	Burkina Faso, Kenya, Madagascar, Nepal, Nigeria, Tajikistan, Tanzania, Uganda, Vietnam, Zimbabwe	204	23	Convenience	NS	Visual inspection, other^b^	Yes	NS
Yang et al,^125^ 2004	Cambodia	96	92.7	Random	Mystery client	Disintegration, dissolution, other^b^	No	NS

Abbreviations: GPHF-Minilab, Global Pharma Health Fund-Minilab; HPLC, high-performance liquid chromatography; IR, infrared spectrometry; MS, mass spectrometry; NS, not specified; TLC, thin-layer chromatography; UV, ultraviolet and visible spectroscopy.

^a^ GPHF-Minilab involves visual inspection, disintegration, and TLC.

^b^ Other testing methods include infrared spectroscopy, uniformity of mass, microbial load, etc.

^c^ These studies examined the quality of medicines beyond antibiotics and antimalarials, such as acid blockers, antacids, anthelmintics, antianemics, antimycobacterials, antifungals, antihypertensives, anti-inflammatories, antiretrovirals, bronchodilators, erectile dysfunction drugs, diuretics, spasmolytics, and steroids.

Table 2 presents the summary statistics and weighted prevalence of the subcategorizations of 8 study characteristics in the meta-analysis. Statistical analysis of the association between the mean SF prevalence of each subcategorization and the overall prevalence (13.6%) found studies with smaller sample sizes to report significantly higher mean prevalence (sample size 50-99: mean prevalence, 34.4%; *P* = .001 and sample size 100-249: mean prevalence, 31%; *P* < .001). Conversely, the prevalence of studies that used convenience sampling (7.1%; *P* = .001) or survey sampling (2.3%; *P* = .009) and those that analyzed samples with the Global Pharma Health Fund’s Minilab, a mobile minilaboratory suitcase, were found to be significantly lower (7.7%; P < .001) than the overall prevalence of 13.6%.

**Table 2.** Prevalence of Substandard and Falsified Medicines by Study Characteristics

Category	Studies, No. (%)	Samples, No. (%)	Prevalence (95% CI)	*P* Value	
				Overall	Subgroup
Sample size					
50-99	17 (18)	1213 (1.8)	34.4 (23.2-45.6)	.001^a^	<.001^a^
100-249	30 (31)	4625 (6.8)	31.0 (22.9-39.0)	<.001^a^	<.001^a^
250-499	25 (26)	8679 (12.8)	20.6 (13.6-27.5)	.05	.001^a^
500-999	10 (10)	6969 (10.3)	17.0 (6.2-27.8)	.50	.06
1000-9999^b^	12 (13)	21 211 (31.3)	7.0 (3.7-10.3)	.001^a^	NA
≥10 000^c^	2 (2)	25 142 (37.1)	11.9 (0-32.7)	.79	NA
Publication year					
2015-2017^b^	25 (26)	34 535 (50.9)	12.4 (9.0-15.9)	.50	NA
2010-2014	32 (33)	17 764 (26.2)	14.1 (8.9-19.4)	.85	.59
2000-2009	31 (32)	13 040 (19.2)	15.2 (8.3-22.0)	.67	.49
<2000	8 (8)	2500 (3.7)	20.4 (12.7-28.0)	.13	.07
Sampling method					
Random^b^	35 (37)	24 926 (36.7)	17.4 (14.3-20.4)	.06	NA
Convenience	17 (18)	24 334 (35.9)	7.1 (4.9-9.2)	.001^a^	<.001^a^
Survey	4 (4)	4292 (6.3)	2.3 (0-6.0)	.009^a^	<.001^a^
Combination (random, convenience, and survey)^d^	4 (4)	871 (1.3)	47.4 (30.0-64.8)	.03^a^	.002^a^
Not specified	36 (38)	13 416 (19.8)	18.7 (18.7-18.7)	.09	.71
Purchasing method					
Mystery client^b^	40 (42)	23 754 (35)	14.0 (9.0-19.0)	.89	NA
Overt	11 (12)	5252 (7.7)	12.8 (3.2-22.5)	.88	.84
Mystery client and overt	11 (12)	13 435 (19.8)	19.0 (13.3-24.6)	.10	.20
Not specified	34 (35)	25 398 (37.4)	10.1 (6.8-13.4)	.04^a^	.21
Chemical analysis					
Chemical analysis	95 (99)	67 779 (99.9)	13.6 (11.0-16.3)	>.99	
1 Form of chemical analysis	31 (32)	22 708 (33.5)	12.0 (7.1-16.8)	.50	.14
2 Forms of chemical analysis	15 (16)	28 857 (42.5)	12.8 (8.5-17.2)	.72	.19
≥3 Forms of chemical analysis^b^	49 (51)	16 214 (23.9)	17.3 (12.3-22.3)	.16	NA
Performed chemical and visual analysis	48 (50)	41 747 (61.5)	8.8 (5.8-11.8)	.003^a^	
Chemical testing method^e^					
HPLC	60 (63)	44 177 (65.1)	14.2 (10.8-17.7)	.73	
Dissolution	32 (33)	10 118 (14.9)	12.3 (7.4-17.2)	.59	
Disintegration	15 (16)	5222 (7.7)	16.0 (7.4-24.5)	.60	
GPHF-Minilab	26 (27)	30 712 (45.3)	7.7 (5.5-9.9)	<.001^a^	
TLC	13 (14)	4884 (7.2)	24.8 (14.6-35.0)	.05	
UV spectrometry	26 (27)	8621 (12.7)	19.6 (13.6-25.5)	.06	
Colorimetry	14 (15)	4287 (6.3)	24.0 (11.6-36.3)	.13	
Mass spectrometry	10 (10)	15 239 (22.5)	18.9 (14.0-23.7)	.06	
Other analyses	40 (42)	17 231 (25.4)	14.8 (9.7-20.0)	.66	
Conflict of interest					
No discernable conflict of interest^b^	69 (72)	54 120 (79.8)	14.2 (11.1-17.4)	.71	NA
Potential conflict of interest	12 (13)	8769 (12.9)	9.3 (6.5-12.2)	.01^a^	.03^a^
Unclear	15 (16)	4950 (7.3)	14.4 (4.8-24.0)	.87	.97
MEDQUARG score^f^					
≥6^g^	53 (55)	34 077 (50.2)	15.9 (12.1-19.7)	.25	NA
<6	43 (45)	33 762 (49.8)	10.9 (7.1-14.6)	.16	.07
Total	96 (100)	67 839 (100)	13.6 (11.0-16.3)		*I*^2^ = 99.9%

Abbreviations: GPHF-Minilab, Global Pharma Health Fund Minilab; HPLC, high-performance liquid chromatography; MEDQUARG, Medical Quality Assessment Reporting Guidelines; NA, not applicable; TLC, thin-layer chromatography; UV, ultraviolet and visible spectroscopy.

^a^ *P* < .05.

^b^ Categorical gold standard, as recommended by Newton et al.^16^

^c^ Number of studies in the subcategory was too small to be used as the categorical gold standard.

^d^ Total samples for subcategorization was less than 1000; thus, results lack statistical rigor.

^e^ As some studies were in more than 1 category, further subcategories could not be compared.

^f^ Reported prevalence was not weighted by the MEDQUARG score.

^g^ Studies with a MEDQUARG score of 6 or greater were considered to have been reported with sufficient quality.

To further examine the association between methodological quality of the studies and the reported SF prevalence, we statistically compared the mean prevalence of SF medicines for each of the 8 subcategorizations with the gold standard for each study characteristic. Small sample sizes and less rigorous sampling methods resulted in significant differences from the categorical gold standard of larger sample sizes (7.0%) and randomized sampling (17.4%). The mean prevalence of studies with potential conflict of interest (9.3%) was also significantly lower than both the overall prevalence (13.6%; *P* = .01) and studies without discernable conflict of interest (14.2%; *P* = .03). Interestingly, the mean SF prevalence of studies with adequate reporting quality (MEDQUARG score ≥6; 15.9%) was 5% higher than studies with a lower quality score (10.9%) but not significantly so (*P* = .07).

A random-effects model was used to examine studies for heterogeneity and publication bias. Studies in the meta-analysis indicate large amounts of heterogeneity (*I*^2^ = 99.9%), and the regression test for funnel plot asymmetry indicates publication bias (*P* < .001). A mixed-effects model was used to test for 8 different potential modifiers where sample size was found to be the only significant modifier (*P* = .04) (eAppendix 3 in the Supplement).

### Economic Evidence and Estimated Impact

Our search for economic impact estimates yielded 65 unique records with each citing 1 or more of 17 different estimates of the economic effect of SF medicines. Three of these estimates were found to be citation errors, 4 did not estimate total economic impact or market size, and the root sources of 2 were untraceable, resulting in 8 economic estimates.

eTable 3 in the Supplement presents the original sources of the 8 economic estimates, primarily of market size, ranging from $10 billion to $200 billion annually (median, $31.25 billion). Of the 8 estimates traced back to their origin,^5,128,129,130,131,132,133,134^ many were old and based on crude calculations by authors without methodological disclosure. Four estimates came from reports from international or intergovernmental organizations but 3 did not note how the estimates were derived. Three estimates were introduced as a rough back-of-the-envelope calculation in a peer-reviewed journal and 2 sources were potentially partisan involving a moderator’s guide from a think tank and a book.^129,134^

## Discussion

Findings from this systematic review and meta-analysis reveal that 13.6% of essential medicines tested in LMICs failed quality analysis. The highest prevalence of poor-quality medicines was observed in Africa, where 18.7% of samples were substandard or falsified. Deficiencies in quality were more prevalent among antimalarials (19.1%) than antibiotics (12.4%), while further studies are needed to understand the quality profile of other essential medicines.

These findings are similar to the estimated prevalence from a 2017 report by the WHO^5^ and consistent with ranges reported in other reviews.^3,20,21,22,23,24,25,26,27,28,29^ All studies within these reviews that met our inclusion criteria were included in our review; the slight variance between our results and those of previous analyses is likely due to these reviews including studies with small sample sizes as well as the sheer amount of study heterogeneity.

This review identified a significant amount of study heterogeneity and potential issues of quality of the prevalence data reported in publications. This is a significant issue as poor-quality prevalence data affect analyses of the health and economic effects of SF medicines as well as crucial policy and regulatory efforts to address the issue.^6^ Unfortunately, controversy over the role of industry in defining the problem of SF medicines has slowed global efforts to address this issue in recent decades.^6,14^ The International Medical Products Anti-counterfeiting Taskforce (IMPACT), established by the WHO in 2006, disbanded over perceived industry connections, and intellectual property concerns waylaid the agreement on the definition of SF medical products.^135^ Concerns that anticounterfeiting efforts could adversely affect the legitimate generic drug industry—essential for access to medicines in LMICs—have been central to the debate.^136^ This dispute has distracted from the public health and socioeconomic consequences as well as efforts to accurately assess the scope of the issue. As a result, efforts have been disjointed, and numerous studies with small sample sizes that do not use rigorous randomization, collection, and analysis methods have been conducted.

This review also identifies a significant gap in the literature on the economic burden of SF medicines due to the poor-quality of reported economic estimates and limited focus, primarily on market size. Robust economic analysis capturing the broader economic burden of SF medicines, such as additional costs of treatment and productivity losses, is critical to understanding the extent of the problem, raising awareness, developing intervention strategies, and fostering change. Future economic research is important to inform efforts to combat the falsification of medications and should be conducted following rigorous economic methods.^137,138^

As demonstrated in the results of our prevalence subgroup analysis, use of less rigorous research, analysis, and reporting methods is detrimental to efforts to assess the scope of the issue because of the number of biases they introduce.^5,16,139^ Studies aiming to support policy development should therefore follow rigorous standards of sampling, analysis, reporting, and disclosure. While metrics such as MEDQUARG exist to guide in the reporting of studies of the prevalence of SF medicines, greater effort and emphasis need to be placed on researching and standardizing international sampling, collection, and analysis protocols.^5,16,139^ Improving quality control and laboratory capacity in LMICs is also crucial as the GPHF-Minilab that is used in these settings in lieu of full pharmacopeial analysis has poor sensitivity to detect substandard medicines.^11,67,123,140^ Furthermore, to ensure that these efforts bear fruit, greater transparency is needed in the disclosure of industry-related potential conflicts of interest. This heterogeneity in findings serves to caution against extrapolating SF prevalences to other regions and across medicine categories.

Countries with weak pharmaceutical governance and poor pharmacovigilance are at the greatest risk from SF medications.^6,141,142^ Weak regulatory capacity to license manufacturers, ensure good manufacturing practices, and perform quality control encourages the illicit manufacture and distribution of SF medications.^142^ Poor supply chain management and surveillance not only open the door to allow SF medicines to permeate the supply chain, but also cause stock-outs that drive patients to purchase medicines from unregulated markets.^142^ Therefore, efforts to improve supply-chain management, surveillance, and regulatory capacity in LMICs are essential to reduce the threat of SF medicines. The successes of the Promoting the Quality of Medicines and the WHO prequalified drug programs demonstrate that these efforts can reduce the prevalence of SF medicines.^137^

While the effects of SF medications disproportionately rest on LMICs, SF medicines originate from and are reported in every country worldwide.^6,142^ The global nature of the medicine supply chain implies that weaknesses in any country in the supply chain affect all the countries downstream.^142,143,144^ This threatens global health security by increasing transmission, morbidity, mortality, and antimicrobial resistance, highlighting the need for a unified global effort to address the issue.^142,143,144^ In 2013, the WHO Global Surveillance and Monitoring System was launched to gather data, improve reporting, and strengthen regulatory capacity globally.^6^ While this is an important first step, additional efforts to implement laws on drug quality and improve data sharing among stakeholders are essential to complement research and technical initiatives.^144^ Nayyar et al^145^ have called for an international convention on SF medicines, similar to the 1929 treaty that internationally criminalized counterfeit banknotes or the Framework Convention on Tobacco Control.^146,147^ Such a convention could (1) accurately define SF medicines, (2) require signatory countries to enact national laws (and consequent prosecution penalties) criminalizing intentional manufacture, trafficking, or selling of SF medicines, (3) provide a legal and institutional framework on convergent medicine regulation, and (4) provide LMICs with financial and technical assistance to effectively join local and regional SF medicine regulatory networks. Without such concerted global effort, the global supply chain, and thereby every country within it, remains vulnerable to SF medicines.

### Limitations

Systematic reviews are inherently limited by their search strategies, databases searched, and the inclusion and exclusion criteria selected.^148^ To address this limitation, we ran 2 searches, performed a systematic reference review, and examined other pertinent database sources. Furthermore, as with all meta-analyses, ours is limited by the quality of the included studies and any biases they may contain.^148^ As demonstrated by the high amount of heterogeneity, the summary statistics reported in this study reflect a wide range of studies and methods, and as a result are subject to various limitations. For studies involving multiple medicines and countries, only the total sample size was included in the prevalence calculations. It is therefore possible that the regional variation observed is explained by differences in sample size by medicine, or that the variation by medicines is explained by the geographic distribution of samples. Differences in study quality, sampling, or purchasing and collection method across regions and medicine categories may have also introduced bias. To control for these potential sources of bias, we selected studies that tested 50 or more samples and removed studies with very poor sampling methods or no description of study methods. To further ensure the rigor of our reported prevalence estimates, each study prevalence was weighted by sample size and a metric that assessed the quality of the reported studies. Quality analyses indicate that there is significant publication bias as well as a moderating effect of sample size, even after controlling for it, which demonstrates the significance of rigorous sampling methods. Finally, reported economic impact estimates are limited by the poor quality and large heterogeneity of available data. Despite these limitations, we believe we appropriately controlled for bias to the best of our ability and have identified and synthesized articles in a systematic and methodical manner.

## Conclusions

Our findings suggest that SF essential medicines are a substantial and understudied problem in LMICs with high estimated economic impact. Reducing their prevalence is imperative to the Global Health Security Agenda, reaching the United Nations Sustainable Development Goals, and global efforts to curb antimicrobial resistance.^1,149^ Although the literature on prevalence continues to grow, methodological standards are needed to improve generalizability and facilitate comparison across studies. Precise, independent estimates are also needed to describe the health and economic effects of poor-quality medicines to build the evidence base for successful policy interventions to curb SF medicines in LMICs. Efforts to strengthen supply-chain management, surveillance, and regulatory capacity are essential to effectively control SF medicines. Globalization necessitates global coordination across national, regional, and sectoral stakeholders to improve the regulation, standardization, and surveillance of the quality of medicines worldwide.

## Notes

Supplement. **eAppendix 1.** Comprehensive Search Strategy **eReferences 1.** Systematic Review Studies Not Included in Meta-analysis **eAppendix 2.** MEDQUARG Scores and Interrater Reliability **eTable 1.** MEDQUARG Scores of Studies Included in the Meta-analysis **eAppendix 3.** Meta-analysis Bias and Quality Analysis **eFigure 1. **Funnel Plot **eFigure 2.** Baujat Plot **eFigure 3. **Influence Plot and Output **eTable 2.** Descriptive Statistics of All Studies in Systematic Review **eFigure 4.** Reported Prevalence of Substandard and Falsified Drugs in Studies in Africa **eFigure 5.** Reported Prevalence of Substandard and Falsified Drugs in Studies in Asia **eFigure 6.** Reported Prevalence of Substandard and Falsified Drugs in Studies that Examined Multiple Regions **eFigure 7.** Reported Prevalence of Substandard and Falsified Drugs in Studies in Other Regions (South America, Europe, and Oceania) **eTable 3.** Estimates of the Economic Impact of Substandard and Falsified Medicines **eReferences 2.** eAppendix References
