# Supplementary material for: Prevalence and Estimated Economic Burden of Substandard and Falsified Medicines in Low- and Middle-Income Countries: A Systematic Review and Meta-analysis
Source: JAMA Netw Open. 2018 Aug 10;1(4):e181662. doi: 10.1001/jamanetworkopen.2018.1662 (PMC6324280; doi:10.1001/jamanetworkopen.2018.1662)
Supplement: Supplement. — eAppendix 1. Comprehensive Search Strategy eReferences 1. Systematic Review Studies Not Included in Meta-analysis eAppendix 2. MEDQUARG Scores and Interrater Reliability eTable 1. MEDQUARG Scores of Studies Included in the Meta-analysis eAppendix 3. Meta-analysis Bias and Quality Analysis eFigure 1. Funnel Plot eFigure 2. Baujat Plot eFigure 3. Influence Plot and Output eTable 2. Descriptive Statistics of All Studies in Systematic Review eFigure 4. Reported Prevalence of Substandard and Falsified Drugs in Studies in Africa eFigure 5. Reported Prevalence of Substandard and Falsified Drugs in Studies in Asia eFigure 6. Reported Prevalence of Substandard and Falsified Drugs in Studies that Examined Multiple Regions eFigure 7. Reported Prevalence of Substandard and Falsified Drugs in Studies in Other Regions (South America, Europe, and Oceania) eTable 3. Estimates of the Economic Impact of Substandard and Falsified Medicines eReferences 2. eAppendix References [file jamanetwopen-1-e181662-s001.pdf]

Supplementary Online Content

Ozawa S, Evans DR, Bessias S, et al. Prevalence and Estimated Economic Burden of Substandard and Falsified Medicines in Low- and Middle-Income Countries: A Systematic Review and Meta-analysis. *JAMA Netw Open*. 2018;1(4):e181662. doi:10.1001/jamanetworkopen.2018.1662

eAppendix 1. Comprehensive Search Strategy

eReferences 1. Systematic Review Studies Not Included in Meta-analysis

eAppendix 2. MEDQUARG Scores and Interrater Reliability

eTable 1. MEDQUARG Scores of Studies Included in the Meta-analysis

eAppendix 3. Meta-analysis Bias and Quality Analysis

eFigure 1. Funnel Plot

eFigure 2. Baujat Plot

eFigure 3. Influence Plot and Output

eTable 2. Descriptive Statistics of All Studies in Systematic Review

eFigure 4. Reported Prevalence of Substandard and Falsified Drugs in Studies in Africa

eFigure 5. Reported Prevalence of Substandard and Falsified Drugs in Studies in Asia

eFigure 6. Reported Prevalence of Substandard and Falsified Drugs in Studies that Examined Multiple Regions

eFigure 7. Reported Prevalence of Substandard and Falsified Drugs in Studies in Other Regions (South America, Europe, and Oceania)

eTable 3. Estimates of the Economic Impact of Substandard and Falsified Medicines

eReferences 2. eAppendix References

This supplementary material has been provided by the authors to give readers additional information about their work.

© 2018 Ozawa S et al. *JAMA Network Open.*

© 2018 Ozawa S et al. *JAMA Network Open.*

eAppendix 1: Comprehensive Search Strategy

Total before duplicates removed: 4284
Total after duplicates removed: 3164

First Search

Search for studies examining the prevalence of substandard and falsified medicines in LMICs

Conducted: January 12th, 2017; Updated: November 3rd, 2017
Total before duplicates removed: 2510
Total after duplicates removed: 1839

PubMed

Search	Query	Items Found
#1	("Counterfeit Drugs"[Mesh] OR ((counterfeit[tw] OR counterfeited[tw] OR counterfeits[tw] OR fake[tw] OR fakes[tw] OR faked[tw] OR falsified[tw] OR false[tw] OR counterfeiting[tw] OR substandard[tw] OR spurious[tw]) AND (medicine[tw] OR medicines[tw] OR medication[tw] OR medications[tw] OR drug[tw] OR drugs[tw] OR pharmaceutical[tw] OR pharmaceuticals[tw] OR "Pharmaceutical Preparations"[Mesh])))	17444
#2	("Counterfeit Drugs"[Mesh] OR ((counterfeit[tw] OR counterfeited[tw] OR counterfeits[tw] OR fake[tw] OR fakes[tw] OR faked[tw] OR falsified[tw] OR false[tw] OR counterfeiting[tw] OR substandard[tw] OR spurious[tw]) AND (medicine[tw] OR medicines[tw] OR medication[tw] OR medications[tw] OR drug[tw] OR drugs[tw] OR pharmaceutical[tw] OR pharmaceuticals[tw] OR "Pharmaceutical Preparations"[Mesh]))) NOT ("false positive"[tw] OR "false negative"[tw] OR "false transmitter"[tw] OR "false transmitters"[tw] OR "false result"[tw] OR "false results"[tw])	8064
#3	((Deprived Countries[tw] OR Deprived Population[tw] OR Deprived Populations[tw] OR Developing Countries[tw] OR Developing Country[tw] OR Developing Economies[tw] OR Developing Economy[tw] OR Developing Nation[tw] OR Developing Nations[tw] OR Developing Population[tw] OR Developing Populations[tw] OR Developing World[tw] OR LAMI Countries[tw] OR LAMI Country[tw] OR Less Developed Countries[tw] OR Less Developed Country[tw] OR Less Developed Economies [tw] OR Less Developed Nation[tw] OR Less Developed Nations[tw] OR Less Developed World[tw] OR Lesser Developed Countries[tw] OR Lesser Developed Nations[tw] OR LMIC[tw] OR LMICS[tw] OR Low GDP[tw] OR Low GNP[tw] OR Low Gross Domestic[tw] OR Low Gross National[tw] OR Low Income Countries[tw] OR Low Income Country[tw] OR Low Income Economies [tw] OR Low Income Economy[tw] OR Low Income Nations[tw] OR Low Income Population[tw] OR Low Income Populations[tw] OR Lower GDP[tw] OR lower gross domestic[tw] OR Lower Income Countries[tw] OR Lower Income Country[tw] OR Lower Income Nations[tw] OR Lower Income Population[tw] OR Lower Income Populations[tw] OR Middle Income Countries[tw] OR Middle Income Country[tw] OR Middle Income Economies [tw] OR Middle Income Nation[tw] OR Middle Income Nations[tw] OR Middle Income Population[tw] OR Middle Income Populations[tw] OR Poor Countries[tw] OR Poor Country[tw] OR Poor Economies [tw] OR Poor Economy[tw] OR Poor Nation[tw] OR Poor Nations[tw] OR Poor Population[tw] OR	129398

© 2018 Ozawa S et al. *JAMA Network Open.*

	Poor Populations[tw] OR poor world[tw] OR Poorer Countries[tw] OR Poorer Economies [tw] OR Poorer Economy[tw] OR Poorer Nations[tw] OR Poorer Population[tw] OR Poorer Populations[tw] OR Third World[tw] OR Transitional Countries[tw] OR Transitional Country[tw] OR Transitional Economies[tw] OR Transitional Economy[tw] OR Under Developed Countries[tw] OR Under Developed Country[tw] OR under developed nations[tw] OR Under Developed World[tw] OR Under Served Population[tw] OR Under Served Populations[tw] OR Underdeveloped Countries[tw] OR Underdeveloped Country[tw] OR underdeveloped economies[tw] OR underdeveloped nations[tw] OR underdeveloped population[tw] OR Underdeveloped World[tw] OR Underserved Countries[tw] OR Underserved Nations[tw] OR Underserved Population[tw] OR Underserved Populations[tw]))	
#4	(Afghanistan[tw] OR Albania[tw] OR Algeria[tw] OR "American Samoa"[tw] OR Angola[tw] OR Armenia[tw] OR Azerbaijan[tw] OR Bangladesh[tw] OR Belarus[tw] OR Byelarus[tw] OR Belorussia[tw] OR Belize[tw] OR Benin[tw] OR Bhutan[tw] OR Bolivia[tw] OR Bosnia[tw] OR Botswana[tw] OR Brazil[tw] OR Bulgaria[tw] OR Burma[tw] OR "Burkina Faso"[tw] OR Burundi[tw] OR "Cabo Verde"[tw] OR "Cape verde"[tw] OR Cambodia[tw] OR Cameroon[tw] OR "Central African Republic"[tw] OR Chad[tw] OR China[tw] OR Colombia[tw] OR Comoros[tw] OR Comores[tw] OR Comoro[tw] OR Congo[tw] OR "Costa Rica"[tw] OR "Côte d'Ivoire"[tw] OR Cuba[tw] OR Djibouti[tw] OR Dominica[tw] OR "Dominican Republic"[tw] OR Ecuador[tw] OR Egypt[tw] OR "El Salvador"[tw] OR Eritrea[tw] OR Ethiopia[tw] OR Fiji[tw] OR Gabon[tw] OR Gambia[tw] OR Gaza[tw] OR "Georgia Republic"[tw] OR Georgian[tw] OR Ghana[tw] OR Grenada[tw] OR Grenadines[tw] OR Guatemala[tw] OR Guinea[tw] OR "Guinea Bisau"[tw] OR Guyana[tw] OR Haiti[tw] OR Herzegovina[tw] OR Hercegovina[tw] OR Honduras[tw] OR India[tw] OR Indonesia[tw] OR Iran[tw] OR Iraq[tw] OR Jamaica[tw] OR Jordan[tw] OR Kazakhstan[tw] OR Kenya[tw] OR Kiribati[tw] OR Korea[tw] OR Kosovo[tw] OR Kyrgyz[tw] OR OR Kirghizia[tw] OR Kirghiz[tw] OR Kirgizstan[tw] OR Kyrgyzstan[tw] OR "Lao PDR"[tw] OR Laos[tw] OR Lebanon[tw] OR Lesotho[tw] OR Liberia[tw] OR Libya[tw] OR Macedonia[tw] OR Madagascar[tw] OR Malawi[tw] OR Malay[tw] OR Malaya[tw] OR Malaysia[tw] OR Maldives[tw] OR Mali[tw] OR "Marshall Islands"[tw] OR Mauritania[tw] OR Mauritius[tw] OR Mexico[tw] OR Micronesia[tw] OR Moldova[tw] OR Mongolia[tw] OR Montenegro[tw] OR Morocco[tw] OR Mozambique[tw] OR Myanmar[tw] OR Namibia[tw] OR Nepal[tw] OR Nicaragua[tw] OR Niger[tw] OR Nigeria [tw] OR Pakistan [tw] OR Palau[tw] OR Panama[tw] OR "Papua New Guinea"[tw] OR Paraguay[tw] OR Peru [tw] OR Philippines[tw] OR Phillippines[tw] OR Philipines[tw] OR Phillipines[tw] OR Principe[tw] OR Romania[tw] OR Rwanda[tw] OR Ruanda[tw] OR Samoa[tw] OR "Sao Tome"[tw] OR Senegal[tw] OR Serbia[tw] OR "Sierra Leone"[tw] OR "Solomon Islands"[tw] OR Somalia[tw] OR "South Africa"[tw] OR "South Sudan"[tw] OR "Sri Lanka"[tw] OR "St Lucia"[tw] OR "St Vincent"[tw] OR Sudan[tw] OR Suriham[tw] OR Suriname[tw] OR Swaziland[tw] OR Syria[tw] OR "Syrian Arab Republic"[tw] OR Tajikistan[tw] OR Tadzhikistan[tw] OR Tadjikistan[tw] OR Tadzhik[tw] OR Tanzania[tw] OR Thailand[tw] OR Timor[tw] OR Togo[tw] OR Tonga[tw] OR Tunisia[tw] OR Turkey[tw] OR Turkmen[tw] OR Turkmenistan[tw] OR Tuvalu[tw] OR Uganda[tw] OR Ukraine[tw] OR Uzbek[tw] OR Uzbekistan[tw] OR Vanuatu[tw] OR Vietnam[tw] OR "West Bank"[tw] OR Yemen[tw] OR Zambia[tw] OR Zimbabwe[tw]))	1155076
#5	#2 AND (#3 OR #4)	714

© 2018 Ozawa S et al. *JAMA Network Open.*

#6	#5 Rerun November 3rd, 2017 (Publications since first search)	58

PubMed before duplicates removed: 772
PubMed after duplicates removed: 760

Scopus

Search	Query	Items Found
#1	TITLE-ABS-KEY ((counterfeit W/3 medicine) OR (counterfeited W/3 medicine) OR (counterfeits W/3 medicine) OR (fake W/3 medicine) OR (fakes W/3 medicine) OR (faked W/3 medicine) OR (falsified W/3 medicine) OR (false W/3 medicine) OR (counterfeiting W/3 medicine) OR (substandard W/3 medicine) OR (spurious W/3 medicine) OR (counterfeit W/3 medicines) OR (counterfeited W/3 medicines) OR (counterfeits W/3 medicines) OR (fake W/3 medicines) OR (fakes W/3 medicines) OR (faked W/3 medicines) OR (falsified W/3 medicines) OR (false W/3 medicines) OR (counterfeiting W/3 medicines) OR (substandard W/3 medicines) OR (spurious W/3 medicines) OR (counterfeit W/3 medication) OR (counterfeited W/3 medication) OR (counterfeits W/3 medication) OR (fake W/3 medication) OR (fakes W/3 medication) OR (faked W/3 medication) OR (falsified W/3 medication) OR (false W/3 medication) OR (counterfeiting W/3 medication) OR (substandard W/3 medication) OR (spurious W/3 medication) OR (counterfeit W/3 medications) OR (counterfeited W/3 medications) OR (counterfeits W/3 medications) OR (fake W/3 medications) OR (fakes W/3 medications) OR (faked W/3 medications) OR (falsified W/3 medications) OR (false W/3 medications) OR (counterfeiting W/3 medications) OR (substandard W/3 medications) OR (spurious W/3 medications) OR (counterfeit W/3 drug) OR (counterfeited W/3 drug) OR (counterfeits W/3 drug) OR (fake W/3 drug) OR (fakes W/3 drug) OR (faked W/3 drug) OR (falsified W/3 drug) OR (false W/3 drug) OR (counterfeiting W/3 drug) OR (substandard W/3 drug) OR (spurious W/3 drug) OR (counterfeit W/3 drugs) OR (counterfeited W/3 drugs) OR (counterfeits W/3 drugs) OR (fake W/3 drugs) OR (fakes W/3 drugs) OR (faked W/3 drugs) OR (falsified W/3 drugs) OR (false W/3 drugs) OR (counterfeiting W/3 drugs) OR (substandard W/3 drugs) OR (spurious W/3 drugs) OR (counterfeit W/3 pharmaceutical) OR (counterfeited W/3 pharmaceutical) OR (counterfeits W/3 pharmaceutical) OR (fake W/3 pharmaceutical) OR (fakes W/3 pharmaceutical) OR (faked W/3 pharmaceutical) OR (falsified W/3 pharmaceutical) OR (false W/3 pharmaceutical) OR (counterfeiting W/3 pharmaceutical) OR (substandard W/3 pharmaceutical) OR (spurious W/3 pharmaceutical) OR (counterfeit W/3 pharmaceuticals) OR (counterfeited W/3 pharmaceuticals) OR (counterfeits W/3 pharmaceuticals) OR (fake W/3 pharmaceuticals) OR (fakes W/3 pharmaceuticals) OR (faked W/3 pharmaceuticals) OR (falsified W/3 pharmaceuticals) OR (false W/3 pharmaceuticals) OR (counterfeiting W/3 pharmaceuticals) OR (substandard W/3 pharmaceuticals) OR (spurious W/3 pharmaceuticals))	2,251
#2	TITLE-ABS-KEY ("false positive" OR "false negative" OR "false transmitter" OR "false transmitters" OR "false result" OR "false results")	117,213
#3	#1 AND NOT #2	2,011
#4	TITLE-ABS-KEY ("deprived countries" OR "deprived country" OR "deprived	275,059

© 2018 Ozawa S et al. *JAMA Network Open.*

	nation" OR "deprived nations" OR "deprived population" OR "deprived populations" OR "deprived world" OR "developing countries" OR "developing country" OR "developing economies" OR "developing economy" OR "developing nation" OR "developing nations" OR "developing population" OR "developing populations" OR "developing world" OR "lami countries" OR "lami country" OR "less developed countries" OR "less developed country" OR "less developed economies" OR "less developed economy" OR "less developed nation" OR "less developed nations" OR "less developed population" OR "less developed populations" OR "less developed world" OR "lesser developed countries" OR "lesser developed country" OR "lesser developed economies" OR "lesser developed economy" OR "lesser developed nation" OR "lesser developed nations" OR "lesser developed population" OR "lesser developed populations" OR "lesser developed world" OR "LMIC" OR "LMICS" OR "low gdp" OR "low gnp" OR "low gross domestic" OR "low gross national" OR "low income countries" OR "low income country" OR "low income economies" OR "low income economy" OR "low income nation" OR "low income nations" OR "low income population" OR "low income populations" OR "lower gdp" OR "lower gnp" OR "lower gross domestic" OR "lower gross national" OR "lower income countries" OR "lower income country" OR "lower income economies" OR "lower income economy" OR "lower income nation" OR "lower income nations" OR "lower income population" OR "lower income populations" OR "middle income countries" OR "middle income country" OR "middle income economies" OR "middle income economy" OR "middle income nation" OR "middle income nations" OR "middle income population" OR "middle income populations" OR "poor countries" OR "poor country" OR "Poor Economies" OR "Poor Economy" OR "poor nation" OR "poor nations" OR "poor population" OR "poor populations" OR "poor world" OR "poorer countries" OR "poorer country" OR "Poorer Economies" OR "Poorer Economy" OR "poorer nation" OR "poorer nations" OR "poorer population" OR "poorer populations" OR "poorer world" OR "third world" OR "transitional countries" OR "transitional country" OR "Transitional Economies" OR "Transitional Economy" OR "under developed countries" OR "under developed country" OR "under developed economies" OR "under developed economy" OR "under developed nation" OR "under developed nations" OR "under developed population" OR "under developed populations" OR "under developed world" OR "under served countries" OR "under served country" OR "under served nation" OR "under served nations" OR "under served population" OR "under served populations" OR "under served world" OR "underdeveloped countries" OR "underdeveloped country" OR "underdeveloped economies" OR "underdeveloped economy" OR "underdeveloped nation" OR "underdeveloped nations" OR "underdeveloped population" OR "underdeveloped populations" OR "underdeveloped world" OR "underserved countries" OR "underserved country" OR "underserved nation" OR "underserved nations" OR "underserved population" OR "underserved populations" OR "underserved world")	
#5	TITLE-ABS-KEY (afghanistan OR albania OR algeria OR "American Samoa" OR angola OR armenia OR azerbaijan OR bangladesh OR belarus OR byelarus OR belorussia OR belize OR benin OR bhutan OR bolivia OR bosnia OR botswana OR brazil OR bulgaria OR burma OR "Burkina Faso"	3,101,742

© 2018 Ozawa S et al. *JAMA Network Open.*

	OR burundi OR "Cabo Verde" OR "Cape verde" OR cambodia OR cameroon OR "Central African Republic" OR chad OR china OR colombia OR comoros OR comores OR comoro OR congo OR "Costa Rica" OR "Côte d'Ivoire" OR cuba OR djibouti OR dominica OR "Dominican Republic" OR ecuador OR egypt OR "El Salvador" OR eritrea OR ethiopia OR fiji OR gabon OR gambia OR gaza OR "Georgia Republic" OR georgian OR ghana OR grenada OR grenadines OR guatemala OR guinea OR "Guinea Bisau" OR guyana OR haiti OR herzegovina OR hercegovina OR honduras OR india OR indonesia OR iran OR iraq OR jamaica OR jordan OR kazakhstan OR kenya OR kiribati OR korea OR kosovo OR kyrgyz OR kirghizia OR kirghiz OR kirgizstan OR kyrgyzstan OR "Lao PDR" OR laos OR lebanon OR lesotho OR liberia OR libya OR macedonia OR madagascar OR malawi OR malay OR malaya OR malaysia OR maldives OR mali OR "Marshall Islands" OR mauritania OR mauritius OR mexico OR micronesia OR moldova OR mongolia OR montenegro OR morocco OR mozambique OR myanmar OR namibia OR nepal OR nicaragua OR niger OR nigeria OR pakistan OR palau OR panama OR "Papua New Guinea" OR paraguay OR peru OR philippines OR phillippines OR philipines OR phillipines OR principe OR romania OR rwanda OR ruanda OR samoa OR "Sao Tome" OR senegal OR serbia OR "Sierra Leone" OR "Solomon Islands" OR somalia OR "South Africa" OR "South Sudan" OR "Sri Lanka" OR "St Lucia" OR "St Vincent" OR sudan OR suriham OR suriname OR swaziland OR syria OR "Syrian Arab Republic" OR tajikistan OR tadzhikistan OR tadjikistan OR tadzhik OR tanzania OR thailand OR timor OR togo OR tonga OR tunisia OR turkey OR turkmen OR turkmenistan OR tuvalu OR uganda OR ukraine OR uzbek OR uzbekistan OR vanuatu OR vietnam OR "West Bank" OR yemen OR zambia OR zimbabwe)	
#6	#4 OR #5	3,244,856
#7	#3 AND #6	537
#8	#7 Rerun November 3rd, 2017 (Publications since first search)	39

Scopus before duplicates removed: 576
Scopus after duplicates removed: 260

Global Health

Search	Query	Items Found
S1	TI (((counterfeit OR counterfeited OR counterfeits OR fake OR fakes OR faked OR falsified OR false OR counterfeiting OR substandard OR spurious) N3 (medicine OR medicines OR medication OR medications OR drug OR drugs OR pharmaceutical OR pharmaceuticals)))) OR AB (((counterfeit OR counterfeited OR counterfeits OR fake OR fakes OR faked OR falsified OR false OR counterfeiting OR substandard OR spurious) N3 (medicine OR medicines OR medication OR medications OR drug OR drugs OR pharmaceutical OR pharmaceuticals)))) OR SU (((counterfeit OR counterfeited OR counterfeits OR fake OR fakes OR faked OR falsified OR false OR counterfeiting OR substandard OR spurious) N3 (medicine OR medicines OR medication OR medications OR	245

© 2018 Ozawa S et al. *JAMA Network Open.*

	drug OR drugs OR pharmaceutical OR pharmaceuticals))))	
S2	TI (("false positive" OR "false negative" OR "false transmitter" OR "false transmitters" OR "false result" OR "false results")) OR AB (("false positive" OR "false negative" OR "false transmitter" OR "false transmitters" OR "false result" OR "false results")) OR SU (("false positive" OR "false negative" OR "false transmitter" OR "false transmitters" OR "false result" OR "false results"))	9,088
S3	S1 NOT S2	231

Global Health before duplicates removed: 231
Global Health after duplicates removed: 115

EconLit

Search	Query	Items Found
S1	TI (((counterfeit OR counterfeited OR counterfeits OR fake OR fakes OR faked OR falsified OR false OR counterfeiting OR substandard OR spurious) N3 (medicine OR medicines OR medication OR medications OR drug OR drugs OR pharmaceutical OR pharmaceuticals)))) OR AB (((counterfeit OR counterfeited OR counterfeits OR fake OR fakes OR faked OR falsified OR false OR counterfeiting OR substandard OR spurious) N3 (medicine OR medicines OR medication OR medications OR drug OR drugs OR pharmaceutical OR pharmaceuticals)))) OR SU (((counterfeit OR counterfeited OR counterfeits OR fake OR fakes OR faked OR falsified OR false OR counterfeiting OR substandard OR spurious) N3 (medicine OR medicines OR medication OR medications OR drug OR drugs OR pharmaceutical OR pharmaceuticals))))	37
S2	TI (("false positive" OR "false negative" OR "false transmitter" OR "false transmitters" OR "false result" OR "false results")) OR AB (("false positive" OR "false negative" OR "false transmitter" OR "false transmitters" OR "false result" OR "false results")) OR SU (("false positive" OR "false negative" OR "false transmitter" OR "false transmitters" OR "false result" OR "false results"))	69
S3	S1 NOT S2	36

EconLit before duplicates removed: 36
EconLit after duplicates removed: 24

Embase

Search	Query	Items Found
#1	'counterfeit drug'/exp OR (counterfeit:ab,ti OR counterfeited:ab,ti OR counterfeits:ab,ti OR fake:ab,ti OR fakes:ab,ti OR faked:ab,ti OR falsified:ab,ti OR false:ab,ti OR counterfeiting:ab,ti OR substandard:ab,ti OR spurious:ab,ti AND (medicine:ab,ti OR medicines:ab,ti OR medication:ab,ti OR medications:ab,ti OR drug:ab,ti OR drugs:ab,ti OR pharmaceutical:ab,ti OR pharmaceuticals:ab,ti OR 'chemicals and drugs'/exp))	12,782
#2	'false positive' OR 'false negative' OR 'false transmitter' OR 'false transmitters' OR 'false result' OR 'false results'	83,351
#3	#1 NOT #2	7,077
#4	'deprived countries' OR 'deprived country' OR 'deprived nation' OR 'deprived	145,063

© 2018 Ozawa S et al. *JAMA Network Open.*

	nations' OR 'deprived population' OR 'deprived populations' OR 'deprived world' OR 'developing countries' OR 'developing country' OR 'developing economies' OR 'developing economy' OR 'developing nation' OR 'developing nations' OR 'developing population' OR 'developing populations' OR 'developing world' OR 'lami countries' OR 'lami country' OR 'less developed countries' OR 'less developed country' OR 'less developed economies' OR 'less developed economy' OR 'less developed nation' OR 'less developed nations' OR 'less developed population' OR 'less developed populations' OR 'less developed world' OR 'lesser developed countries' OR 'lesser developed country' OR 'lesser developed economies' OR 'lesser developed economy' OR 'lesser developed nation' OR 'lesser developed nations' OR 'lesser developed population' OR 'lesser developed populations' OR 'lesser developed world' OR 'lmic' OR 'lmics' OR 'low gdp' OR 'low gnp' OR 'low gross domestic' OR 'low gross national' OR 'low income countries' OR 'low income country' OR 'low income economies' OR 'low income economy' OR 'low income nation' OR 'low income nations' OR 'low income population' OR 'low income populations' OR 'lower gdp' OR 'lower gnp' OR 'lower gross domestic' OR 'lower gross national' OR 'lower income countries' OR 'lower income country' OR 'lower income economies' OR 'lower income economy' OR 'lower income nation' OR 'lower income nations' OR 'lower income population' OR 'lower income populations' OR 'middle income countries' OR 'middle income country' OR 'middle income economies' OR 'middle income economy' OR 'middle income nation' OR 'middle income nations' OR 'middle income population' OR 'middle income populations' OR 'poor countries' OR 'poor country' OR 'poor economies' OR 'poor economy' OR 'poor nation' OR 'poor nations' OR 'poor population' OR 'poor populations' OR 'poor world' OR 'poorer countries' OR 'poorer country' OR 'poorer economies' OR 'poorer economy' OR 'poorer nation' OR 'poorer nations' OR 'poorer population' OR 'poorer populations' OR 'poorer world' OR 'third world' OR 'transitional countries' OR 'transitional country' OR 'transitional economies' OR 'transitional economy' OR 'under developed countries' OR 'under developed country' OR 'under developed economies' OR 'under developed economy' OR 'under developed nation' OR 'under developed nations' OR 'under developed population' OR 'under developed populations' OR 'under developed world' OR 'under served countries' OR 'under served country' OR 'under served nation' OR 'under served nations' OR 'under served population' OR 'under served populations' OR 'under served world' OR 'underdeveloped countries' OR 'underdeveloped country' OR 'underdeveloped economies' OR 'underdeveloped economy' OR 'underdeveloped nation' OR 'underdeveloped nations' OR 'underdeveloped population' OR 'underdeveloped populations' OR 'underdeveloped world' OR 'underserved countries' OR 'underserved country' OR 'underserved nation' OR 'underserved nations' OR 'underserved population' OR 'underserved populations' OR 'underserved world'	
#5	afghanistan OR albania OR algeria OR 'american samoa' OR angola OR armenia OR azerbaijan OR bangladesh OR belarus OR byelarus OR belorussia OR belize OR benin OR bhutan OR bolivia OR bosnia OR botswana OR brazil OR bulgaria OR burma OR 'burkina faso' OR burundi OR 'cabo verde' OR 'cape verde' OR cambodia OR cameroon OR 'central african republic' OR chad OR china OR colombia OR comoros OR comores OR comoro OR congo OR 'costa rica' OR 'cote d ivoire' OR cuba OR djibouti OR dominica OR 'dominican republic' OR ecuador OR egypt OR 'el salvador' OR eritrea OR ethiopia OR fiji OR gabon OR gambia OR	5,201,776

© 2018 Ozawa S et al. *JAMA Network Open.*

	gaza OR 'georgia republic' OR georgian OR ghana OR grenada OR grenadines OR guatemala OR guinea OR 'guinea bisau' OR guyana OR haiti OR herzegovina OR hercegovina OR honduras OR india OR indonesia OR iran OR iraq OR jamaica OR jordan OR kazakhstan OR kenya OR kiribati OR korea OR kosovo OR kyrgyz OR kirghizia OR kirghiz OR kirgizstan OR kyrgyzstan OR 'lao pdr' OR laos OR lebanon OR lesotho OR liberia OR libya OR macedonia OR madagascar OR malawi OR malay OR malaya OR malaysia OR maldives OR mali OR 'marshall islands' OR mauritania OR mauritius OR mexico OR micronesia OR moldova OR mongolia OR montenegro OR morocco OR mozambique OR myanmar OR namibia OR nepal OR nicaragua OR niger OR nigeria OR pakistan OR palau OR panama OR 'papua new guinea' OR paraguay OR peru OR philippines OR phillippines OR philipines OR phillipines OR principe OR romania OR rwanda OR ruanda OR samoa OR 'sao tome' OR senegal OR serbia OR 'sierra leone' OR 'solomon islands' OR somalia OR 'south africa' OR 'south sudan' OR 'sri lanka' OR 'st lucia' OR 'st vincent' OR sudan OR suriham OR suriname OR swaziland OR syria OR 'syrian arab republic' OR tajikistan OR tadzhikistan OR tadjikistan OR tadzhik OR tanzania OR thailand OR timor OR togo OR tonga OR tunisia OR turkey OR turkmen OR turkmenistan OR tuvalu OR uganda OR ukraine OR uzbek OR uzbekistan OR vanuatu OR vietnam OR 'west bank' OR yemen OR zambia OR zimbabwe	
#6	#3 AND (#4 OR #5)	1,723
#7	#6 AND [embase]/lim NOT [medline]/lim	895

Embase before duplicates removed: 895
Embase after duplicates removed: 680

Second Search

Search for studies examining the quality of medicines in LMICs

Conducted: November 3rd, 2017
Total before duplicates removed: 1582
Total after duplicates removed: 1133

PubMed

#1	(quality[ti] AND (medicine[tiab] OR medicines[tiab] OR medication[tiab] OR medications[tiab] OR drug[tiab] OR drugs[tiab] OR pharmaceutical[tiab] OR pharmaceuticals[tiab] OR "Pharmaceutical Preparations "[Mesh]))	17945
#2	“Controlled Substances”[Mesh] OR “Cosmeceuticals”[Mesh] OR “Designer Drugs”[Mesh] OR “Drugs, Investigational”[Mesh] OR “Medical Marijuana”[Mesh] OR “Nostrums”[Mesh] OR “Pharmaceutic Aids”[Mesh] OR “Pharmaceutical Preparations, Dental”[Mesh] OR “Placebos”[Mesh] OR “Plant Extracts”[Mesh] OR “Solutions”[Mesh] OR “Street Drugs”[Mesh] OR “Veterinary Drugs”[Mesh] OR “Materia Medica”[Mesh] OR “Drugs, Chinese Herbal”[Mesh]	323907
#3	(((“quality of life”[tiab] OR “quality of reporting”[tiab] OR “quality of care”[tiab] OR “quality care”[tiab] OR “care quality”[tiab] OR “quality of service”[tiab] OR “quality of services”[tiab] OR “service quality”[tiab] OR “quality of sleep” OR “sleep quality” OR adherence[tiab] OR “diet quality” OR “quality of diet” OR “quality-adjusted”[tw] OR “quality of evidence”[tiab] OR “healthcare quality”[tiab] OR “health care quality”[tiab] OR “quality of health care”[tiab] OR “quality of healthcare”[tiab] OR “quality of	7684052

© 2018 Ozawa S et al. *JAMA Network Open.*

	medical"[tiab] OR "quality health"[tiab] OR "quality of health"[tiab] OR "institute for quality"[tiab] OR "sperm" OR "documentation quality"[tiab] OR "bone quality" OR "solvent quality"[tiab] OR "DNA"[tiab] OR "quality of study"[tiab] OR "quality of studies"[tiab] OR milk OR protein[tiab] OR proteins[tiab] OR "quality innovation"[tiab] OR "quality attribute"[tiab] OR "quality attributes" OR "education"[ti] OR "teaching"[ti] OR instruction[ti] OR "quality constant" OR "genetic"[tiab] OR "gene"[tiab] OR "block"[tiab] OR "quality management"[tiab] OR "management quality"[tiab] OR "treatment quality"[tiab] OR "quality of outcome"[tiab] OR "quality research"[tiab] OR "quality of data"[tiab] OR "data quality"[tiab] OR "environmental quality"[tiab] OR "stem cell" OR intoxication OR sanitation[tiab] OR "herbal"[tiab] OR "traditional"[tiab] OR "retention" OR "ventilation" OR "curriculum" OR "environment"[tiab] OR "physical activity" OR pollution[tiab] OR "drug abuse" OR alcohol OR "substance abuse" OR insecticide[ti] OR "smoking" OR "adverse events" OR acupuncture OR transfusion OR attitudes OR "electronic"[tiab] OR "adverse events"[tw] OR "quality criteria"[ti] OR "clinical trials"[ti] OR environment[ti])))	
#4	("false positive"[tw] OR "false negative"[tw] OR "false transmitter"[tw] OR "false transmitters"[tw] OR "false result"[tw] OR "false results"[tw])	79097
#5	(Afghanistan[tw] OR Albania[tw] OR Algeria[tw] OR "American Samoa"[tw] OR Angola[tw] OR Armenia[tw] OR Azerbaijan[tw] OR Bangladesh[tw] OR Belarus[tw] OR Byelarus[tw] OR Belorussia[tw] OR Belize[tw] OR Benin[tw] OR Bhutan[tw] OR Bolivia[tw] OR Bosnia[tw] OR Botswana[tw] OR Brazil[tw] OR Bulgaria[tw] OR Burma[tw] OR "Burkina Faso"[tw] OR Burundi[tw] OR "Cabo Verde"[tw] OR "Cape verde"[tw] OR Cambodia[tw] OR Cameroon[tw] OR "Central African Republic"[tw] OR Chad[tw] OR China[tw] OR Colombia[tw] OR Comoros[tw] OR Comores[tw] OR Comoro[tw] OR Congo[tw] OR "Costa Rica"[tw] OR "Côte d'Ivoire"[tw] OR Cuba[tw] OR Djibouti[tw] OR Dominica[tw] OR "Dominican Republic"[tw] OR Ecuador[tw] OR Egypt[tw] OR "El Salvador"[tw] OR Eritrea[tw] OR Ethiopia[tw] OR Fiji[tw] OR Gabon[tw] OR Gambia[tw] OR Gaza[tw] OR "Georgia Republic"[tw] OR Georgian[tw] OR Ghana[tw] OR Grenada[tw] OR Grenadines[tw] OR Guatemala[tw] OR Guinea[tw] OR "Guinea Bisau"[tw] OR Guyana[tw] OR Haiti[tw] OR Herzegovina[tw] OR Hercegovina[tw] OR Honduras[tw] OR India[tw] OR Indonesia[tw] OR Iran[tw] OR Iraq[tw] OR Jamaica[tw] OR Jordan[tw] OR Kazakhstan[tw] OR Kenya[tw] OR Kiribati[tw] OR Korea[tw] OR Kosovo[tw] OR Kyrgyz[tw] OR OR Kirghizia[tw] OR Kirghiz[tw] OR Kirgizstan[tw] OR Kyrgyzstan[tw] OR "Lao PDR"[tw] OR Laos[tw] OR Lebanon[tw] OR Lesotho[tw] OR Liberia[tw] OR Libya[tw] OR Macedonia[tw] OR Madagascar[tw] OR Malawi[tw] OR Malay[tw] OR Malaya[tw] OR Malaysia[tw] OR Maldives[tw] OR Mali[tw] OR "Marshall Islands"[tw] OR Mauritania[tw] OR Mauritius[tw] OR Mexico[tw] OR Micronesia[tw] OR Moldova[tw] OR Mongolia[tw] OR Montenegro[tw] OR Morocco[tw] OR Mozambique[tw] OR Myanmar[tw] OR Namibia[tw] OR Nepal[tw] OR Nicaragua[tw] OR Niger[tw] OR Nigeria [tw] OR Pakistan [tw] OR Palau[tw] OR Panama[tw] OR "Papua New Guinea"[tw] OR Paraguay[tw] OR Peru [tw] OR Philippines[tw] OR Phillippines[tw] OR Philipines[tw] OR Phillipines[tw] OR Principe[tw] OR Romania[tw] OR Rwanda[tw] OR Ruanda[tw] OR Samoa[tw] OR "Sao Tome"[tw] OR Senegal[tw] OR Serbia[tw] OR "Sierra Leone"[tw] OR "Solomon Islands"[tw] OR Somalia[tw] OR "South Africa"[tw] OR "South Sudan"[tw] OR "Sri Lanka"[tw] OR "St Lucia"[tw] OR "St Vincent"[tw] OR Sudan[tw] OR Suriham[tw] OR Suriname[tw] OR Swaziland[tw] OR Syria[tw] OR "Syrian Arab Republic"[tw] OR Tajikistan[tw] OR Tadzhikistan[tw] OR Tadjikistan[tw]	1225922

© 2018 Ozawa S et al. *JAMA Network Open.*

	OR Tadzhik[tw] OR Tanzania[tw] OR Thailand[tw] OR Timor[tw] OR Togo[tw] OR Tonga[tw] OR Tunisia[tw] OR Turkey[tw] OR Turkmen[tw] OR Turkmenistan[tw] OR Tuvalu[tw] OR Uganda[tw] OR Ukraine[tw] OR Uzbek[tw] OR Uzbekistan[tw] OR Vanuatu[tw] OR Vietnam[tw] OR "West Bank"[tw] OR Yemen[tw] OR Zambia[tw] OR Zimbabwe[tw]))	
#6	((Deprived Countries[tw] OR Deprived Population[tw] OR Deprived Populations[tw] OR Developing Countries[tw] OR Developing Country[tw] OR Developing Economies[tw] OR Developing Economy[tw] OR Developing Nation[tw] OR Developing Nations[tw] OR Developing Population[tw] OR Developing Populations[tw] OR Developing World[tw] OR LAMI Countries[tw] OR LAMI Country[tw] OR Less Developed Countries[tw] OR Less Developed Country[tw] OR Less Developed Economies [tw] OR Less Developed Nation[tw] OR Less Developed Nations[tw] OR Less Developed World[tw] OR Lesser Developed Countries[tw] OR Lesser Developed Nations[tw] OR LMIC[tw] OR LMICS[tw] OR Low GDP[tw] OR Low GNP[tw] OR Low Gross Domestic[tw] OR Low Gross National[tw] OR Low Income Countries[tw] OR Low Income Country[tw] OR Low Income Economies [tw] OR Low Income Economy[tw] OR Low Income Nations[tw] OR Low Income Population[tw] OR Low Income Populations[tw] OR Lower GDP[tw] OR lower gross domestic[tw] OR Lower Income Countries[tw] OR Lower Income Country[tw] OR Lower Income Nations[tw] OR Lower Income Population[tw] OR Lower Income Populations[tw] OR Middle Income Countries[tw] OR Middle Income Country[tw] OR Middle Income Economies [tw] OR Middle Income Nation[tw] OR Middle Income Nations[tw] OR Middle Income Population[tw] OR Middle Income Populations[tw] OR Poor Countries[tw] OR Poor Country[tw] OR Poor Economies [tw] OR Poor Economy[tw] OR Poor Nation[tw] OR Poor Nations[tw] OR Poor Population[tw] OR Poor Populations[tw] OR poor world[tw] OR Poorer Countries[tw] OR Poorer Economies [tw] OR Poorer Economy[tw] OR Poorer Nations[tw] OR Poorer Population[tw] OR Poorer Populations[tw] OR Third World[tw] OR Transitional Countries[tw] OR Transitional Country[tw] OR Transitional Economies[tw] OR Transitional Economy[tw] OR Under Developed Countries[tw] OR Under Developed Country[tw] OR under developed nations[tw] OR Under Developed World[tw] OR Under Served Population[tw] OR Under Served Populations[tw] OR Underdeveloped Countries[tw] OR Underdeveloped Country[tw] OR underdeveloped economies[tw] OR underdeveloped nations[tw] OR underdeveloped population[tw] OR Underdeveloped World[tw] OR Underserved Countries[tw] OR Underserved Nations[tw] OR Underserved Population[tw] OR Underserved Populations[tw]))	136241
#7	#1 NOT (#2 OR #3 OR #4)	5155
#8	#7 AND (#5 OR #6)	452

PubMed before duplicates removed: 452
PubMed after duplicates removed: 388

Scopus

#1	TITLE ((quality W/3 medicine) OR (quality W/3 medicines) OR (quality W/3 medication) OR (quality W/3 medications) OR (quality W/3 drug) OR (quality W/3 tablets) OR (quality W/3 pills) OR (quality W/3 drugs) OR (quality W/3 tablet) OR (quality W/3 pill) OR (quality W/3 pharmaceutical) OR (quality W/3 pharmaceuticals))	3706

© 2018 Ozawa S et al. *JAMA Network Open.*

#2	TITLE ("quality of life" OR "quality of reporting" OR "quality of care" OR "quality care" OR "care quality" OR "quality of service" OR "quality of services" OR "service quality" OR "quality of sleep" OR "sleep quality" OR “adherence” OR "diet quality" OR "quality of diet" OR "quality-adjusted" OR "quality of evidence" OR "healthcare quality" OR "health care quality" OR "quality of health care" OR "quality of healthcare" OR "quality of medical" OR "quality health" OR "quality of health" OR "institute for quality" OR "documentation quality" OR "bone quality" OR "solvent quality" OR "quality of study" OR "quality of studies" OR “milk” OR “protein” OR “proteins” OR "quality innovation" OR "quality attribute" OR "quality attributes" OR "quality education" OR "quality educational" OR "education quality" OR "quality of teaching" OR "teaching quality" OR "quality of instruction" OR "quality constant" OR "quality management" OR "management quality" OR "treatment quality" OR "quality of outcome" OR "quality research" OR "quality of data" OR "data quality" OR “air quality” OR "environment" OR "retention" OR "ventilation" OR "curriculum" OR "environment" OR "physical activity" OR pollution OR "drug abuse" OR alcohol OR "substance abuse" OR "insecticide quality" OR "smoking" OR "adverse events" OR “acupuncture” OR “transfusion” OR “attitudes” OR "electronic" OR "adverse events") AND TITLE-ABS-KEY ("false positive" OR "false negative" OR "false transmitter" OR "false transmitters" OR "false result" OR "false results" OR "sperm" OR "DNA" OR "genetic" OR "gene" OR "block" OR “stem cell” OR “intoxication” OR “sanitation” OR "herbal" OR "traditional" OR “dental” OR “tooth” OR “dentist” OR “veterinary” OR “animal” OR “animals”)	740140
#3	#1 AND NOT #2	3633
#4	TITLE-ABS-KEY ("deprived countries" OR "deprived country" OR "deprived nation" OR "deprived nations" OR "deprived population" OR "deprived populations" OR "deprived world" OR "developing countries" OR "developing country" OR "developing economies" OR "developing economy" OR "developing nation" OR "developing nations" OR "developing population" OR "developing populations" OR "developing world" OR "lami countries" OR "lami country" OR "less developed countries" OR "less developed country" OR "less developed economies" OR "less developed economy" OR "less developed nation" OR "less developed nations" OR "less developed population" OR "less developed populations" OR "less developed world" OR "lesser developed countries" OR "lesser developed country" OR "lesser developed economies" OR "lesser developed economy" OR "lesser developed nation" OR "lesser developed nations" OR "lesser developed population" OR "lesser developed populations" OR "lesser developed world" OR "LMIC" OR "LMICS" OR "low gdp" OR "low gnp" OR "low gross domestic" OR "low gross national" OR "low income countries" OR "low income country" OR "low income economies" OR "low income economy" OR "low income nation" OR "low income nations" OR "low income population" OR "low income populations" OR "lower gdp" OR "lower gnp" OR "lower gross domestic" OR "lower gross national" OR "lower income countries" OR "lower income country" OR "lower income economies" OR "lower income economy" OR "lower income nation" OR "lower income nations" OR "lower income population" OR "lower income populations" OR "middle income countries" OR "middle income country" OR "middle income economies" OR "middle income economy" OR "middle income nation" OR "middle income nations" OR "middle income population" OR "middle income populations" OR "poor countries" OR "poor country" OR "Poor Economies" OR "Poor Economy" OR "poor nation" OR "poor nations" OR "poor	3,531,7<br>99

© 2018 Ozawa S et al. *JAMA Network Open.*

	population" OR "poor populations" OR "poor world" OR "poorer countries" OR "poorer country" OR "Poorer Economies" OR "Poorer Economy" OR "poorer nation" OR "poorer nations" OR "poorer population" OR "poorer populations" OR "poorer world" OR "third world" OR "transitional countries" OR "transitional country" OR "Transitional Economies" OR "Transitional Economy" OR "under developed countries" OR "under developed country" OR "under developed economies" OR "under developed economy" OR "under developed nation" OR "under developed nations" OR "under developed population" OR "under developed populations" OR "under developed world" OR "under served countries" OR "under served country" OR "under served nation" OR "under served nations" OR "under served population" OR "under served populations" OR "under served world" OR "underdeveloped countries" OR "underdeveloped country" OR "underdeveloped economies" OR "underdeveloped economy" OR "underdeveloped nation" OR "underdeveloped nations" OR "underdeveloped population" OR "underdeveloped populations" OR "underdeveloped world" OR "underserved countries" OR "underserved country" OR "underserved nation" OR "underserved nations" OR "underserved population" OR "underserved populations" OR "underserved world") OR TITLE-ABS-KEY (afghanistan OR albania OR algeria OR "American Samoa" OR angola OR armenia OR azerbaijan OR bangladesh OR belarus OR byelarus OR belorussia OR belize OR benin OR bhutan OR bolivia OR bosnia OR botswana OR brazil OR bulgaria OR burma OR "Burkina Faso" OR burundi OR "Cabo Verde" OR "Cape verde" OR cambodia OR cameroon OR "Central African Republic" OR chad OR china OR colombia OR comoros OR comores OR comoro OR congo OR "Costa Rica" OR "Côte d'Ivoire" OR cuba OR djibouti OR dominica OR "Dominican Republic" OR ecuador OR egypt OR "El Salvador" OR eritrea OR ethiopia OR fiji OR gabon OR gambia OR gaza OR "Georgia Republic" OR georgian OR ghana OR grenada OR grenadines OR guatemala OR guinea OR "Guinea Bisau" OR guyana OR haiti OR herzegovina OR hercegovina OR honduras OR india OR indonesia OR iran OR iraq OR jamaica OR jordan OR kazakhstan OR kenya OR kiribati OR korea OR kosovo OR kyrgyz OR kirghizia OR kirghiz OR kirgizstan OR kyrgyzstan OR "Lao PDR" OR laos OR lebanon OR lesotho OR liberia OR libya OR macedonia OR madagascar OR malawi OR malay OR malaya OR malaysia OR maldives OR mali OR "Marshall Islands" OR mauritania OR mauritius OR mexico OR micronesia OR moldova OR mongolia OR montenegro OR morocco OR mozambique OR myanmar OR namibia OR nepal OR nicaragua OR niger OR nigeria OR pakistan OR palau OR panama OR "Papua New Guinea" OR paraguay OR peru OR philippines OR phillippines OR philipines OR phillipines OR principe OR romania OR rwanda OR ruanda OR samoa OR "Sao Tome" OR senegal OR serbia OR "Sierra Leone" OR "Solomon Islands" OR somalia OR "South Africa" OR "South Sudan" OR "Sri Lanka" OR "St Lucia" OR "St Vincent" OR sudan OR suriham OR suriname OR swaziland OR syria OR "Syrian Arab Republic" OR tajikistan OR tadzhikistan OR tadjikistan OR tadzhik OR tanzania OR thailand OR timor OR togo OR tonga OR tunisia OR turkey OR turkmen OR turkmenistan OR tuvalu OR uganda OR ukraine OR uzbek OR uzbekistan OR vanuatu OR vietnam OR "West Bank" OR yemen OR zambia OR zimbabwe)	
#5	#3 AND #4	376

Scopus before duplicates removed: 376
Scopus after duplicates removed: 209

© 2018 Ozawa S et al. *JAMA Network Open.*

Embase

#1b	'quality':ti AND (('quality' NEAR/3 medicine) OR ('quality' NEAR/3 medicines) OR ('quality' NEAR/3 medication) OR ('quality' NEAR/3 medications) OR ('quality' NEAR/3 drug) OR ('quality' NEAR/3 drugs) OR ('quality' NEAR/3 pills) OR ('quality' NEAR/3 tablets) OR ('quality' NEAR/3 pharmaceutical) OR ('quality' NEAR/3 pharmaceuticals))	5813
#2	'tooth disease' OR 'dental' OR 'dental procedure' OR 'herbal medicine' OR 'traditional healer' OR 'herbaceous agent' OR 'plant medicinal product' OR 'physical activity, capacity and performance' OR 'genetic procedures':ab,ti OR 'false positive' OR 'false negative' OR 'false transmitter' OR 'false transmitters' OR 'false result' OR 'false results' OR 'quality of life':ab,ti OR 'quality of reporting':ab,ti OR 'quality of care':ab,ti OR 'quality care':ab,ti OR 'care quality':ab,ti OR 'quality of service':ab,ti OR 'quality of services':ab,ti OR 'service quality':ab,ti OR 'quality of sleep' OR 'sleep quality' OR 'adherence':ab,ti OR 'diet quality' OR 'quality of diet' OR 'quality-adjusted':ab,ti OR 'quality of evidence':ab,ti OR 'healthcare quality':ab,ti OR 'health care quality':ab,ti OR 'quality of health care':ab,ti OR 'quality of healthcare':ab,ti OR 'quality of medical':ab,ti OR 'quality health':ab,ti OR 'quality of health':ab,ti OR 'institute for quality':ab,ti OR 'sperm' OR 'solvent quality' OR 'dna':ab,ti OR 'quality of study':ab,ti OR 'quality of studies':ab,ti OR 'milk':ab,ti OR 'protein':ab,ti OR 'proteins':ab,ti OR 'quality innovation':ab,ti OR 'quality attribute':ab,ti OR 'quality attributes':ab,ti OR 'education':ti OR 'teaching':ti OR 'quality constant':ab,ti OR 'genetic':ab,ti OR 'gene':ab,ti OR 'block' OR 'quality management':ab,ti OR 'management quality':ab,ti OR 'treatment quality':ab,ti OR 'quality of outcome':ab,ti OR 'quality research':ab,ti OR 'environment':ti OR 'stem cell':ab,ti OR 'intoxication' OR 'sanitation' OR 'herbal' OR 'traditional' OR 'retention':ab,ti OR 'ventilation' OR 'curriculum' OR 'environment':ab,ti OR 'physical activity' OR pollution OR 'drug abuse' OR alcohol OR 'substance abuse' OR 'insecticide':ti OR 'smoking' OR 'acupuncture' OR 'transfusion' OR 'attitudes' OR 'electronic':ab,ti OR 'adverse events':ab,ti	9619062
#3	#1 NOT #2	2919
#4	'deprived countries' OR 'deprived country' OR 'deprived nation' OR 'deprived nations' OR 'deprived population' OR 'deprived populations' OR 'deprived world' OR 'developing countries' OR 'developing country' OR 'developing economies' OR 'developing economy' OR 'developing nation' OR 'developing nations' OR 'developing population' OR 'developing populations' OR 'developing world' OR 'lami countries' OR 'lami country' OR 'less developed countries' OR 'less developed country' OR 'less developed economies' OR 'less developed economy' OR 'less developed nation' OR 'less developed nations' OR 'less developed population' OR 'less developed populations' OR 'less developed world' OR 'lesser developed countries' OR 'lesser developed country' OR 'lesser developed economies' OR 'lesser developed economy' OR 'lesser developed nation' OR 'lesser developed nations' OR 'lesser developed population' OR 'lesser developed populations' OR 'lesser developed world' OR 'lmic' OR 'lmics' OR 'low gdp' OR 'low gnp' OR 'low gross domestic' OR 'low gross national' OR 'low income countries' OR 'low income country' OR 'low income economies' OR 'low income economy' OR 'low income nation' OR 'low income nations' OR 'low income population' OR 'low income populations' OR 'lower gdp' OR 'lower gnp' OR 'lower gross domestic' OR 'lower gross national' OR 'lower income countries' OR 'lower income country' OR 'lower income economies' OR 'lower income economy' OR 'lower income nation' OR 'lower	155202

© 2018 Ozawa S et al. *JAMA Network Open.*

	income nations' OR 'lower income population' OR 'lower income populations' OR 'middle income countries' OR 'middle income country' OR 'middle income economies' OR 'middle income economy' OR 'middle income nation' OR 'middle income nations' OR 'middle income population' OR 'middle income populations' OR 'poor countries' OR 'poor country' OR 'poor economies' OR 'poor economy' OR 'poor nation' OR 'poor nations' OR 'poor population' OR 'poor populations' OR 'poor world' OR 'poorer countries' OR 'poorer country' OR 'poorer economies' OR 'poorer economy' OR 'poorer nation' OR 'poorer nations' OR 'poorer population' OR 'poorer populations' OR 'poorer world' OR 'third world' OR 'transitional countries' OR 'transitional country' OR 'transitional economies' OR 'transitional economy' OR 'under developed countries' OR 'under developed country' OR 'under developed economies' OR 'under developed economy' OR 'under developed nation' OR 'under developed nations' OR 'under developed population' OR 'under developed populations' OR 'under developed world' OR 'under served countries' OR 'under served country' OR 'under served nation' OR 'under served nations' OR 'under served population' OR 'under served populations' OR 'under served world' OR 'underdeveloped countries' OR 'underdeveloped country' OR 'underdeveloped economies' OR 'underdeveloped economy' OR 'underdeveloped nation' OR 'underdeveloped nations' OR 'underdeveloped population' OR 'underdeveloped populations' OR 'underdeveloped world' OR 'underserved countries' OR 'underserved country' OR 'underserved nation' OR 'underserved nations' OR 'underserved population' OR 'underserved populations' OR 'underserved world'	
#5	afghanistan OR albania OR algeria OR 'american samoa' OR angola OR armenia OR azerbaijan OR bangladesh OR belarus OR byelarus OR belorussia OR belize OR benin OR bhutan OR bolivia OR bosnia OR botswana OR brazil OR bulgaria OR burma OR 'burkina faso' OR burundi OR 'cabo verde' OR 'cape verde' OR cambodia OR cameroon OR 'central african republic' OR chad OR china OR colombia OR comoros OR comores OR comoro OR congo OR 'costa rica' OR 'cote d ivoire' OR cuba OR djibouti OR dominica OR 'dominican republic' OR ecuador OR egypt OR 'el salvador' OR eritrea OR ethiopia OR fiji OR gabon OR gambia OR gaza OR 'georgia republic' OR georgian OR ghana OR grenada OR grenadines OR guatemala OR guinea OR 'guinea bisau' OR guyana OR haiti OR herzegovina OR hercegovina OR honduras OR india OR indonesia OR iran OR iraq OR jamaica OR jordan OR kazakhstan OR kenya OR kiribati OR korea OR kosovo OR kyrgyz OR kirghizia OR kirghiz OR kirgizstan OR kyrgyzstan OR 'lao pdr' OR laos OR lebanon OR lesotho OR liberia OR libya OR macedonia OR madagascar OR malawi OR malay OR malaya OR malaysia OR maldives OR mali OR 'marshall islands' OR mauritania OR mauritius OR mexico OR micronesia OR moldova OR mongolia OR montenegro OR morocco OR mozambique OR myanmar OR namibia OR nepal OR nicaragua OR niger OR nigeria OR pakistan OR palau OR panama OR 'papua new guinea' OR paraguay OR peru OR philippines OR phillippines OR philipines OR phillipines OR principe OR romania OR rwanda OR ruanda OR samoa OR 'sao tome' OR senegal OR serbia OR 'sierra leone' OR 'solomon islands' OR somalia OR 'south africa' OR 'south sudan' OR 'sri lanka' OR 'st lucia' OR 'st vincent' OR sudan OR suriham OR suriname OR swaziland OR syria OR 'syrian arab republic' OR tajikistan OR tadzhikistan OR tadjikistan OR tadzhik OR tanzania OR thailand OR timor OR togo OR tonga OR tunisia OR turkey OR turkmen OR turkmenistan OR tuvalu OR uganda OR ukraine OR uzbek OR uzbekistan OR vanuatu OR vietnam OR 'west bank' OR	5641628

© 2018 Ozawa S et al. *JAMA Network Open.*

	yemen OR zambia OR zimbabwe	
#6	(#1b NOT #2) AND (#3 OR #4)	879
#7	#6 AND [embase]/lim NOT [medline]/lim	566

Embase before duplicates removed: 566
Embase after duplicates removed: 392

Global Health

S1	TI (((quality) N3 (medicine OR medicines OR medication OR medications OR drug OR drugs OR pharmaceutical OR pharmaceuticals))))	305
S2	TI ("quality of life" OR "quality of reporting" OR "quality of care" OR "quality care" OR "care quality" OR "quality of service" OR "quality of services" OR "service quality" OR "quality of sleep" OR "sleep quality" OR “adherence” OR "diet quality" OR "quality of diet" OR "quality-adjusted" OR "quality of evidence" OR "healthcare quality" OR "health care quality" OR "quality of health care" OR "quality of healthcare" OR "quality of medical" OR "quality health" OR "quality of health" OR "institute for quality" OR "documentation quality" OR "bone quality" OR "solvent quality" OR "quality of study" OR "quality of studies" OR “milk” OR “protein” OR “proteins” OR "quality innovation" OR "quality attribute" OR "quality attributes" OR "quality education" OR "quality educational" OR "education quality" OR "quality of teaching" OR "teaching quality" OR "quality of instruction" OR "quality constant" OR "quality management" OR "management quality" OR "treatment quality" OR "quality of outcome" OR "quality research" OR "quality of data" OR "data quality" OR “air quality” OR "environment" OR "retention" OR "ventilation" OR "curriculum" OR "environment" OR "physical activity" OR pollution OR "drug abuse" OR alcohol OR "substance abuse" OR "insecticide quality" OR "smoking" OR "adverse events" OR “acupuncture” OR “transfusion” OR “attitudes” OR "electronic" OR "adverse events" OR "sperm" OR "DNA" OR "genetic" OR "gene" OR "block" OR “stem cell” OR “intoxication” OR “sanitation” OR "herbal" OR "traditional" OR “dental” OR “tooth” OR “dentist” OR “veterinary” OR “animal” OR “animals”)	335,740
S3	TI (("false positive" OR "false negative" OR "false transmitter" OR "false transmitters" OR "false result" OR "false results")) OR AB (("false positive" OR "false negative" OR "false transmitter" OR "false transmitters" OR "false result" OR "false results")) OR SU (("false positive" OR "false negative" OR "false transmitter" OR "false transmitters" OR "false result" OR "false results"))	9,523
S4	S1 NOT (S2 OR S3)	188

Global Health before duplicates removed: 188
Global Health after duplicates removed: 144

© 2018 Ozawa S et al. *JAMA Network Open.*

Additional Sources Searched

Total from additional sources before duplicates removed: 192
Total from additional sources after duplicates removed: 182

WWARN Database (Constrained to only peer-reviewed publications):
WWARN before duplicates removed: 93
WWARN after duplicates removed: 83

Similar Literature and Systematic Reviews:
Total Reviews searched: 12

Citations:

1. Almuzaini T, Choonara I, Sammons H. Substandard and counterfeit medicines: a systematic review of the literature. *BMJ open.* 2013;3(8):e002923.
2. Amin AA, Kokwaro GO. Antimalarial drug quality in Africa. *Journal of clinical pharmacy and therapeutics.* 2007;32(5):429-440.
3. Caudron JM, Ford N, Henkens M, Mace C, Kiddle-Monroe R, Pinel J. Substandard medicines in resource-poor settings: a problem that can no longer be ignored. *Tropical medicine & international health : TM & IH.* 2008;13(8):1062-1072.
4. Conway J, Bero L, Ondari C, Wasan KM. Review of the quality of pediatric medications in developing countries. *Journal of pharmaceutical sciences.* 2013;102(5):1419-1433.
5. Karunamoorthi K. The counterfeit anti-malarial is a crime against humanity: A systematic review of the scientific evidence. *Malaria Journal.* 2014;13(1).
6. Kelesidis T, Falagas ME. Substandard/counterfeit antimicrobial drugs. *Clinical Microbiology Reviews.* 2015;28(2):443-464.
7. Kelesidis T, Kelesidis I, Rafailidis PI, Falagas ME. Counterfeit or substandard antimicrobial drugs: a review of the scientific evidence. *Journal of Antimicrobial Chemotherapy.* 2007;60(2):214-236.
8. Nayyar GM, Breman JG, Newton PN, Herrington J. Poor-quality antimalarial drugs in southeast Asia and sub-Saharan Africa. *The Lancet Infectious diseases.* 2012;12(6):488-496.
9. Newton PN, Green MD, Fernandez FM, Day NP, White NJ. Counterfeit anti-infective drugs. *The Lancet Infectious diseases.* 2006;6(9):602-613.
10. Tabernero P, Fernández FM, Green M, Guerin PJ, Newton PN. Mind the gaps-the epidemiology of poor-quality anti-malarials in the malarious world-analysis of the WorldWide Antimalarial Resistance Network database. *Malaria journal.* 2014;13(1):139.
11. Torloni MR, Gomes Freitas C, Kartoglu UH, Metin Gulmezoglu A, Widmer M. Quality of oxytocin available in low- and middle-income countries: a systematic review of the literature. *BJOG : an international journal of obstetrics and gynaecology.* 2016;123(13):2076-2086.
12. World Health Organization. *A study on the public health and socioeconomic impact of substandard and falsified medical products.* Geneva2017.

Additions from other reviews: 49

Other databases searched:

WHO Essential Medicines and Health Products Information Portal: 6
USP Medicines Quality Database: 2
Reference Mining of Included Studies: 42

eReferences 1: Systematic Review Studies Not Included in Meta-analysis

Systematic Review (Not included in meta-analysis):

169 Studies

1. Abdelkarim A, Mohammed A, Abdeen M. Assessment of pharmaceutical quality control and in vitro equivalence of various brands of atenolol (100MG) tablets available in sudanese market under biowaiver conditions. *International Journal of Pharmaceutical Sciences and Research.* 2015;6(6):2390-2394.
2. Abdi YA, Rimoy G, Ericsson O, Alm C, Massele AY. Quality of chloroquine preparations marketed in Dar es Salaam, Tanzania. *Lancet (London, England).* 1995;346(8983):1161.
3. Abdullah M, Bepary S, Rouf A. Report: in vitro dissolution studies of different brands of sustained release diclofenac sodium matrix tablet available in Bangladesh. *Pakistan journal of pharmaceutical sciences.* 2008;21(1):70-77.
4. Abuga K, Amugune B, Ndwigah S, et al. Quality Performance of Drugs Analyzed in the Drug Analysis and Research Unit (DARU) during the Period 2006-2010. *East and Central African Journal of Pharmaceutical Sciences.* 2013;16(2):33-43.
5. Abuga KO, Mwagiru PM, Thoithi GN, et al. Quality of antiretroviral drugs analyzed in the drug analysis and research unit during 2000-2003. *East and Central African Journal of Pharmaceutical Sciences.* 2003;6(1):20-23.
6. Adegbite AI, Adegbolagun OM. Evaluation of the physicochemical equivalence of three brands of commercially available quinine sulphate tablets from South Western part of Nigeria. *African health sciences.* 2011;11(2).
7. Affum AO, Lowor S, Osae SD, Dickson A, Gyan BA, Tulasi D. A pilot study on quality of artesunate and amodiaquine tablets used in the fishing community of Tema, Ghana. *Malaria journal.* 2013;12:220.
8. Aina B, Tayo F, Taylor O. Quality of chloroquine dosage forms in Lagos State General Hospitals, Nigeria. *Journal of Pharmacy and Pharmacology.* 2007;59:119.
9. Ajala TO, Adebona AC, Bamiro OA. The pharmaceutical quality of brands of metformin tablets in Ogun-state, Nigeria. *African Journal of Biomedical Research.* 2014;17(1):43-48.
10. Akgeyik E, Kaynak MS, Çelebier M, Altınöz S, Şahin S. Evaluation of pharmaceutical quality of conventional dosage forms containing paracetamol and caffeine available in the Turkish drug market. *Dissolution Technologies.* 2016;23(2):36-41.
11. Akpabio E, Jackson C, Ugwu C, Etim M, Udofia M. Quality control and in vitro bioequivalence studies on four brands of ciprofloxacin tablets commonly sold in Uyo Metropolis, Nigeria. *Journal of Chemical and Pharmaceutical Research.* 2011;3(3):734-741.
12. Albonico M, Mathema P, Montresor A, et al. Comparative study of the quality and efficacy of originator and generic albendazole for mass treatment of soil-transmitted nematode infections in Nepal. *Transactions of the Royal Society of Tropical Medicine and Hygiene.* 2007;101(5):454-460.
13. Ali SA, Ezika CC, Abdulraheem OR, et al. Quality assessment of Artemisinin-based drugs marketed and used in Maiduguri Metropolitan Council Borno State, Nigeria. *Research Journal of Pharmaceutical, Biological and Chemical Sciences.* 2011;2(2):615-623.
14. Almarzouki A, Omar K, Elmajiri M, Alsaadi M. Comparative evaluation of different brands of enteric-coated diclofenac sodium tablets. *International Journal of Pharmaceutical Sciences and Research.* 2015;6(9):3829-3835.
15. Antignac M, Diop BI, Macquart De Terline D, et al. Quality assessment of 7 cardiovascular drugs in sub-Saharan African countries: Results of the seven study by drug and version of drug. *Global Heart.* 2016;11(2):e28.
16. Antignac M, Diop IB, Bernard M, et al. Quality of cardiovascular drugs in street markets and pharmacies in 10 sub-saharan African countries: The seven study. *European Heart Journal.* 2015;36:540.

© 2018 Ozawa S et al. *JAMA Network Open.*

17. Arnet I, Altermatt M, Roggo Y, Schnetzler G. Pharmaceutical quality of eight generics of ceftriaxone preparation for injection in Eastern Asia. *Journal of chemotherapy (Florence, Italy).* 2015;27(6):337-342.
18. Arshad HM, Shoaib MH, Ghayas S, Yousuf RI, Hanif M, Khan A. Pharmaceutical quality control studies on gatifloxacin 200 mg tablets available in the Pakistani market. *Latin American Journal of Pharmacy.* 2011;30(10):1922.
19. Atemnkeng M, Chimanuka B, Plaizier□Vercammen J. Quality evaluation of chloroquine, quinine, sulfadoxine–pyrimethamine and proguanil formulations sold on the market in East Congo DR. *Journal of clinical pharmacy and therapeutics.* 2007;32(2):123-132.
20. Atemnkeng MA, De Cock K, Plaizier-Vercammen J. Quality control of active ingredients in artemisinin-derivative antimalarials within Kenya and DR Congo. *Tropical medicine & international health : TM & IH.* 2007;12(1):68-74.
21. Audu S, Taiwo A, Saidu K. Quantitative analysis of ten (10) different brands of chlorpheniramine tablet marketed in Maiduguri metropolitan council (MMC). *J Chem Pharm Res.* 2012;4(7):3637-3650.
22. Awemu G, Ramos G, Tejano G, Maduka G. Chloroquine Content in Tablets, Syrups, and Injections of Some Common Brands in Nigeria. 2015.
23. Awemu GO, LO, Ajidue, TN. . Quality Control Studies on Multi-Sourced Aspirin Tablets Manufactured in Nigeria. *African Journal of Pharmaceutical Research & Development.* 2013;5(4):105-108.
24. Awofisayo SO, Awofisayo OA, Eyen N, Udoh IE. Comparative assessment of the quality control measurements of multisource ofloxacin tablets marketed in Nigeria. *Dissolution Technologies.* 2010;17(2):20-25.
25. Azam MG, Haider SS. Evaluation of dissolution behavior of paracetamol suspensions. *Dhaka University Journal of Pharmaceutical Sciences.* 2008;7(1):53-58.
26. Baba-Moussa F, Bonou J, Ahouandjinou H, et al. Quality Control of Selected Antimalarials Sold in the Illicit Market: An Investigation Conducted in Porto-Novo City (Republic of Benin). *Advances in Bioscience and Biotechnology.* 2015;6(10):637.
27. Bamiro O, Odeniyi M, Osonuga O. Brand variations in the physicochemical properties of metronidazole tablets. *Nigerian quarterly journal of hospital medicine.* 2007;17(1):22-25.
28. Basha B, Malaj L, Myftari E. A comparative study on the quality of different olmesartan tablets available in the albanian market. *Asian Journal of Pharmaceutical and Clinical Research.* 2016;9(2):80-83.
29. Bate R, Coticelli P, Tren R, Attaran A. Antimalarial Drug Quality in the Most Severely Malarious Parts of Africa – A Six Country Study. *PloS one.* 2008;3(5):e2132.
30. Bate R, Hess K. Anti-malarial drug quality in Lagos and Accra-a comparison of various quality assessments. *Malaria journal.* 2010;9(1):157.
31. Bate R, Jin GZ, Mathur A. Does price reveal poor-quality drugs? Evidence from 17 countries. *Journal of health economics.* 2011;30(6):1150-1163.
32. Bate R, Tren R, Hess K, Mooney L, Porter K. Pilot study comparing technologies to test for substandard drugs in field settings. *African Journal of Pharmacy and Pharmacology.* 2009;3(4):165-170.
33. Bate R, Tren R, Mooney L, et al. Pilot study of essential drug quality in two major cities in India. *PloS one.* 2009;4(6):e6003.
34. Beck RCR, Athayde ML, Cardoso SG. HIV/AIDS treatment and physicochemical quality control of medicines: Evaluation of non-generic lamivudine + zidovudine tablets manufactured in Brazil. *Brazilian Journal of Infectious Diseases.* 2007;11(6):540-543.
35. Bekoe SO, Bak SA, Björklund E, et al. Determination of thirteen antibiotics in drug products-A new LC-MS/MS tool for screening drug product quality. *Analytical Methods.* 2014;6(15):5847-5855.
36. Betari N, Haidar S. Pharmaceutical quality of generic sitagliptin tablets compared with Januvia®. *Journal of Applied Pharmacy.* 2014;6(2):195-201.
37. Birhanu G, Kassa F, Hymete A, Ashenef A. Comparative in-vitro quality evaluation of erythromycin stearate tablets marketed in Addis Ababa, Ethiopia. *International Journal of Pharmaceutical Sciences and Research.* 2013;4(1):378-385.

© 2018 Ozawa S et al. *JAMA Network Open.*

38. Briesen S, Guthoff R, Kilangalanga N, Mundkowski RG. [Counterfeiting of eye drops?: a laboratory study of antibiotic eye drops purchased in two African countries]. *Der Ophthalmologe : Zeitschrift der Deutschen Ophthalmologischen Gesellschaft.* 2012;109(12):1207-1210.
39. Bunhak EJ, Stoef PR, de Melo EB. Physicochemical quality evaluation of acetylsalicylic acid tablets distributed by a basic pharmacy. *Latin American Journal of Pharmacy.* 2010;29(8):1358-1363.
40. Chang F, Zhou Y. Grey clustering analysis for evaluating quality of medicines. *Chinese Journal of New Drugs.* 2014;23(2):130-133.
41. Chepkwony HK, Mwaura N, Guantai E, et al. Quality of antimalarial drugs analysed in the National Quality Control Laboratory during the period 2002-2005. *East and Central African Journal of Pharmaceutical Sciences.* 2007;10(3):59-62.
42. Çomoğlu T, Gönül N. Quality control studies on conventional carbamazepine tablets available on the Turkish drug market. *Turkish Journal of Medical Sciences.* 2005;35(4):217-221.
43. Cong LD, Yen PT, Van Nhu T, Binh LN. Use and quality of antimalarial drugs in the private sector in Viet Nam. *Bulletin of the World Health Organization.* 1998;76(SUPPL. 1):51-58.
44. Diop A, Sarr SO, Diop YM, Ndiaye B, Fall M, Mbaye G. [Quality control of antibiotics used in Senegal]. *Medecine tropicale : revue du Corps de sante colonial.* 2009;69(3):251-254.
45. Djobet MP, Singhe D, Lohoue J, Kuaban C, Ngogang J, Tambo E. Antiretroviral therapy supply chain quality control and assurance in improving people living with HIV therapeutic outcomes in Cameroon. *AIDS research and therapy.* 2017;14(1):19.
46. Ehianeta T, Williams B, Surakat J, Mohammed N, Anyakora C. Quality survey of some brands of artesunate-amodiaquine in Lagos drug market. *African Journal of Pharmacy and Pharmacology.* 2012;6(9):636-642.
47. Eichie FE, Arhewoh IM, Ezeobi OC. In-vitro evaluation of the pharmaceutical quality of some ibuprofen tablets dispensed in Nigeria. *African Journal of Pharmacy and Pharmacology.* 2009;3(10):491-495.
48. Eichie FE, Arhewoh MI, Isesele JE, Olatunji KT. In vitro assessment of quality control parameters of some commercially available generics of amlodipine besylate in Nigerian drug market. *International Journal of Health Research.* 2011;4(1):57-61.
49. El-Duah M, Ofori-Kwakye K. Substandard artemisinin-based antimalarial medicines in licensed retail pharmaceutical outlets in Ghana. *Journal of vector borne diseases.* 2012;49(3):131-139.
50. El-Sayed A, Boraie NA, Ismail FA, El-Khordagui LK, Khalil SA. Assessment of the pharmaceutical quality of omeprazole capsule brands marketed in Egypt. *Eastern Mediterranean health journal = La revue de sante de la Mediterranee orientale = al-Majallah al-sihhiyah li-sharq al-mutawassit.* 2007;13(6):1427-1437.
51. Eraga SO, Arhewoh MI, Chibuogwu RN, Iwuagwu MA. A comparative UV− HPLC analysis of ten brands of ibuprofen tablets. *Asian Pacific Journal of Tropical Biomedicine.* 2015;5(10):880-884.
52. Fadeyi I, Lalani M, Mailk N, Van Wyk A, Kaur H. Quality of the antibiotics--amoxicillin and co-trimoxazole from Ghana, Nigeria, and the United Kingdom. *The American journal of tropical medicine and hygiene.* 2015;92(6 Suppl):87-94.
53. Feroz M, Razvi N, Ghayas S, Anjum F, Ghazal L, Siddiqui SA. Assessment of pharmaceutical quality control and equivalence of various brands of amlodipine besylate (5 MG) tablets available in the Pakistani market under biowaiver conditions. *International Journal of Pharmacy and Pharmaceutical Sciences.* 2014;6(SUPPL. 2):909-913.
54. Fu Q, Li DK, Wang DY. Quality evaluation of famotidine products for injection. *Chinese Pharmaceutical Journal.* 2003;38(8):617-618.
55. Gaudiano MC, Di Maggio A, Cocchieri E, et al. Medicines informal market in Congo, Burundi and Angola: counterfeit and sub-standard antimalarials. *Malaria journal.* 2007;6:22.
56. Ghosal K, Ranu S, Billah M, Mondal S, Hoque M. Quality control studies on conventional carbamazepine tablets available in the Indian drug market. *Dissolution Technologies.* 2012;19(1):36-39.
57. Giri TK, Barwey N, Verma S, et al. Comparative assessment of the quality measurement of some commercially available paracetamol tablets. *International Journal of Pharmaceutical Sciences Review and Research.* 2012;14(2):42-46.

© 2018 Ozawa S et al. *JAMA Network Open.*

58. Giri TK, Sahu R, Kumar K, et al. In-vitro quality control measurement of some commercially available sustained release tablet containing diclofenac sodium. *Research Journal of Pharmacy and Technology.* 2012;5(5):687-690.
59. Greter H, Cowan N, Ngandolo BN, et al. Treatment of human and livestock helminth infections in a mobile pastoralist setting at Lake Chad: Attitudes to health and analysis of active pharmaceutical ingredients of locally available anthelminthic drugs. *Acta tropica.* 2016.
60. Gupta MM, Gupta M. Comparative pharmaceutical quality control testing of different brands of paracetamol tablets available in the Trinidad & Tobago, West Indies. *International Journal of Pharmaceutical Sciences and Research.* 2016;7(7):2830-2836.
61. Gyanwali P, Humagain BR, Aryal KK, et al. Surveillance of Quality of Medicines Available in the Nepalese Market: A Study from Kathmandu Valley. *Journal of Nepal Health Research Council.* 2015;13(31):233-240.
62. Hebron Y, Tettey JN, Pournamdari M, Watson DG. The chemical and pharmaceutical equivalence of sulphadoxine/pyrimethamine tablets sold on the Tanzanian market. *Journal of clinical pharmacy and therapeutics.* 2005;30(6):575-581.
63. Hehonah N, Popon J, Willie N. Using High Performance Liquid Chromatography to Quantitate Artemether and Artesunate Anti-Malarial Tablets in the National Capital District, Papua New Guinea. *Pacific Journal of Medical Science.* 2010;7(1).
64. Hernandez-Abad VJ, Castaneda-Hernandez G, Garcia-Jimenez S, Marroquin-Segura R, Sanchez-Gonzalez E. Evaluation of the quality of four Mexican drug products containing sodium naproxen. *Journal of clinical pharmacy and therapeutics.* 2008;33(3):237-242.
65. Hogerzeil HV, Walker G, De Goeje M, Organization WH. Stability of injectable oxytocics in tropical climates: results of field surveys and simulation studies on ergometrine, methylergometrine and oxytocin. 1993.
66. Höllein L, Kaale E, Mwalwisi YH, Schulze MH, Holzgrabe U. Routine quality control of medicines in developing countries: analytical challenges, regulatory infrastructures and the prevalence of counterfeit medicines in Tanzania. *TrAC Trends in Analytical Chemistry.* 2016;76:60-70.
67. Hossain MG, Rafiquzzaman M, Hoque MM. Quality and stability studies of marketed antibiotic dry syrups in Bangladesh - Amoxicillin a case study. *Indian Drugs.* 1999;36(8):529-530.
68. Iwuagwu M, Onyeonwu N. In vitro assessment of ampicillin capsules marketed in Nigeria. *International Journal of Pharmacy Practice.* 1992;1(3):167-171.
69. Izevbekhai O, Adeagbo B, Olagunju A, Bolaji O. Quality of artemisinin-based antimalarial drugs marketed in Nigeria. *Transactions of the Royal Society of Tropical Medicine and Hygiene.* 2017;111(2):90-96.
70. Jande M, Ngassapa O, Kibwage IO. Quality of sulfadoxine/pyrimethamine tablets marketed in Dar es Salaam, Tanzania. *East Cent Afr J Pharm Sci* 2000;3:20-24.
71. Joshua AU, Jimmy C, Uchechi AM, et al. Quality of Antimalarial Drugs in Malaria Endemic Municipalities of Pangasinan, Philippines. *International Journal of Malaria Research and Reviews.* 2016;4(1):1-6.
72. Kamuhabwa AAR, Saifuddin MH, Maganda B, Kaale E. Quality of co-trimoxazole tablets used for management of opportunistic infections in HIV/AIDS Patients. *International Journal of Pharmaceutical Research.* 2011;3(4):79-84.
73. Kar A, Amin MN, Hossain MS, Mukul MEH, Rashed MSU, Ibrahim M. Quality analysis of different marketed brands of paracetamol available in Bangladesh. 2015.
74. Karlage KL, Franklin SJ, Mufich WC, et al. Comparative evaluation of pharmaceutical products obtained in Mexico: Augmenting existing scientific data. *Drug Development and Industrial Pharmacy.* 2012;38(7):808-814.
75. Kaur H, Allan EL, Mamadu I, et al. Prevalence of substandard and falsified artemisinin-based combination antimalarial medicines on Bioko Island, Equatorial Guinea. *BMJ global health.* 2017;2(4):e000409.
76. Kaur H, Allan EL, Mamadu I, et al. Quality of artemisinin-based combination formulations for malaria treatment: prevalence and risk factors for poor quality medicines in public facilities and private sector drug outlets in Enugu, Nigeria. *PloS one.* 2015;10(5):e0125577.
77. Kaur H, Long J, Van Wyk A, et al. Assessing the quality of artemisinin combination therapies from Sub Saharan Africa and Cambodia, using analytical techniques. *Tropical Medicine and International Health.* 2013;18:21.

© 2018 Ozawa S et al. *JAMA Network Open.*

78. Kayumba PC, Risha PG, Shewiyo D, et al. The quality of essential antimicrobial and antimalarial drugs marketed in Rwanda and Tanzania: influence of tropical storage conditions on in vitro dissolution. *Journal of clinical pharmacy and therapeutics.* 2004;29(4):331-338.
79. Khan AN, Khar RK, Udayabanu M. Quality and affordability of amoxicillin generic products: A patient concern. *International Journal of Pharmacy and Pharmaceutical Sciences.* 2016;8(1):386-390.
80. Khan MH, Okumura J, Sovannarith T, Nivanna N, Akazawa M, Kimura K. Prevalence of counterfeit anthelminthic medicines: a cross-sectional survey in Cambodia. *Tropical medicine & international health : TM & IH.* 2010;15(5):639-644.
81. Khuluza F. In-vitro evaluation of the quality of paracetamol and co-trimoxazole tablets used in Malawi based on pharmacopoeial standards. *Malawi medical journal : the journal of Medical Association of Malawi.* 2014;26(2):38-41.
82. Khuluza F, Kigera S, Jahnke RW, Heide L. Use of thin-layer chromatography to detect counterfeit sulfadoxine/pyrimethamine tablets with the wrong active ingredient in Malawi. *Malaria journal.* 2016;15:215.
83. Kibwage IO, Ngugi J. Sulphadoxinel/pyrimethamine tablet products on the Kenyan market: Quality Concerns. *East Cent Afr J Pharm Sci* 2000;3:14-19.
84. Kibwage IO, Ogeto JO, Maitai CK, Rutere G, Thuranira J, Ochieng A. Drug quality control work in Daru: observations during 1983-1986. *East African medical journal.* 1992;69(10):577-580.
85. Kibwage IO, Thuranira J, Migosi D. Quality performance of metronidazole tablet products on the Kenyan market. *East African medical journal.* 1991;68(5):365-371.
86. Klein EY, Lewis IA, Jung C, Llinas M, Levin SA. Relationship between treatment-seeking behaviour and artemisinin drug quality in Ghana. *Malaria journal.* 2012;11:110.
87. Kumar S, Shashikant, Agnihotri R. In-vitro evaluation of two marketed brands of paracetamol tablets using quality control tests. *International Journal of Pharmaceutical Sciences and Research.* 2012;3(9):3337-3341.
88. Leslie T, Kaur H, Mohammed N, Kolaczinski K, Ord RL, Rowland M. Epidemic of Plasmodium falciparum malaria involving substandard antimalarial drugs, Pakistan, 2003. *Emerging infectious diseases.* 2009;15(11):1753-1759.
89. Lima LO, Benetoli A, Gianotto EAS, Fregonezi-Nery MM, Rabito MF. Quality evaluation of compounded capsules. *Latin American Journal of Pharmacy.* 2011;30(10):1943.
90. Longstaff C, Thelwell C, Whitton C. The poor quality of streptokinase products in use in developing countries. *Journal of thrombosis and haemostasis : JTH.* 2005;3(5):1092-1093.
91. Ma XN, Wen L, Liang S, et al. Quality analysis of domestic cefuroxime axetil tablets and capsules. *Chinese Journal of Antibiotics.* 2014;4:276-281.
92. Mac TL, Gaulier JM, Le VT, Vu AN, Preux PM, Ratsimbazafy V. Quality of antiepileptic drugs in Vietnam. *Epilepsy research.* 2008;80(1):77-82.
93. Macquart de Terline D, Diop BI, Bernard M, et al. Substandard drugs among five common antihypertensive generic medications: an analysis from 10 African countries. *Journal of hypertension.* 2017.
94. Maitai CK, Tsekpo WM, Wakori E, Wangia C, Mkoji L, Githiga IM. Drug quality control in Kenya--preliminary observations. *East African medical journal.* 1982;59(6):399-403.
95. Markman BE, Rosa PC, Koschtschak MR. Assessment of the quality of simvastatin capsules from compounding pharmacies. *Revista de saude publica.* 2010;44(6):1055-1062.
96. Marques-Marinhot FD, da Costa Zanonz JC, Sakurai E, Reis IA, Lima AA, Vianna-Soares CD. Quality evaluation of simvastatin compounded capsules. *Brazilian Journal of Pharmaceutical Sciences.* 2011;47(3):495-502.
97. Mei D, Fu Q, Li DK, Tian F, Zhang CG. Quality evaluation of different flunarizine preparations. *Chinese Pharmaceutical Journal.* 2002;37(2):135-138.
98. Ministry of Health K. Antimalarial Medicines in Kenya: Availability, Quality and Registration Status. 2007.
99. Minzi OM, Moshi MJ, Hipolite D, et al. Evaluation of the quality of amodiaquine and sulphadoxine/pyrimethamine tablets sold by private wholesale pharmacies in Dar Es Salaam Tanzania. *Journal of clinical pharmacy and therapeutics.* 2003;28(2):117-122.

© 2018 Ozawa S et al. *JAMA Network Open.*

100. Moe Moe A, Khin C, Min W, et al. Pharmaceutical equivalence and quality assurance of different types of quinolone antimicrobials available in Myanmar. *Myanmar Health Sciences Research Journal.* 2014;26(3):180-182.
101. Moses O, Patrick V, Muhammad N, Jasper OO, Celestino O. Substandard rifampicin based anti-tuberculosis drugs common in Ugandan drug market. *African Journal of Pharmacy and Pharmacology.* 2013;7(34):2428-2437.
102. Mubengayi C, Ramli Y, Routaboul C, Gilard V, Karbane M. Quality Evaluation of Diclofenac Formulations Manufactured in DR Congo. *Pharm Anal Chem Open Access.* 2016;2:112.
103. Mubengayi CK, El Karbane M, Mpona-Minga M, Cherrah Y, Essassi EM, Ramli Y. Quality evaluation of acetaminophen formulations manufactured in congo DR. *Journal of Chemical and Pharmaceutical Research.* 2013;5(7):322-328.
104. Musa H, Sule Y, Gwarzo M. Assessment of physicochemical properties of metronidazole tablets marketed in Zaria, Nigeria. *Int J Pharmacy Pharm Sci.* 2011;3(Suppl 3):27-29.
105. Musoke D, Obua C, Waako PJ. Assessment of the drug storage facilities and quality of generic co-formulation tablets (stavudine, lamivudine, nevirapine) at HIV/AIDS treatment centers in Uganda. *Research Journal of Medical Sciences.* 2008;2(3):159-162.
106. Nagaraj A, Tambi S, Shravani G, Biswas G, Kumawat H, Mathur G. Counterfeit medicines sale on online pharmacies in India. *Journal of research in pharmacy practice.* 2014;3(4):145-146.
107. Nair A, Strauch S, Lauwo J, Jahnke RW, Dressman J. Are counterfeit or substandard anti-infective products the cause of treatment failure in Papua New Guinea? *Journal of pharmaceutical sciences.* 2011;100(11):5059-5068.
108. Nayak AK. Comparative in vitro dissolution assessment of some commercially available paracetamol tablets. *Int J Pharm Sci Rev Res.* 2010;2:29-30.
109. Nesar S, Rahim N, Yousuf RI. Evaluation of pharmaceutical quality and bio waiver study of atenolol tablets available in Karachi (Pakistan). *Latin American Journal of Pharmacy.* 2017;36(10):2007-2011.
110. Newton P, Proux S, Green M, et al. Fake artesunate in southeast Asia. *Lancet (London, England).* 2001;357(9272):1948-1950.
111. Nightingale CH. A survey of the quality of generic clarithromycin products from 13 countries. *Clinical Drug Investigation.* 2000;19(4):293-305.
112. Nightingale CH. A survey of the quality of generic clarithromycin products from 18 countries. *Clinical Drug Investigation.* 2005;25(2):135-152.
113. Nnamdi JA, Arhewoh IM, Okhamafe AO, Enato EFO. Evaluation of the pharmaceutical quality of some quinine preparations sold in Nigeria. *Medical Principles and Practice.* 2009;18(3):193-197.
114. Nyarko EA, Nettey H. Quality Assessment of Artemether/Lumefantrine tablets sampled from pharmacies in Accra, using the MVHimagePCv8. exe color software. *Pharmacology & Pharmacy.* 2013;4(08):567.
115. Obodozie OO, Mustapha KB, Ebeshi BU, Inyang US. A comparative study on the prevalence of substandard ampicillin/cloxacillin preparations in the Nigerian market: Mid 1990's and present. *Journal of Phytomedicine and Therapeutics.* 2003;8-11:1-8.
116. Odeniyi MA, Adegoke OA, Adereti RB, Odeku OA, Itiola OA. Comparative analysis of eight brands of sulfadoxine-pyrimethamine tablets. *Tropical journal of pharmaceutical research.* 2003;2(1):161-167.
117. Odermatt P, Ly S, Simmala C, et al. Availability and costs of antiepileptic drugs and quality of phenobarbital in Vientiane municipality, Lao PDR. *Neuroepidemiology.* 2007;28(3):169-174.
118. Odunfa OO, Adegoke OA, Onaga IC. Pharmaceutical equivalence of some commercial samples of artesunate and amodiaquine tablets sold in Southwestern Nigeria. *Tropical Journal of Pharmaceutical Research.* 2009;8(6):491-499.
119. Ofonaike J, Enato EF, Okhamafe AO. A study of the pharmaceutical quality of chloroquine and paracetamol products sold in a major Nigerian “market”. *African Journal of Health Sciences.* 2007;14(3):164-170.

120. Ofori-Kwakye K, Asantewaa Y, Gaye O. Quality of artesunate tablets sold in pharmacies in Kumasi, Ghana. *Tropical Journal of Pharmaceutical Research.* 2008;7(4):1179-1184.
121. Okeke I, Lamikanra A. Quality and bioavailability of ampicillin capsules dispensed in a Nigerian semi-urban community. *African journal of medicine and medical sciences.* 2001;30(1-2):47-51.
122. Okeke IN, Lamikanra A. Quality and bioavailability of tetracycline capsules in a Nigerian semi-urban community. *International journal of antimicrobial agents.* 1995;5(4):245-250.
123. Okorie O, Okonkwo TJ. Inter-batch quality control (QC) and bioavailabilty variability of a branded artesunate tablet in Nigeria. *International Journal of Pharmaceutical Sciences Review and Research.* 2011;6(1):110-113.
124. Olanrewaju OJ, Paul AC, Olusola AM. Quality assessment of amoxicillin-clavulanate potassium tablets in Lagos, Nigeria. *Journal of Chemical and Pharmaceutical Research.* 2012;4(12):5032-5038.
125. Osei-Safo D, Egbo HA, Nettey H, Konadu DY, Addae-Mensah I. Evaluation of the quality of some antibiotics distributed in Accra and Lagos. *International Journal of Pharmaceutical Sciences and Research.* 2016;7(5):1991-2000.
126. Özyazici M, Baloğlu E. Quality control on fluconazole capsuls in Turkish drug market. *Acta Pharmaceutica Turcica.* 2001;43(2):101-105.
127. Patil D, Pandit V, Pore S, Chavan C. Fighting counterfeit and substandard drugs at periphery; the utility of basic quality control tests. Pharmacie Globale. *Int J Comprehen Pharm.* 2012;3:1-3.
128. Penzak SR, Acosta EP, Turner M, Tavel JA, Masur H. Analysis of generic nevirapine products in developing countriesresearch letter. *JAMA.* 2003;289(20):2648-2649.
129. Petralanda I. Quality of antimalarial drugs and resistance to Plasmodium vivax in Amazonian region. *The Lancet.* 1995;345(8962):1433.
130. Pichini S, Rotolo MC, Bellotti P, Minutillo A, Mastrobattista L, Pacifici R. Quali-quantitative analysis of best selling drugs from pharmacy, street market and traditional herbal medicine: a pilot study of market surveillance in Senegal. *Journal of pharmaceutical and biomedical analysis.* 2015;104:62-66.
131. Pierrot Mwamba T, Duez P, Jean Baptiste Kalonji N. Preliminary survey of counterfeiting of albendazole and metronidazole marketed in Lubumbashi. *International Journal of Pharmacy and Pharmaceutical Sciences.* 2016;8(7):282-287.
132. Pouplin T, Phuong PN, Van Toi P, Pouplin JN, Farrar J. Isoniazid, pyrazinamide and rifampicin content variation in split fixed-dose combination tablets. *PloS one.* 2014;9(7):e102047.
133. Prah J, Ameyaw EO, Afoakwah R, Fiawoyife P, Oppong-Danquah E, Boampong JN. Quality Assessment of Artemether-Lumefantrine Samples and Artemether Injections Sold in the Cape Coast Metropolis. *Journal of tropical medicine.* 2016;2016:8602619.
134. Prazuck T, Falconi I, Morineau G, Bricard-Pacaud V, Lecomte A, Ballereau F. Quality control of antibiotics before the implementation of an STD program in Northern Myanmar. *Sexually transmitted diseases.* 2002;29(11):624-627.
135. Rahman Dewan SM, Alam A, Koushik Ahamed S. A comparative quality control study on conventional ibuprofen tablets available in bangladeshi pharma market. *International Research Journal of Pharmacy.* 2013;4(1):96-98.
136. Risha PG, Shewiyo D, Msami A, et al. In vitro evaluation of the quality of essential drugs on the Tanzanian market. *Tropical medicine & international health : TM & IH.* 2002;7(8):701-707.
137. Roy J, Saha P, Sultana S, Kenyon AS. Rapid screening of marketed paracetamol tablets: use of thin-layer chromatography and a semiquantitative spot test. *Bulletin of the World Health Organization.* 1997;75(1):19-22.
138. Sahle SB, Ayane AT, Wabe NT. Comparative quality evaluation of paracetamol tablet marketed in somali region of Ethiopia. *International Journal of Pharmaceutical Sciences and Research.* 2012;3(2):545-550.
139. Said MM, Gibbons S, Moffat AC, Zloh M. Near-infrared spectroscopy (NIRS) and chemometric analysis of Malaysian and UK paracetamol tablets: a spectral database study. *International journal of pharmaceutics.* 2011;415(1-2):102-109.
140. Salih H, Hamam B. Comparative in vitro evaluation of generic ciprofloxacin hydrochloride tablets. *World J Pharm Pharm Sci.* 2014;38(12):388-396.

141. Samali A, Mohammed MI, Ibrahim MB, Gamaniel KS. Quality status of brands of chloroquine tablet dosage form from Northern part of Nigeria. *Journal of Chemical and Pharmaceutical Research.* 2014;6(11):75-79.
142. Sani AA, David BI, Alemika ET, Musa AS, Ilyas M. Quantitative analysis of different brands of dexamethasone tablet marketed in Maiduguri Metropolitan Council. *International Research Journal of Pharmacy.* 2012;3(9):96-105.
143. Sapsirisavat V, Vongsutilers V, Thammajaruk N, et al. Pharmaceutical Equivalence of Distributed Generic Antiretroviral (ARV) in Asian Settings: The Cross-Sectional Surveillance Study - PEDA Study. *PloS one.* 2016;11(6):e0157039.
144. Sarker MMR, Rashid MS, Raju AA, et al. Evaluation of the pharmaceutical quality of different brands of ranitidine tablets manufactured in Bangladesh: A pharmaceutical and public health prospective. *Journal of Applied Pharmaceutical Science.* 2016;6(1):055-061.
145. Sawadogo CW, Al-Kamarany MA, Al-Mekhlafi HM, et al. Quality of chloroquine tablets available in Africa. *Annals of Tropical Medicine and Parasitology.* 2011;105(6):447-453.
146. Sengaloundeth S, Green MD, Fernandez FM, et al. A stratified random survey of the proportion of poor quality oral artesunate sold at medicine outlets in the Lao PDR - implications for therapeutic failure and drug resistance. *Malaria journal.* 2009;8:172.
147. Shenoy Panchmal G, Shenoy R, Tellis R, et al. Comparison of medicine quality of the generic formulation of amoxicillin provided by the government of Karnataka with marketed brands - a public health perspective. *International Journal of Pharmaceutical Sciences Review and Research.* 2013;23(1):37-42.
148. Singal G, Nanda A, Kotwani A. A comparative evaluation of price and quality of some branded versus branded–generic medicines of the same manufacturer in India. *Indian journal of pharmacology.* 2011;43(2):131.
149. Singhal GL, Anita K, Nanda A. Jan Aushadhi stores in India and quality of medicines therein. *International Journal of Pharmacy and Pharmaceutical Sciences.* 2011;3(1):204-207.
150. Smine A. DKaBNLU. Antimalarial Drug Quality in Senegal 2002.
151. Sow P, Gueye T, Sy E, Toure L, Ba C, Badiane M. Drugs in the parallel market for the treatment of urethral discharge in Dakar: epidemiologic investigation and physicochemical tests. *International Journal of Infectious Diseases.* 2002;6(2):108-112.
152. Sulaiman SM, Traore M, Engels D, Hagan P, Cioli D. Counterfeit praziquantel. *Lancet (London, England).* 2001;358(9282):666-667.
153. Teklu L, Adugna E, Ashenef A. Quality evaluation of paracetamol tablets obtained from the common shops (kiosks) in addis ababa, ethiopia. *International Journal of Pharmaceutical Sciences and Research.* 2014;5(8):3502-3510.
154. Thoithi G, Abuga K, Nguyo J, et al. Drug quality control in Kenya: Observation in drug analysis and research unit during the period 1996-2000. *East and Central African Journal of Pharmaceutical Sciences.* 2002;5(2):28-32.
155. Thoithi GN, Abuga KO, Nguyo JM, et al. Drug quality control in Kenya: observation in the drug analysis and research unit during the period 2001-2005. *East and Central African Journal of Pharmaceutical Sciences.* 2008;11(3):un-un.
156. Trefi S, Gilard V, Malet-Martino M, Martino R. Generic ciprofloxacin tablets contain the stated amount of drug and different impurity profiles: a 19 F, 1 H and DOSY NMR analysis. *Journal of pharmaceutical and biomedical analysis.* 2007;44(3):743-754.
157. Twagirumukiza M, Cosijns A, Pringels E, Remon JP, Vervaet C, Van Bortel L. Influence of tropical climate conditions on the quality of antihypertensive drugs from Rwandan pharmacies. *The American journal of tropical medicine and hygiene.* 2009;81(5):776-781.
158. Uddin MS, Mamun AA, Hossain MS, et al. In vitro quality evaluation of leading brands of ciprofloxacin tablets available in Bangladesh. *BMC research notes.* 2017;10(1):185.
159. Vial J, Cohen M, Sassiat P, Thiébaut D. Pharmaceutical quality of docetaxel generics versus originator drug product: a comparative analysis. *Current medical research and opinion.* 2008;24(7):2019-2033.
160. Visser BJ, Vries SG, Bache EB, et al. The diagnostic accuracy of the hand-held Raman spectrometer for the identification of anti-malarial drugs. *Malaria journal.* 2016;15(1):160.
161. Welfare IMoHaF. A Comprehensive Examination of Drug Regulatory Issues, Including the Problem of Spurious Drugs. 2003;6000:27.

© 2018 Ozawa S et al. *JAMA Network Open.*

162. Xhafaj D, Malaj L, Mileti M. A comparative quality control study of cetirizine hydrochloride 10mg tablets on the Albanian pharmaceutical market. *International Journal of Pharmacy and Pharmaceutical Sciences.* 2015;7(1):504-507.
163. Xu Y, Li XG, Zhao RS. Quality evaluation of thioctic acid for injection. *Chinese Journal of New Drugs.* 2015;24(14):1624-1627 and 1674.
164. Xue J, Yin LH, Zhang R, Ma WL, Hu CQ. Comparison and evaluation for the quality of domestic and imported mycophenolate mofeti capsules. *Chinese Journal of New Drugs.* 2013;22(21):2553-2560.
165. Yao J, Shi YQ, Li ZR, Jin SH. Development of a RP-HPLC method for screening potentially counterfeit anti-diabetic drugs. *Journal of chromatography B, Analytical technologies in the biomedical and life sciences.* 2007;853(1-2):254-259.
166. Yeboah DF, Afoakwah R, Nwaefuna EK, Verner O, Boampong JN. Quality of sulfadoxine-pyrimethamine given as antimalarial prophylaxis in pregnant women in selected health facilities in central region of Ghana. *Journal of parasitology research.* 2016;2016.
167. Yong YL, Plancon A, Lau YH, et al. Collaborative health and enforcement operations on the quality of antimalarials and antibiotics in southeast Asia. *The American journal of tropical medicine and hygiene.* 2015;92(6 Suppl):105-112.
168. Yu T, Zhao RS, Zhou Y, Zhai SD. Quality evaluation of vancomycin hydrochloride for injection. *Chinese Pharmaceutical Journal.* 2009;44(21):1662-1665.
169. Zaheer M, Rahman S, Mahmood S, Saleem M. In vitro analysis and data comparison of market brands of ciprofloxacin, ofloxacin and levofloxacin. *Pak J Sci Ind Res.* 2009;52:186-190.

© 2018 Ozawa S et al. *JAMA Network Open.*

eAppendix 2: MEDQUARG Scores and Interrater Reliability

The Medicine Quality Assessment Reporting Guidelines (MEDQUARG) is a checklist proposed by Newton et al.[1] in 2009 to improve the quality of publications examining the quality of medicines. In 2013 Almuzaini et al.[2] took the MEDQUARG checklist and adapted it to a scoring metric in order to assign publications specific quality scores out of 12. The 12 criteria that each study is scored upon are as follows, with the study receiving one point for each criteria met. (Taken from Almuzaini et al. 2013[2])

1. Timing and location of study clearly stated.
2. Definition of counterfeit or substandard medicines used mentioned.
3. Type of outlets sampled.
4. Sampling design and sample size calculation described.
5. Type and number of dosage units purchased per outlet.
6. Random sampling used.
7. Information on who collected the samples (were mystery shoppers applied?)
8. Packaging assessment performed.
9. Statistical analysis described.
10. Chemical analysis clearly described.
11. Details on method validation.
12. Chemical analysis performed blinded to packaging.

To ensure that the studies in this review were assessed accurately according to the MEDQUARG scoring metric the studies were assessed independently by two reviewers (SL and DE) who were blinded to the scores assigned by Almuzaini et al. in their 2013 systematic review. Upon scoring all the studies individually the reviewers met and assigned a final MEDQUARG score to each study. The interrater reliability (IRR) between the two reviewers as well as the IRR between each reviewer and the final score was assessed using Spearman's Rho. The IRR for the MEDQUARG scores for studies included in this review that were also included in Almuzaini et al. was also examined. The results of the analyses showed a high levels of interrater reliability between both reviewers, the reviewers and the final score, and the final score and the Almuzaini et al. score (see results below).

© 2018 Ozawa S et al. *JAMA Network Open.*

eTable 1: MEDQUARG Scores of Studies Included in the Meta-analysis

Author (Year) [Citation #]	MEDQUARG SL	MEDQUARG DE	FINAL
Abdo-Rabbo et al (2005) [33]	5	4	4
ACT Consortium (2015) [34]	8	8	8
Amin et al (2005) [35]	4	4	4
Antignac et al (2017) [36]	10	10	10
Baratta et al (2012) [37]	5	5	5
Basco (2004) [38]	6	6	6
Bate et al (2010) [39]	5	4	4
Bate et al (2012) [40]	6	6	6
Bate et al (2013) [41]	7	8	7
Bate et al (2014) [42]	3	3	3
Bate et al (2015) [43]	6	7	6
Bjorkman et al (2012) [44]	10	10	10
Bruneton (1995) [45]	4	4	4
CDSCO (2009) [46]	6	6	6
Chikowe et al (2015) [47]	7	9	8
Dondorp et al (2004) [48]	7	8	7
Evans et al (2012) [49]	6	6	6
Fotiou et al (2009) [50]	4	5	4
Gimenez et al (1997) [51]	5	3	4
Guo et al (2017) [52]	5	5	5
Hadi et al (2010) [53]	6	7	6
Hajjou et al (2015) [54]	5	5	5
Hall (2016) [55]	4	4	4
Hetzel et al (2014) [56]	10	10	10
Idowu et al (2006) [57]	1	1	1
Ioset and Kaur (2009) [58]	3	3	3
Kaale et al (2016) [59]	6	6	6
Karikari-Boateng et al (2013) [60]	5	5	5
Kaur et al (2008) [61]	6	6	6
Kaur et al (2016) [62]	10	11	10
Kenyan Ministry of Public Health and Sanitation (2011) [63]	6	6	6
Kenyan Ministry of Public Health and Sanitation (2012) [64]	6	7	6
Khan et al (2011) [65]	7	7	7
Khan et al (2013) [66]	7	6	6
Khin et al (2016) [67]	7	6	6
Khuluza et al (2017) [68]	10	10	10
Khurelbat et al (2014) [69]	9	10	9
Kuwana et al (2017) [70]	3	3	3

© 2018 Ozawa S et al. *JAMA Network Open.*

Kyriacos et al (2008) [71]	4	4	4
Lalani et al (2015) [72]	6	8	7
Laroche et al (2005) [73]	6	6	6
Laserson et al (2001) [74]	3	5	4
Lon et al (2006) [75]	6	7	6
Maponga et al (2003) [76]	4	4	4
Mbaziira et al (2015) [77]	4	3	3
Nabirova et al (2017) [78]	6	6	6
Nazerali and Hogerzeil (1998) [79]	5	5	5
Newton et al (2001) [80]	7	5	6
Newton et al (2008) [81]	5	5	5
Obaid (2009) [82]	5	4	4
Ochekpe et al (2010) [83]	7	5	6
Ogwal-Okeng et al (1998) [84]	7	7	7
Ogwal-Okeng et al (2003) [85]	6	6	6
Okumura et al (2010) [86]	4	4	4
Onwujekwe et al (2009) [87]	7	7	7
Osei-Safo et al (2014) [88]	7	7	7
Patel et al (2012) [89]	6	6	6
Petersen et al (2017) [90]	7	7	7
Phanouvong et al (2013a) [91]	5	5	5
Phanouvong et al (2013b) [92]	6	6	6
Pouillot et al (2008) [93]	5	5	5
Pribluda et al (2012) [94]	5	5	5
Ramachandran et al (2013) [95]	5	4	4
Risha et al (2008) [96]	3	1	2
Rookkapan et al (2005) [97]	3	3	3
Roy et al (1993) [98]	3	2	2
Sabartova et al (2011) [99]	8	7	7
Seear et al (2011) [100]	7	6	6
Shakoor et al (1997) [101]	5	5	5
Sheth et al (2007) [102]	5	5	5
Stanton et al (2012) [103]	6	6	6
Stanton et al (2014) [104]	6	6	6
Stenson et al (1998) [105]	5	5	5
Suleman et al (2014) [106]	9	9	9
Syhakhang et al (2002) [107]	8	9	8
Syhakhang et al (2004) [108]	10	10	10
Tabernero et al (2015) [109]	10	11	10
Taylor et al (2001) [110]	5	6	5
Tipke et al (2008) [111]	6	7	6

Tivura et al (2016) [112]	9	10	9
Tshilumba et al (2015) [113]	5	5	5
Uganda Medicines Transparency Alliance (2014) [114]	5	5	5
Uganda Medicines Transparency Alliance (2015) [115]	6	6	6
Vijaykadga et al (2006) [116]	8	6	7
Visser et al (2015) [117]	10	11	10
Wafula et al (2016) [118]	3	4	3
Wahidullah et al (2011) [119]	7	7	7
Wang et al (2015) [120]	5	4	4
Wondemagegnehu (1999) [121]	5	5	5
World Health Organization (2007) [122]	5	5	5
World Health Organization (2009) [123]	7	7	7
World Health Organization (2011) [124]	4	4	4
World Health Organization (2016) [125]	6	5	5
Yang et al (2004) [126]	5	5	5
Yeung et al (2015) [127]	9	9	9
Yoshida et al (2014) [128]	10	8	9

© 2018 Ozawa S et al. *JAMA Network Open.*

Comparison to Almuzaini et al. MEDQUARG scores

**Almuzaini MEDQUARG Scores Author (Year)**	*SL*	*DE*	**FINAL**	**Almuzaini**
Abdo Rabbo et. al (2005)	5	4	4	3
Amin et. al (2005)	4	4	4	4
Baratta et. al (2012)	5	5	5	5
Basco et. al (2004)	6	6	6	6
Bate et. al (2009)	4	5	4	2
Bruneton et. al (1995)	4	4	4	5
Dondorp et. al (2004)	7	8	7	7
Hadi et. al (2010)	6	7	6	8
Kaur et. al (2008)	10	11	10	9
Kyriacos et. al (2008)	4	4	4	4
Laserson et. al (2001)	3	5	4	5
Lon et. al (2006)	6	7	6	6
Maponga et. al (2003)	4	4	4	6
Newton et. al (2001)	5	5	5	5
Newton et. al (2008)	7	5	5	5
Obaid et. al (2009)	5	4	4	3
Ogwal Okeng et. al (1998)	7	7	7	5
Ogwal Okeng et. al (2003)	6	6	6	6
Onwujekwe et. al (2009)	7	7	7	7
Pribluda et. al (2012)	5	5	5	4
Sabartova et. al (2011)	8	7	7	6
Seear et. al (2011)	7	6	6	5
Shakoor et. al (1997)	5	5	5	6
Stenson et. al (1998)	5	5	5	5
Syhakhang et. al (2004)	10	10	10	10
Taylor et. al (2001)	5	6	5	6
Tipke et. al (2008)	6	7	6	5
WHO (2009)	7	7	7	7
WHO (2011)	6	5	5	7
Wondemagegnehu et. al (1999)	5	5	5	6

© 2018 Ozawa S et al. *JAMA Network Open.*

Interrater Reliability

Between the final score from this review and those of Almuzaini et al.:

```
. *Spearman's Rho
. spearman final mqa

 Number of obs =      30
Spearman's rho =       0.7177

Test of Ho: final and mqa are independent
    Prob > |t| =       0.0000
```

Between the two reviewers' scores and Almuzaini et al.'s:

```
. spearman mqs mqa

 Number of obs =      30
Spearman's rho =       0.6218

Test of Ho: mqs and mqa are independent
    Prob > |t| =       0.0002

. spearman mqd mqa

 Number of obs =      30
Spearman's rho =       0.7030

Test of Ho: mqd and mqa are independent
    Prob > |t| =       0.0000
```

Between the two reviewers and each reviewer and final score:

```
. *Spearman's Rho
. spearman mqs mqd

 Number of obs =      96
Spearman's rho =       0.9185

Test of Ho: mqs and mqd are independent
    Prob > |t| =       0.0000

. spearman mqs final

 Number of obs =      96
Spearman's rho =       0.9711

Test of Ho: mqs and final are independent
    Prob > |t| =       0.0000

. spearman mqd final

 Number of obs =      96
Spearman's rho =       0.9714

Test of Ho: mqd and final are independent
    Prob > |t| =       0.0000
```

© 2018 Ozawa S et al. *JAMA Network Open.*

eAppendix 3: Meta-analysis Bias and Quality Analysis

Due to the significant differences in study methods, quality, location, sample types, chemical testing, etc. a large amount of disparity across the studies is expected. To examine study heterogeneity, bias, and potential moderators a random effects model was run for both the weighted and unweighted point prevalences. Heterogeneity was evaluated via Cochrane's Q and I^2, publication bias was analyzed using a funnel plot analysis, influence was determined by baujaut and influence plots, and eight potential moderators (MEDQUARG scores, MEDQUARG categorization, sample size, publication year, region, medicine category, use of random sampling, and use of mystery shoppers).

Heterogeneity: The studies had large amounts of heterogeneity with a significant Q value. The I^2 value for the model was very high (99.92%).

Influence: Influence plots on the unweighted point estimates indicated that studies [15], [71], and [94] had significant levels of influence. Each of these studies had prevalences of 85% or greater.

Publication Bias: To examine the studies for publication bias a funnel plot analysis was performed. Visual inspection of the plot indicated publication bias with studies with low standard errors having lower proportions of substandard/falsified samples. Statistical analysis of the funnel plot results was done via a regression test (significant).

Moderators: Number of samples tested was found to be a significant moderator. See results below.

3.1. Heterogeneity:

```
Random-Effects Model (k = 96; tau^2 estimator: REML)

tau^2 (estimated amount of total heterogeneity): 0.0442 (SE = 0.0066)
tau (square root of estimated tau^2 value):      0.2103
I^2 (total heterogeneity / total variability):   99.92%
H^2 (total variability / sampling variability):  1262.97

Test for Heterogeneity:
Q(df = 95) = 14181.4845, p-val < .0001

Model Results:

estimate      se     zval    pval   ci.lb   ci.ub
  0.2465  0.0217  11.3645  <.0001  0.2040  0.2890  ***

---
Signif. codes:  0 '***' 0.001 '**' 0.01 '*' 0.05 '.' 0.1 ' ' 1
```

```
          estimate     ci.lb     ci.ub
tau^2       0.0442    0.0337    0.0603
tau         0.2103    0.1835    0.2456
I^2(%)     99.9208   99.8960   99.9419
H^2      1262.9695  961.6433 1721.5317
```

© 2018 Ozawa S et al. *JAMA Network Open.*

3.2. Publication Bias:

eFigure 1: Funnel Plot

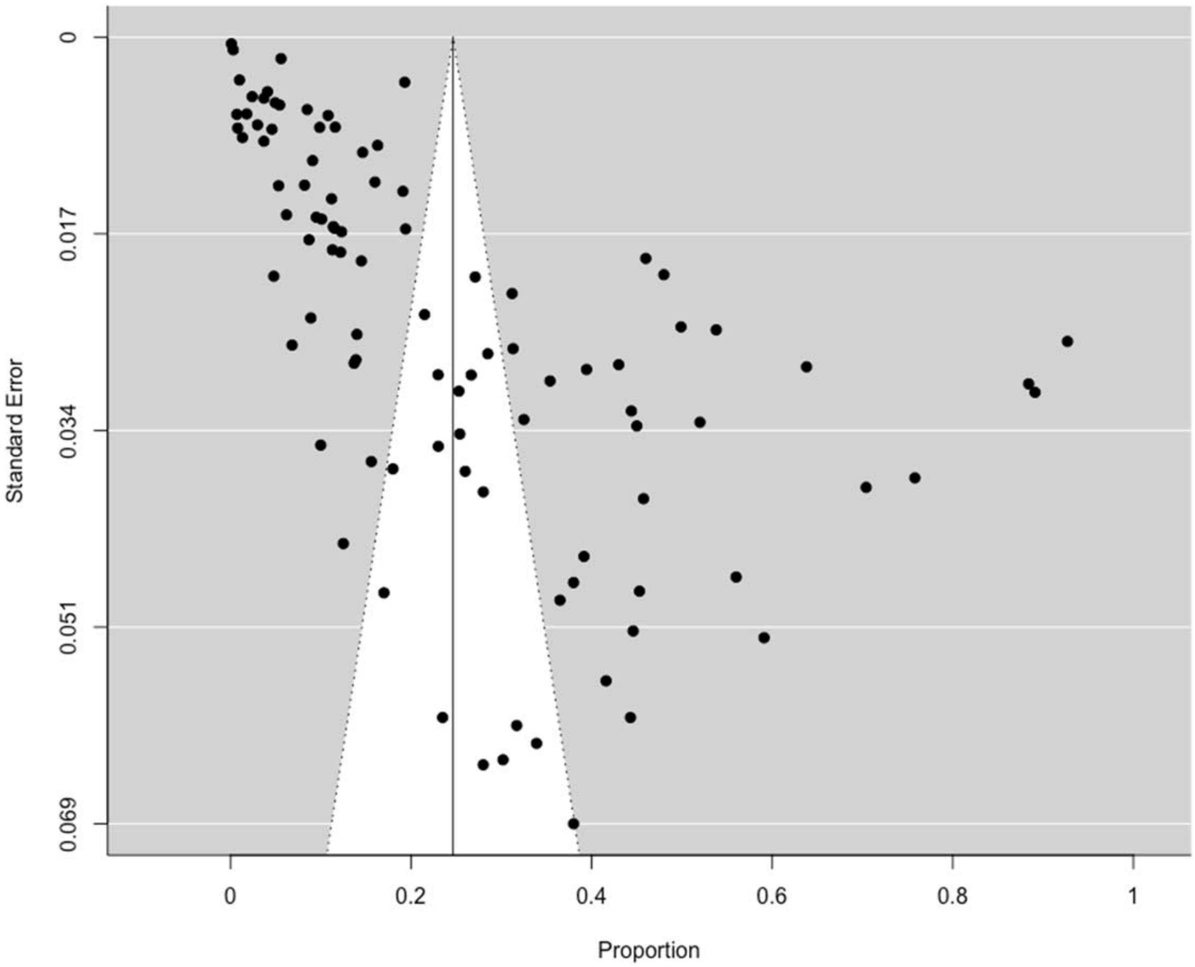


```
Regression Test for Funnel Plot Asymmetry

model:     mixed-effects meta-regression model
predictor: standard error

test for funnel plot asymmetry: z = 6.5633, p < .0001
```

© 2018 Ozawa S et al. *JAMA Network Open.*

3.3. Individual Study Influence on Heterogeneity:

eFigure 2: Baujat Plot

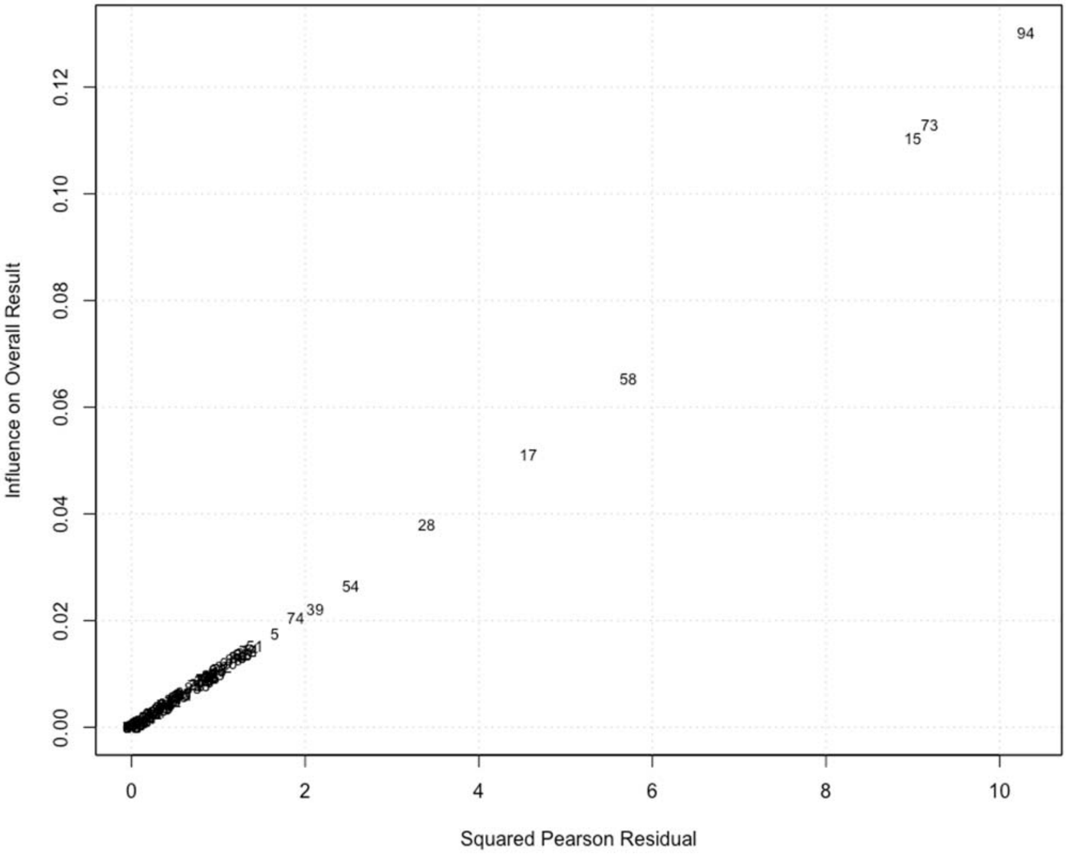

© 2018 Ozawa S et al. *JAMA Network Open.*

eFigure 3: Influence Plot and Output

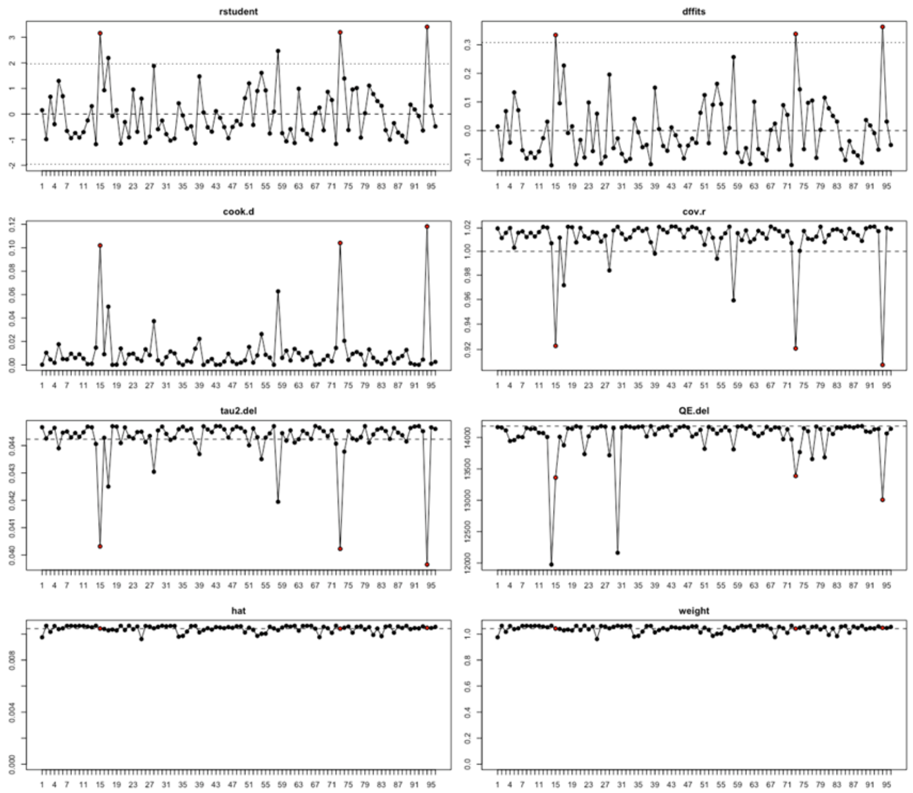

	rstudent	dffits	cook.d	cov.r	tau2.del	QE.del	hat	weight	dfbs	inf
1	0.1523	0.0142	0.0002	1.0196	0.0447	14164.41	0.0098	0.9750	0.0141	
2	-0.9819	-0.1019	0.0104	1.0114	0.0443	14157.06	0.0106	1.0633	-0.1019	
3	0.6754	0.0679	0.0046	1.0158	0.0445	14113.40	0.0102	1.0167	0.0679	
4	-0.3971	-0.0421	0.0018	1.0200	0.0446	13943.83	0.0106	1.0618	-0.0421	
5	1.2955	0.1334	0.0177	1.0031	0.0439	13957.96	0.0104	1.0374	0.1334	
6	0.6983	0.0712	0.0051	1.0159	0.0445	14012.62	0.0104	1.0440	0.0712	
7	-0.6589	-0.0689	0.0048	1.0171	0.0445	14005.02	0.0106	1.0628	-0.0689	
8	-0.9408	-0.0977	0.0096	1.0122	0.0443	14150.20	0.0106	1.0631	-0.0977	
9	-0.7407	-0.0772	0.0060	1.0158	0.0445	14134.96	0.0106	1.0611	-0.0772	
10	-0.9167	-0.0952	0.0091	1.0127	0.0443	14142.40	0.0106	1.0631	-0.0952	
11	-0.7037	-0.0735	0.0054	1.0164	0.0445	14074.70	0.0106	1.0624	-0.0735	
12	-0.2491	-0.0268	0.0007	1.0209	0.0447	14070.35	0.0106	1.0572	-0.0268	
13	0.3096	0.0310	0.0010	1.0203	0.0447	14008.19	0.0105	1.0520	0.0310	
14	-1.1760	-0.1216	0.0147	1.0069	0.0441	11975.84	0.0106	1.0639	-0.1216	
15	3.1570	0.3341	0.1019	0.9228	0.0403	13361.42	0.0104	1.0423	0.3341	*
16	0.9321	0.0954	0.0091	1.0117	0.0443	14010.58	0.0104	1.0389	0.0954	
17	2.1915	0.2274	0.0497	0.9716	0.0425	13876.21	0.0103	1.0280	0.2274	
18	-0.0767	-0.0089	0.0001	1.0211	0.0447	14146.02	0.0103	1.0341	-0.0089	
19	0.1563	0.0149	0.0002	1.0207	0.0447	14137.76	0.0103	1.0273	0.0149	
20	-1.1462	-0.1185	0.0140	1.0076	0.0441	14179.00	0.0106	1.0628	-0.1185	
21	-0.3116	-0.0328	0.0011	1.0201	0.0447	14162.90	0.0103	1.0308	-0.0328	
22	-0.9098	-0.0945	0.0090	1.0128	0.0443	13734.43	0.0106	1.0638	-0.0945	
23	0.9598	0.0982	0.0096	1.0111	0.0443	14019.04	0.0104	1.0369	0.0982	
24	-0.6917	-0.0721	0.0052	1.0165	0.0445	14153.88	0.0106	1.0579	-0.0721	

© 2018 Ozawa S et al. *JAMA Network Open.*

25	0.6045	0.0590	0.0035	1.0159	0.0445	14153.61	0.0096	0.9615	0.0590	
26	-1.1152	-0.1153	0.0133	1.0083	0.0441	14181.24	0.0106	1.0621	-0.1153	
27	-0.8785	-0.0911	0.0083	1.0134	0.0444	14173.27	0.0106	1.0581	-0.0911	
28	1.8792	0.1958	0.0373	0.9839	0.0430	13716.40	0.0104	1.0443	0.1958	
29	-0.5909	-0.0617	0.0038	1.0178	0.0446	14150.53	0.0106	1.0555	-0.0617	
30	-0.2546	-0.0274	0.0008	1.0210	0.0447	12163.02	0.0106	1.0635	-0.0274	
31	-0.7833	-0.0815	0.0067	1.0151	0.0444	14156.60	0.0106	1.0599	-0.0815	
32	-1.0347	-0.1072	0.0115	1.0102	0.0442	14178.85	0.0106	1.0625	-0.1072	
33	-0.9574	-0.0993	0.0099	1.0119	0.0443	14168.96	0.0106	1.0623	-0.0993	
34	0.4223	0.0412	0.0017	1.0181	0.0446	14154.28	0.0098	0.9798	0.0412	
35	-0.0529	-0.0063	0.0000	1.0201	0.0447	14168.08	0.0099	0.9854	-0.0063	
36	-0.5665	-0.0582	0.0034	1.0175	0.0446	14175.58	0.0102	1.0189	-0.0582	
37	-0.4761	-0.0502	0.0025	1.0193	0.0446	14017.06	0.0106	1.0615	-0.0502	
38	-1.1411	-0.1180	0.0139	1.0077	0.0441	14180.02	0.0106	1.0624	-0.1179	
39	1.4706	0.1500	0.0222	0.9981	0.0437	14048.94	0.0101	1.0131	0.1500	
40	0.0629	0.0054	0.0000	1.0210	0.0447	14140.56	0.0103	1.0304	0.0054	
41	-0.5168	-0.0539	0.0029	1.0185	0.0446	14163.87	0.0104	1.0448	-0.0539	
42	-0.6886	-0.0710	0.0051	1.0162	0.0445	14176.13	0.0103	1.0343	-0.0710	
43	0.1157	0.0109	0.0001	1.0213	0.0447	14034.83	0.0105	1.0535	0.0109	
44	-0.1490	-0.0164	0.0003	1.0212	0.0447	14114.97	0.0105	1.0500	-0.0164	
45	-0.5075	-0.0530	0.0028	1.0186	0.0446	14162.88	0.0105	1.0452	-0.0530	
46	-0.9437	-0.0975	0.0095	1.0120	0.0443	14179.36	0.0105	1.0535	-0.0975	
47	-0.5034	-0.0526	0.0028	1.0187	0.0446	14159.20	0.0105	1.0480	-0.0526	
48	-0.2637	-0.0283	0.0008	1.0209	0.0447	14015.11	0.0106	1.0596	-0.0283	
49	-0.4111	-0.0435	0.0019	1.0199	0.0446	14054.58	0.0106	1.0601	-0.0435	
50	0.6202	0.0621	0.0039	1.0165	0.0445	14123.50	0.0101	1.0121	0.0621	
51	1.2012	0.1242	0.0153	1.0056	0.0440	13818.91	0.0105	1.0487	0.1242	
52	-0.4244	-0.0442	0.0020	1.0192	0.0446	14167.52	0.0103	1.0319	-0.0442	
53	0.9028	0.0899	0.0081	1.0116	0.0443	14130.14	0.0099	0.9854	0.0899	
54	1.6100	0.1635	0.0263	0.9938	0.0435	14061.78	0.0100	1.0017	0.1636	
55	0.9250	0.0930	0.0087	1.0114	0.0443	14113.14	0.0100	1.0030	0.0930	
56	-0.7581	-0.0788	0.0062	1.0154	0.0444	14166.07	0.0106	1.0564	-0.0788	
57	0.0949	0.0087	0.0001	1.0212	0.0447	14110.08	0.0104	1.0434	0.0087	
58	2.4678	0.2571	0.0628	0.9592	0.0419	13810.86	0.0103	1.0295	0.2572	
59	-0.7461	-0.0773	0.0060	1.0155	0.0445	14172.99	0.0105	1.0496	-0.0773	
60	-1.0641	-0.1102	0.0121	1.0095	0.0442	14179.95	0.0106	1.0632	-0.1102	
61	-0.5865	-0.0613	0.0038	1.0179	0.0446	14142.94	0.0106	1.0570	-0.0613	
62	-1.1321	-0.1171	0.0137	1.0080	0.0441	14177.29	0.0106	1.0636	-0.1171	
63	0.9903	0.1008	0.0102	1.0104	0.0442	14062.18	0.0103	1.0263	0.1008	
64	-0.6215	-0.0651	0.0043	1.0176	0.0445	14023.91	0.0106	1.0624	-0.0651	
65	-0.7701	-0.0803	0.0065	1.0153	0.0444	14067.09	0.0106	1.0629	-0.0803	
66	-1.0012	-0.1038	0.0108	1.0109	0.0442	14168.10	0.0106	1.0632	-0.1038	
67	0.0303	0.0021	0.0000	1.0212	0.0447	14123.42	0.0104	1.0414	0.0021	
68	0.2522	0.0241	0.0006	1.0192	0.0447	14161.16	0.0098	0.9761	0.0241	
69	-0.6338	-0.0661	0.0044	1.0173	0.0445	14155.04	0.0106	1.0557	-0.0661	
70	0.8679	0.0889	0.0079	1.0130	0.0443	13973.26	0.0104	1.0446	0.0889	
71	0.5494	0.0548	0.0030	1.0173	0.0446	14131.49	0.0101	1.0088	0.0548	
72	-1.1662	-0.1206	0.0145	1.0071	0.0441	13969.98	0.0106	1.0639	-0.1206	
73	3.1939	0.3379	0.1041	0.9208	0.0402	13387.17	0.0104	1.0413	0.3380	*
74	1.3902	0.1441	0.0206	1.0004	0.0438	13766.23	0.0105	1.0484	0.1441	
75	-0.6247	-0.0652	0.0043	1.0174	0.0445	14147.34	0.0106	1.0572	-0.0652	
76	0.9614	0.0971	0.0094	1.0108	0.0443	14100.38	0.0101	1.0105	0.0971	
77	1.0164	0.1050	0.0110	1.0101	0.0442	13656.43	0.0105	1.0550	0.1050	
78	-0.9211	-0.0955	0.0091	1.0126	0.0443	14173.90	0.0106	1.0599	-0.0955	

© 2018 Ozawa S et al. *JAMA Network Open.*

79	0.0349	0.0025	0.0000	1.0211	0.0447	14134.88	0.0104	1.0358	0.0025	
80	1.1122	0.1151	0.0132	1.0079	0.0441	13683.54	0.0105	1.0537	0.1151	
81	0.7810	0.0778	0.0061	1.0138	0.0444	14131.17	0.0099	0.9931	0.0778	
82	0.5065	0.0512	0.0026	1.0184	0.0446	14055.76	0.0104	1.0427	0.0512	
83	0.3223	0.0313	0.0010	1.0189	0.0446	14156.64	0.0098	0.9836	0.0312	
84	-0.6300	-0.0658	0.0044	1.0174	0.0445	14147.55	0.0106	1.0574	-0.0658	
85	-1.0006	-0.1037	0.0108	1.0109	0.0442	14176.91	0.0106	1.0619	-0.1037	
86	-0.3549	-0.0368	0.0014	1.0194	0.0446	14171.61	0.0101	1.0102	-0.0368	
87	-0.7204	-0.0750	0.0057	1.0161	0.0445	14157.21	0.0106	1.0580	-0.0750	
88	-0.8442	-0.0872	0.0076	1.0138	0.0444	14177.94	0.0105	1.0468	-0.0872	
89	-1.0929	-0.1131	0.0128	1.0089	0.0441	14181.48	0.0106	1.0628	-0.1131	
90	0.3687	0.0368	0.0014	1.0196	0.0446	14096.61	0.0104	1.0378	0.0368	
91	0.1813	0.0176	0.0003	1.0209	0.0447	14087.77	0.0105	1.0458	0.0176	
92	-0.0779	-0.0091	0.0001	1.0212	0.0447	14129.50	0.0104	1.0434	-0.0091	
93	-0.6396	-0.0668	0.0045	1.0173	0.0445	14136.63	0.0106	1.0591	-0.0668	
94	3.4065	0.3627	0.1181	0.9080	0.0397	13007.91	0.0105	1.0472	0.3626	*
95	0.3135	0.0313	0.0010	1.0202	0.0447	14063.36	0.0105	1.0464	0.0313	
96	-0.4813	-0.0505	0.0026	1.0191	0.0446	14138.91	0.0105	1.0548	-0.0505	

© 2018 Ozawa S et al. *JAMA Network Open.*

3.4. Moderating Effect:

MEDQUARG Score:

```
Mixed-Effects Model (k = 96; tau^2 estimator: REML)

tau^2 (estimated amount of residual heterogeneity):     0.0447 (SE = 0.0067)
tau (square root of estimated tau^2 value):             0.2114
I^2 (residual heterogeneity / unaccounted variability): 99.92%
H^2 (unaccounted variability / sampling variability):   1184.08
R^2 (amount of heterogeneity accounted for):            0.00%

Test for Residual Heterogeneity:
QE(df = 94) = 13643.2377, p-val < .0001

Test of Moderators (coefficient(s) 2):
QM(df = 1) = 0.0234, p-val = 0.8783

Model Results:

         estimate      se    zval    pval    ci.lb   ci.ub
intrcpt    0.2368  0.0674  3.5108  0.0004   0.1046  0.3690  ***
mods       0.0017  0.0109  0.1531  0.8783  -0.0197  0.0230

---
Signif. codes:  0 ‘***’ 0.001 ‘**’ 0.01 ‘*’ 0.05 ‘.’ 0.1 ‘ ’ 1
```

Catagorical MEDQUARG (Score of greater than or equal to 6)

```
Mixed-Effects Model (k = 96; tau^2 estimator: REML)

tau^2 (estimated amount of residual heterogeneity):     0.0446 (SE = 0.0066)
tau (square root of estimated tau^2 value):             0.2112
I^2 (residual heterogeneity / unaccounted variability): 99.87%
H^2 (unaccounted variability / sampling variability):   744.10
R^2 (amount of heterogeneity accounted for):            0.00%

Test for Residual Heterogeneity:
QE(df = 94) = 13898.5432, p-val < .0001

Test of Moderators (coefficient(s) 2):
QM(df = 1) = 0.2176, p-val = 0.6409

Model Results:

         estimate      se     zval    pval    ci.lb   ci.ub
intrcpt    0.2761  0.0670   4.1185  <.0001   0.1447  0.4075  ***
mods      -0.0204  0.0438  -0.4665  0.6409  -0.1063  0.0654

---
Signif. codes:  0 ‘***’ 0.001 ‘**’ 0.01 ‘*’ 0.05 ‘.’ 0.1 ‘ ’ 1
```

© 2018 Ozawa S et al. *JAMA Network Open.*

Publication Year:

```
Mixed-Effects Model (k = 96; tau^2 estimator: REML)

tau^2 (estimated amount of residual heterogeneity):     0.0433 (SE = 0.0065)
tau (square root of estimated tau^2 value):             0.2081
I^2 (residual heterogeneity / unaccounted variability): 99.91%
H^2 (unaccounted variability / sampling variability):   1114.03
R^2 (amount of heterogeneity accounted for):            2.13%

Test for Residual Heterogeneity:
QE(df = 94) = 12804.4879, p-val < .0001

Test of Moderators (coefficient(s) 2):
QM(df = 1) = 3.0208, p-val = 0.0822

Model Results:

         estimate      se     zval    pval    ci.lb    ci.ub
intrcpt   13.2349  7.4730   1.7710  0.0766  -1.4119  27.8817  .
mods      -0.0065  0.0037  -1.7381  0.0822  -0.0137   0.0008  .

---
Signif. codes:  0 '***' 0.001 '**' 0.01 '*' 0.05 '.' 0.1 ' ' 1
```

Number of Samples Tested:

```
Mixed-Effects Model (k = 96; tau^2 estimator: REML)

tau^2 (estimated amount of residual heterogeneity):     0.0428 (SE = 0.0064)
tau (square root of estimated tau^2 value):             0.2069
I^2 (residual heterogeneity / unaccounted variability): 99.91%
H^2 (unaccounted variability / sampling variability):   1099.83
R^2 (amount of heterogeneity accounted for):            3.22%

Test for Residual Heterogeneity:
QE(df = 94) = 13802.6472, p-val < .0001

Test of Moderators (coefficient(s) 2):
QM(df = 1) = 4.1618, p-val = 0.0413

Model Results:

         estimate      se     zval    pval    ci.lb    ci.ub
intrcpt    0.2632  0.0229  11.5089  <.0001   0.2184   0.3080  ***
mods      -0.0000  0.0000  -2.0401  0.0413  -0.0000  -0.0000    *

---
Signif. codes:  0 '***' 0.001 '**' 0.01 '*' 0.05 '.' 0.1 ' ' 1
```

© 2018 Ozawa S et al. *JAMA Network Open.*

Medicine Category:

```
Mixed-Effects Model (k = 96; tau^2 estimator: REML)

tau^2 (estimated amount of residual heterogeneity):     0.0447 (SE = 0.0067)
tau (square root of estimated tau^2 value):             0.2114
I^2 (residual heterogeneity / unaccounted variability): 99.92%
H^2 (unaccounted variability / sampling variability):   1207.43
R^2 (amount of heterogeneity accounted for):            0.00%

Test for Residual Heterogeneity:
QE(df = 94) = 11855.8074, p-val < .0001

Test of Moderators (coefficient(s) 2):
QM(df = 1) = 0.0182, p-val = 0.8926

Model Results:

         estimate      se    zval    pval    ci.lb   ci.ub
intrcpt    0.2438  0.0299  8.1441  <.0001   0.1851  0.3025  ***
mods       0.0033  0.0244  0.1350  0.8926  -0.0445  0.0511


---
Signif. codes:  0 ‘***’ 0.001 ‘**’ 0.01 ‘*’ 0.05 ‘.’ 0.1 ‘ ’ 1
```

Region:

```
Mixed-Effects Model (k = 96; tau^2 estimator: REML)

tau^2 (estimated amount of residual heterogeneity):     0.0438 (SE = 0.0065)
tau (square root of estimated tau^2 value):             0.2092
I^2 (residual heterogeneity / unaccounted variability): 99.92%
H^2 (unaccounted variability / sampling variability):   1183.66
R^2 (amount of heterogeneity accounted for):            1.02%

Test for Residual Heterogeneity:
QE(df = 94) = 13665.1452, p-val < .0001

Test of Moderators (coefficient(s) 2):
QM(df = 1) = 1.9638, p-val = 0.1611

Model Results:

         estimate      se     zval    pval    ci.lb   ci.ub
intrcpt    0.3118  0.0513   6.0730  <.0001   0.2112  0.4124  ***
mods      -0.0355  0.0254  -1.4014  0.1611  -0.0852  0.0142


---
Signif. codes:  0 ‘***’ 0.001 ‘**’ 0.01 ‘*’ 0.05 ‘.’ 0.1 ‘ ’ 1
```

© 2018 Ozawa S et al. *JAMA Network Open.*

Using Random Sampling:

```
Mixed-Effects Model (k = 96; tau^2 estimator: REML)

tau^2 (estimated amount of residual heterogeneity):     0.0447 (SE = 0.0067)
tau (square root of estimated tau^2 value):             0.2114
I^2 (residual heterogeneity / unaccounted variability): 99.92%
H^2 (unaccounted variability / sampling variability):   1190.40
R^2 (amount of heterogeneity accounted for):            0.00%

Test for Residual Heterogeneity:
QE(df = 94) = 11248.9301, p-val < .0001

Test of Moderators (coefficient(s) 2):
QM(df = 1) = 0.0336, p-val = 0.8546

Model Results:

         estimate      se     zval    pval    ci.lb   ci.ub
intrcpt    0.2587  0.0699   3.7020  0.0002   0.1217  0.3957  ***
mods      -0.0080  0.0436  -0.1833  0.8546  -0.0935  0.0775

---
Signif. codes:  0 '***' 0.001 '**' 0.01 '*' 0.05 '.' 0.1 ' ' 1
```

Using Mystery Shoppers:

```
Mixed-Effects Model (k = 96; tau^2 estimator: REML)

tau^2 (estimated amount of residual heterogeneity):     0.0438 (SE = 0.0065)
tau (square root of estimated tau^2 value):             0.2094
I^2 (residual heterogeneity / unaccounted variability): 99.85%
H^2 (unaccounted variability / sampling variability):   686.48
R^2 (amount of heterogeneity accounted for):            0.88%

Test for Residual Heterogeneity:
QE(df = 94) = 13425.9920, p-val < .0001

Test of Moderators (coefficient(s) 2):
QM(df = 1) = 1.8334, p-val = 0.1757

Model Results:

         estimate      se     zval    pval    ci.lb   ci.ub
intrcpt    0.3414  0.0733   4.6561  <.0001   0.1977  0.4851  ***
mods      -0.0595  0.0440  -1.3540  0.1757  -0.1457  0.0266

---
Signif. codes:  0 '***' 0.001 '**' 0.01 '*' 0.05 '.' 0.1 ' ' 1
```

© 2018 Ozawa S et al. *JAMA Network Open.*

eTable 2: Descripitive Statistics of All Studies in Systematic Review

**Sample Size**				**Sample Size Statistics**		
	n	%		Mean	1512	
<10	56	21.1%		Median	42	
10-50	85	32.1%		Total	400708	
50-100	21	7.9%		Min	2	
100-200	27	10.2%		Max	283233	
200-500	41	15.5%		IQR	10 - 242	
500-1000	15	5.7%				
1000-10000	15	5.7%		**Year Published**		
>10000	5	1.9%			n	%
Total	265			Before 2000	19	7.2%
				2000-2010	89	33.6%
**Medicine Type**				2010-2015	94	35.5%
	n	%		After 2015	63	23.8%
Anti-retrovirals Only	8	3.0%		Total	265	100%
Analgesic Only	23	8.7%				
**Antibiotics Total**	**104**	39.2%				
Only Antibiotics	58	21.9%		**Region**		
Antibiotics and Others[a]	11	4.2%			n	%
**Antimalarials Total**	**117**	44.2%		Africa	133	50.2%
Only Antimalarials	82	30.9%		Asia	90	34.0%
**Antimalarials and Antibiotics Total**	**35**	13.2%		S America	8	3.0%
Only Antimalarials and Antibiotics	21	7.9%		Europe	3	1.1%
Antimalarials, Antibiotics, and Others[a]	14	5.3%		N America	3	1.1%
Cardiovascular Only	15	5.7%		Oceania	3	1.1%
Multiple Other[b]	33	12.5%		Multiple	25	9.4%
Total	265	100.0%		Total	265	100.00 %

a: Medicines classified as other include: acid blockers, antacids, anthelmintics, anti-anemics, antimycobacterials, antifungals, anti-hypertensives, anti-inflammatories, antiretrovirals, bronchodilators, erectile dysfunction drugs, diuretics, spasmolytics, steroids, etc
b: Medicine catagories were classified as multiple other if they examined multiple medicine catagories but did not include antibiotics or antimalarials.

© 2018 Ozawa S et al. *JAMA Network Open.*

eFigure 4: Reported Prevalence of Substandard and Falsified Drugs in Studies in Africa

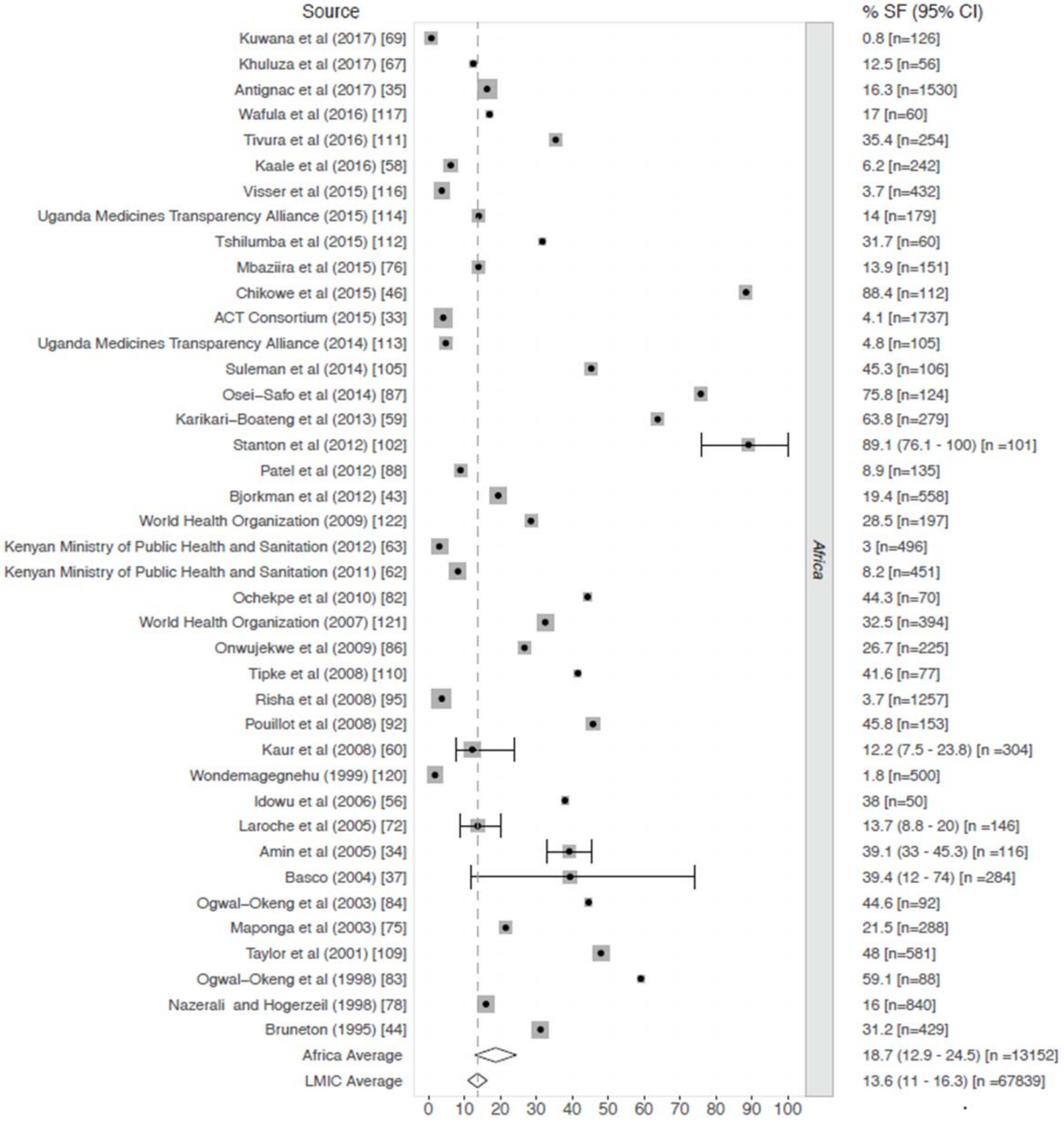

A forest plot of the reported prevalence of poor quality medicines for studies conducted in Africa that were included in the meta-analysis. Mean weighted regional and overall prevalences are reported as diamonds with a 95% confidence interval.

The average overall prevalence of substandard and falsified medicines was 13.6% across LMICs (95% CI: 11.0% - 16.3%). Regional prevalence estimates were 18.7% in Africa (12.9% - 24.5%).

© 2018 Ozawa S et al. *JAMA Network Open.*

eFigure 5: Reported Prevalence of Substandard and Falsified Drugs in Studies in Asia

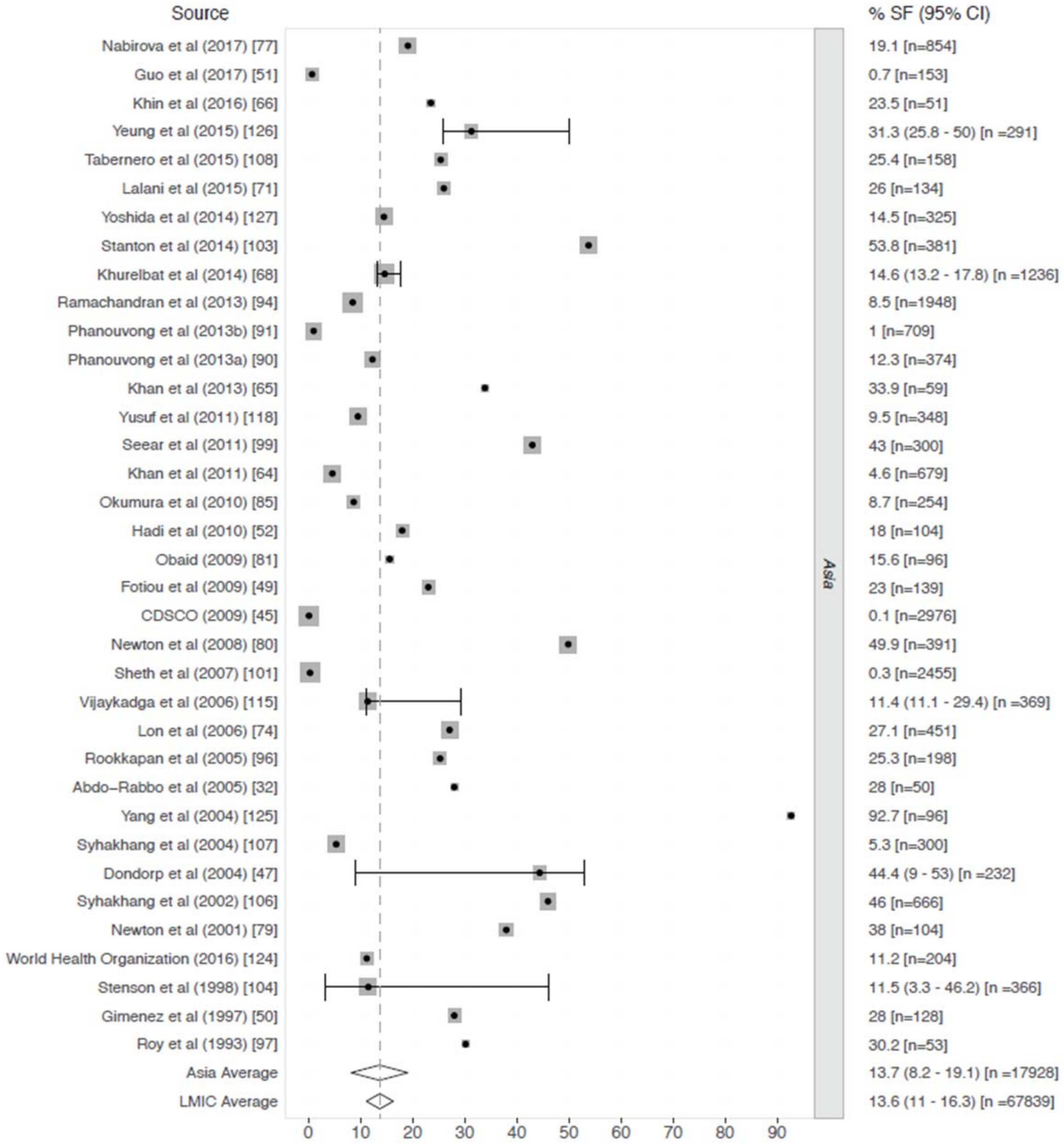

A forest plot of the reported prevalence of poor quality medicines for studies conducted in Asia that were included in the meta-analysis. Mean weighted regional and overall prevalences are reported as diamonds with a 95% confidence interval.

The average overall prevalence of substandard and falsified medicines was 13.6% across LMICs (95% CI: 11.0% - 16.3%). Regional prevalence estimates were 13.7% in Asia (8.2% - 19.1%).

© 2018 Ozawa S et al. *JAMA Network Open.*

eFigure 6: Reported Prevalence of Substandard and Falsified Drugs in Studies that Examined Multiple Regions

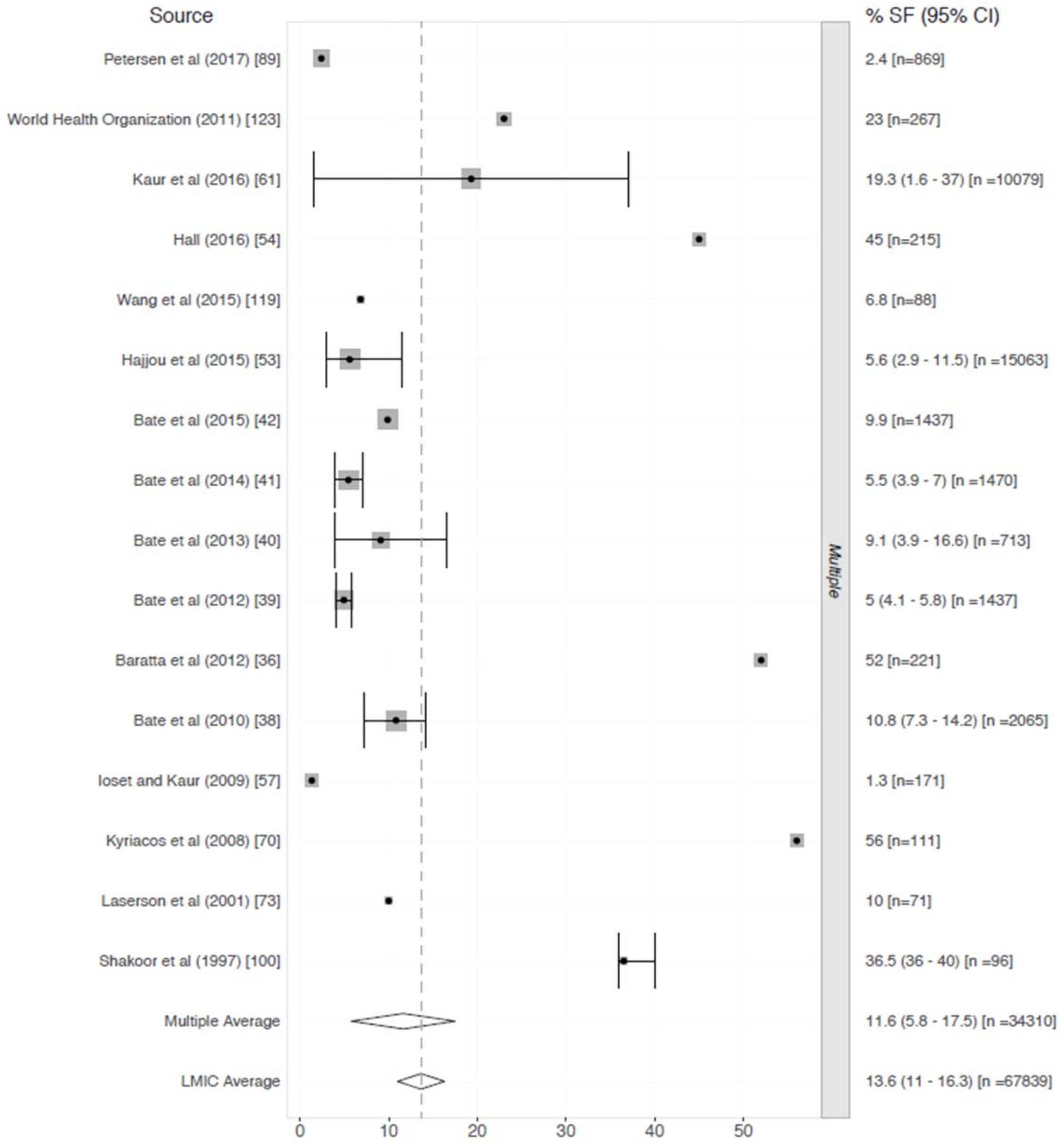

A forest plot of the reported prevalence of poor quality medicines for studies conducted in multiple regions that were included in the meta-analysis. Mean weighted regional and overall prevalences are reported as diamonds with a 95% confidence interval.

The average overall prevalence of substandard and falsified medicines was 13.6% across LMICs (95% CI: 11.0% - 16.3%). Prevalence estimates were 11.6% in multiple region studies (5.8% - 17.5%).

© 2018 Ozawa S et al. *JAMA Network Open.*

eFigure 7: Reported Prevalence of Substandard and Falsified Drugs in Studies in Other Regions (South America, Europe, and Oceania)

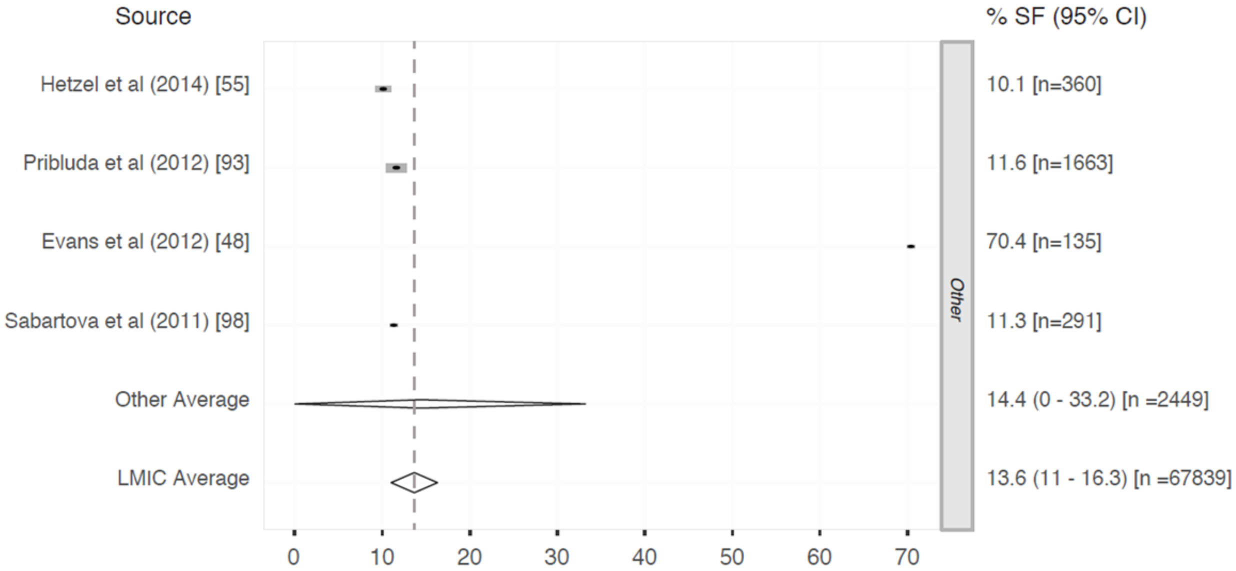

A forest plot of the reported prevalence of poor quality medicines for studies conducted in other regions (South America, Europe, and Oceania) that were included in the meta-analysis. Mean weighted regional and overall prevalences are reported as diamonds with a 95% confidence interval.

The average overall prevalence of substandard and falsified medicines was 13.6% across LMICs (95% CI: 11.0% - 16.3%). Regional prevalence estimates were 14.4% for other single region studies (0% - 32.2%).

© 2018 Ozawa S et al. *JAMA Network Open.*

eTable 3: Estimates of the Economic Impact of Substandard and Falsified Medicines

Estimated Annual Economic Impact	Impact Definition	Scope	S/F Category	Medicines	Original Source	Source type
USD 200 billion	Estimated market size	Global	Falsified	All	World Economic Forum (2011)[3]	WEF Report, Global Risks 2011 6th edition
USD 75 billion	Revenue in 2010	Global	Falsified	All	Pitts (2005)[4] Author's calculation	Moderator's guide from Center for Medicines in the Public Interest
USD 35 billion	Annual sales of drugs	Global	Falsified	All	Cockburn (2005)[5] Author's calculation	Ballpark estimate in peer reviewed article
USD 32 billion	Annual earnings from sales.	Global	Falsified	All	World Health Organization (2003)[6]	WHO Factsheet[a]
USD 30.5 billion	Market size	LMICs	Substandard and Falsified	All	World Health Organization (2017)[7] calculation	WHO Report
USD 20 billion	Annual pharmaceutical industry financial loss	Global	Falsified	All	Ten Ham (2003)[8] Author's calculation	Ballpark estimate in peer reviewed article
USD 18-34 billion	Market size	Global	Falsified	All	Anisfeld (2006)[9] Author's calculation	Ballpark estimate in peer reviewed article
USD 10 billion	Market size	Global	Falsified	All	Bate (2014)[10] Author's calculation	Ballpark estimate in book

a : This estimate is no longer used in later versions of WHO factsheets

FBI: Federal Bureau of Investigation; IFPMA: International Federation of Pharmaceutical Manufacturers & Associations; LMICs: Low and Middle-Income Countries; UNODC: United Nations Office of Drugs and Crime; USD: United States Dollars; WEF: World Economic Forum; WHO: World Health Organization.

© 2018 Ozawa S et al. *JAMA Network Open.*

eReferences 2: eAppendix References

1. Newton PN, Lee SJ, Goodman C, et al. Guidelines for Field Surveys of the Quality of Medicines: A Proposal. *PLoS medicine.* 2009;6(3):e1000052.
2. Almuzaini T, Choonara I, Sammons H. Substandard and counterfeit medicines: a systematic review of the literature. *BMJ open.* 2013;3(8):e002923.
3. World Economic Forum. *Global Risks 2011 Sixth Edition.* Geneva, Switzerland: World Economic Forum;2011.
4. Pitts P. *21st Century Health Care Terrorism: The Perils of International Drug Counterfeiting - Moderator's guide.* Washington, DC: Center for Medicines in the Public Interest, Pacific Research Institute;2005.
5. Cockburn R. Crime, fear and silence: Making public the fake pharmaceutical drug racket [presentation]. First global forum on pharmaceutical anticounterfeiting; 2002.
6. World Health Organization. *Substandard and counterfeit medicines.* Geneva, Switzerland: World Health Organization;2003.
7. World Health Organization. *A study on the public health and socioeconomic impact of substandard and falsified medical products.* Geneva2017.
8. Ten Ham M. Health Risks of Counterfeit Pharmaceuticals. *Drug Safety.* 2003;26(14):991-997.
9. Anisfeld MH. Counterfeit pharmaceuticals and the International Pharmaceutical Federation (FIP) working group on counterfeit medicines. *Journal of Pharmacy Practice.* 2006;19(3):178-181.
10. Bate R. *Phake: The Deadly World of Falsified and Substandard Medicines.* Lanham, MD and Boulder: Rowman and Littlefield, AEI Press; 2014.

© 2018 Ozawa S et al. *JAMA Network Open.*
